# Bioinspired strong and rigid fusion-spun graphene fibers for harsh robotics

**DOI:** 10.1126/sciadv.aeg6444

**Published:** 2026-09-04

**Authors:** Yiwei Zhang, Senping Liu, Dan Chang, Bo Wang, Xin Ming, Jiahao Lu, Lidan Wang, Peng Li, Chendong Ge, Yue Gao, Yingjun Liu, Zhen Xu, Chao Gao

**Affiliations:** MOE Key Laboratory of Macromolecular Synthesis and Functionalization, Zhejiang Key Laboratory of Advanced Organic Materials and Technologies, International Research Center for X Polymers, Research Center for Advanced Fibers, Department of Polymer Science and Engineering, Zhejiang University, Hangzhou 310058, China.

## Abstract

High-performance fibers with the merits of lightweight and high strength are indispensable to modern industry. However, the performance trade-off between tensile strength and bending rigidity limits their more applicational possibilities as structural materials. Inspired by the hierarchical assembly of biomaterials, we exploit graphene fibers through multiscale fusion spinning of gel fiber bundles with an unprecedented synergy of high tensile strength of 3.0 gigapascal and bending rigidity reaching 1.6 × 10^6^ newtons per square micrometer for a 100-micrometer-thick fiber (more than 10^4^ times higher than Kevlar fibers). Continuous fusion–induced defect suppression produces thick fibers with homogeneous ordering and performance. These fibers also exhibit exceptional thermal and electrical conductivities, showing their potential as structure-function integrated materials. The fibers are used as wing veins, feet, or claws of robots, resisting extreme conditions including wind speed from 0 to 7 Beaufort scale, pH from 0 to 14, and temperature from 4 to 2000 kelvin. These results promise structural materials design, bionic functions, and robotics in harsh environments.

## INTRODUCTION

High-performance fibers are crucial for modern industries and technologies, such as aerospace vehicles, construction engineering, and biorobotics (*1*–*4*). Continuous improvements in the mechanical properties of these fibers, including carbonaceous fibers, polymer fibers, and ceramic fibers, have driven the technological revolutions (*5*–*8*). However, the current strategies for enhancing tensile strength generally result in the dramatically reduced fiber diameter, leading to a substantial decrease in the bending rigidity (typically less than 4 × 10^3^ N μm^2^, bending rigidity *B = EπD^4^*/*64*, where *D* is fiber diameter, and *E* is elastic modulus) (*9*–*11*). For example, the bending rigidity of carbon fibers decreases from 27 N μm^2^ (T300, TORAY) to 10 N μm^2^ (T1100G, TORAY) with increasing tensile strength. The same trend also applies to graphene fibers: Higher tensile strength is achieved at the expense of bending rigidity reduction due to the decrease of fiber diameter (fig. S1 and table S1) (*10*, *12*–*17*). The excessively thin fibers easily deform or even fracture under lateral stress, failing to maintain the structural and performance stabilities under complex stress environments, thereby limiting their more application possibilities as structural materials. To simultaneously improve both tensile strength and bending rigidity, an effective approach is increasing fiber diameter while maintaining high strength and modulus. However, constrained by Griffith’s law, the tensile strength and elastic modulus of fibers exponentially decrease with elevating fiber diameter due to the dramatic increase of defect size (*10*, *18*–*23*). This conflict leads to a huge problem to prepare fibers with both high tensile strength and bending rigidity. Several approaches were proposed to mitigate the conflict, such as resin impregnation of fiber bundles (*24*), improving draw ratio of large-diameter fibers (*25*), filling structural defects with small-size ingredients (*6*), enhancing shearing orientation by microfluidic channels (*26*), etc. The resultant fibers generally show heterogeneous axial and radial morphologies, as well as limited enhancement of strength and rigidity. In addition, the incorporation of polymer severely sacrifices the inherent thermal stability or thermal/electrical conductivity of fibers. The preparation of strong and rigid or thick fibers still faces a great challenge.

**Fig. 1.**
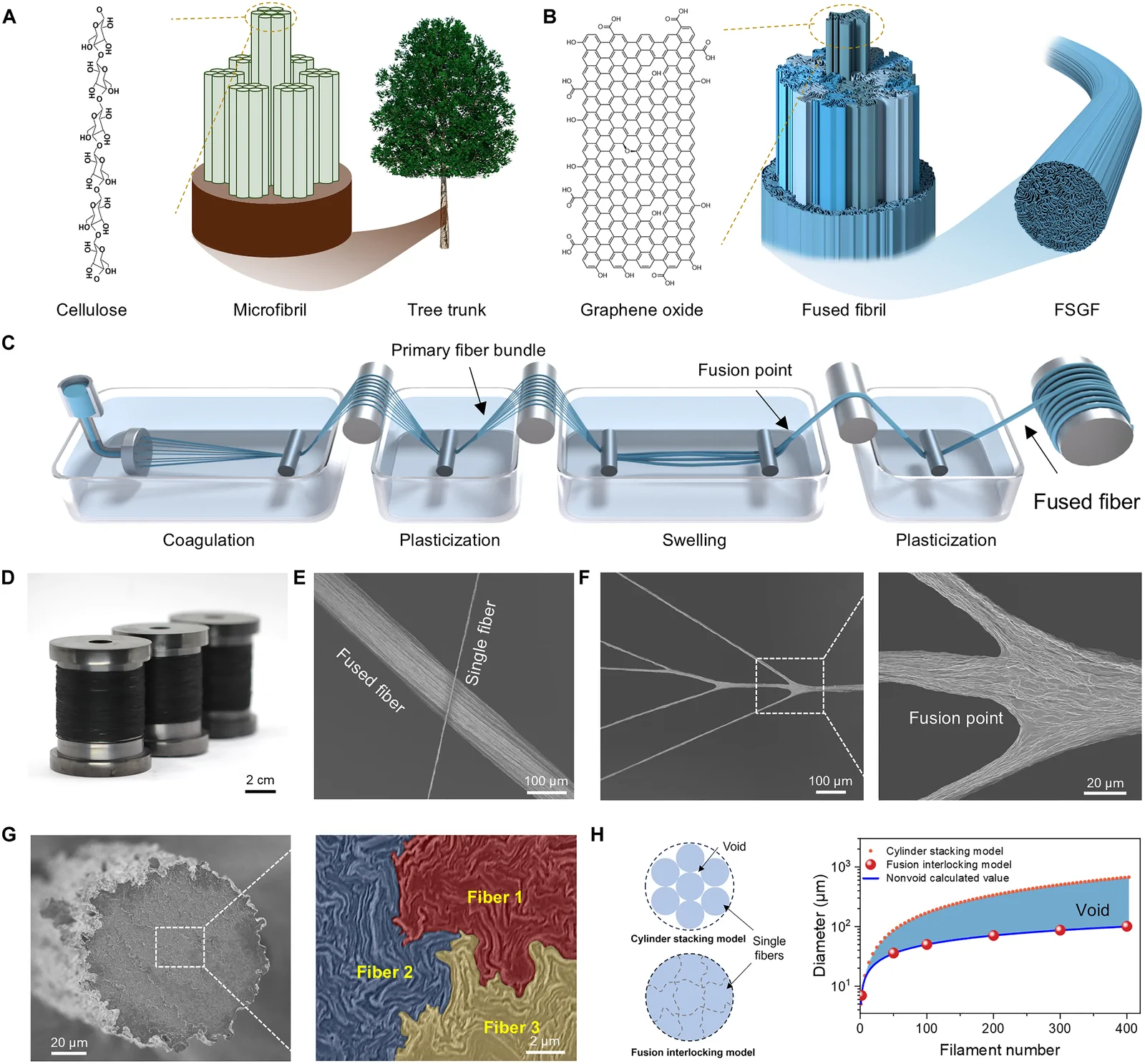
Biohierarchy-inspired fabrication of FSGFs. (**A**) Schematic demonstrating the cellulose hierarchical assembly of tree trunk. (**B**) Schematic illustration of the fusion-induced defect suppression strategy of FSGFs prepared by multiscale fusion spinning. (**C**) Schematic of the continuous multiscale fusion spinning process. (**D**) Photograph showing the FSGFs prepared by multiscale fusion spinning. (**E**) SEM image of 100-μm-thick FSGOF fused by 400 filaments and a single 5-μm-thick filament. (**F**) SEM images depicting the fusion process of multiple GO filaments (left) and the enlarged region of fusion point (right). (**G**) SEM images showing the dense and regular cross section of fusion-spun GO fibers (left), as well as the magnified region highlighting the interlocking structure among filaments (right). (**H**) Schematic illustrating the cylindrical stacking model and the fusion interlocking model of multiple filaments (left), as well as the diameter of FSGF versus the filament number during the multiscale fusion spinning process (right). Illustrations were created by the authors as follows: (A) using Microsoft PowerPoint; (B) and (C) using Cinema 4D.

In nature, structural biomaterials such as tree trunks and muscle fibers maintain their mechanical performance across orders-of-magnitude increases in size (*27*, *28*). This size-insensitive mechanical behavior arises from the hierarchical assembly principle, which organizes matter progressively from molecules to microfibrils and ultimately to macrostructures (Fig. 1A and fig. S2) (*29*). Compared to direct molecular assembly, the hierarchical assembly effectively limits the growth of defects as material dimensions increase, thereby avoiding stress concentration and preventing the rapid degradation of mechanical properties.

Inspired by the hierarchical assembly of biomaterials, here we propose a fusion-induced defect suppression strategy to overcome the long-standing strength-rigidity conflict in fibers (Fig. 1B). During the multiscale fusion spinning process, a continuous fusion and plasticization allowed numerous graphene oxide (GO) gel filaments to fuse and orient into a single thick fiber with high structural regularity. After thermal reduction, the fusion-spun graphene fibers (FSGFs) realized a remarkable combination of high tensile strength reaching 3.0 GPa and bending rigidity of up to 1.6 × 10^6^ N μm^2^ (more than 10^4^ times higher than Kevlar fibers), overcoming the traditional limitation of Griffith’s law. These FSGFs also exhibited exceptional thermal conductivity of 1390 W m^−1^ K^−1^, electrical conductivity of 1.2 × 10^6^ S m^−1^, and property homogeneity with SD below 2%. The fusion-induced defect suppression strategy was also extended to prepare various strong and rigid composite fibers. The fibers were used as wing veins, claws, and feet of robots, which maintained the performance under extreme conditions of high wind from 0 to 7 Beaufort scale, strong acid and base with pH from 0 to 14, and extreme high and low temperature from 4 to 2000 K. This achievement opens an avenue for developing high-performance structural materials, harsh-environment robotics, and biomimetic functions.

## RESULTS

### Continuously fabricate FSGFs by multiscale fusion spinning

GO, a representative two-dimensional macromolecule, exhibits large conformational transformation, easy processability and assimilability, and self-adhesion properties (*30*–*33*). These traits make GO a molecular model for studying assembly behaviors and material properties. On the basis of the large swelling and shrinkage characteristics of GO-based materials, the discovery of their precisely reversible assembly phenomenon offers great possibilities for constructing macroscopic structures (*34*, *35*). In this work, we propose a fusion-induced defect suppression strategy to continuously fabricate graphene fibers via multiscale fusion spinning of GO (Fig. 1C and figs. S3 and S4). Specifically, a spinneret with multiple holes from 2 to 400 with each hole diameter of 80 μm was used to produce multiple GO gel filaments. The individual hole size of 80 μm was selected as it provides an optimal balance between spinning stability and the mechanical performance of the resulting FSGFs (fig. S5). The real-time continuous fusion spinning process was provided using a 100-hole spinneret at a collection speed of 2 m min^−1^ (movie S1). Through proper adjustment of the spinning solution rheology and preparation parameters (e.g., solution concentration and quality to avoid large solid particles), stable spinning without clogging or breakage was achieved (fig. S6). After coagulation and plasticization stretching, multiple orienting gel filaments were swollen by a swelling bath of water and ethanol with swelling ratio as high as 600% (fig. S7). As the filaments were drawn out and dried, the multiple swollen filaments continuously fused into a single thick fiber (fig. S8). The fused fiber was further stretched through a plasticization bath and then successively collected on a reel. The total draw ratio of fibers was 75%. After heat treatment (*36*, *37*), FSGFs were obtained (Fig. 1D and fig. S9). In the fusion stage, the multiple soft swollen filaments contact and synergistically shrink through adaptive deformation of GO sheets under capillary force. Consequently, multiple swollen filaments fuse into a dense thick fiber via interlocking wrinkles and spontaneous interfacial noncovalent interactions of hydrogen bonding, van der Waals force, and π-π interaction (Fig. 1, E and F, and fig. S10) (*38*). Direct evidence of this adaptive fusion mechanism is provided by tracing the fusion process of two labeled filaments as well as filament bundles using scanning electron microscopy–energy dispersive x-ray spectroscopy (SEM-EDS) (figs. S11 and S12). Compared with traditional direct wet-spun graphene fibers (DSGFs), FSGF show a more round and uniform cross-sectional morphology and structure (Fig. 1G and fig. S13). By using the fusion spinning strategy, the heterogeneous core-shell structure of fibers was obviously eliminated.

By altering the hole number of spinneret, the diameter of FSGF was easily adjusted from 7 to 100 μm (fig. S14). Different from the simple stacking model of cylinders, the multiple filaments deform to form dense interlocking interfaces in the FSGFs. Therefore, the relationship between the diameter of FSGF (*D*) and the filament diameter (*d*) and number (*n*) can be hypothesized as $D=d\sqrt{n}$ (see Supplementary Text). To verify this hypothesis, FSGFs with different diameters were prepared by changing the hole number of spinneret. The corresponding diameter of a single dry filament is 5 μm. As a result, with the hole number increasing from 2 to 400, the diameter of FSGF elevated from 7 to 100 μm (Fig. 1H). This rule aligns with the theoretical formula, validating the dense interlocking structure of fibers. The ridge width of 5 μm in fusion-spun graphene oxide fibers (FSGOFs) is notably narrower than the apparent width of 20 μm for a single dry GO filament, indicating that the ridges are formed during fusion process rather than being individual fibers simply brought together in a bundle (fig. S15).

### Structural regularity

**Fig. 2.**
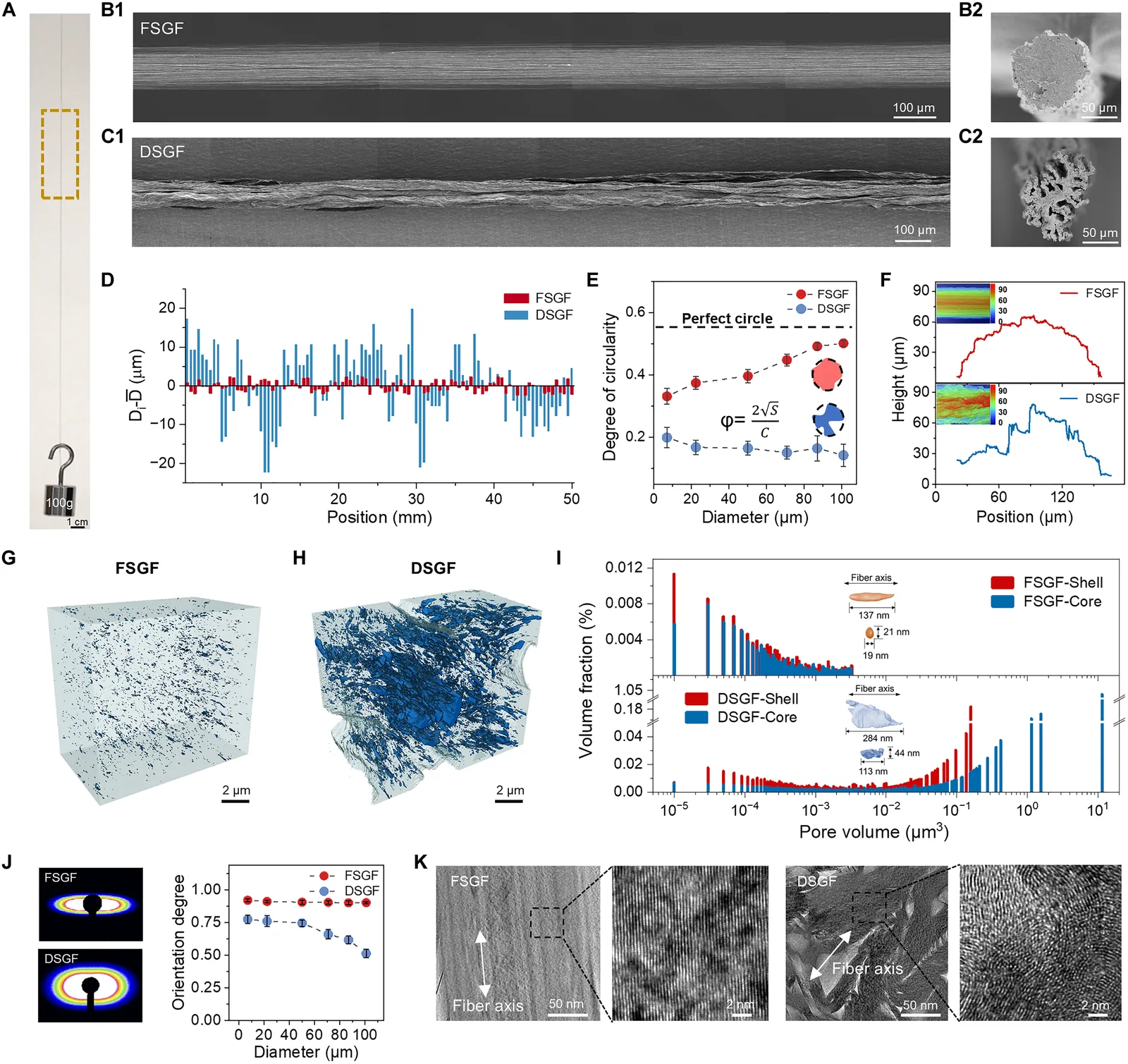
Structural regularity of FSGFs across multiple scales. (**A**) Digital photograph showing the meter-long FSGF with 100 μm in diameter bearing a 100-g weight. (**B1** and **B2**) Scanning electron microphotographs suggesting the lateral view (B1) and cross-sectional view (B2) of 100-μm-thick FSGF. The fiber demonstrates axial uniformity and radial regularity. (**C1** and **C2**) Scanning electron microphotographs showing the lateral view (C1) and cross-sectional view (C2) of 100-μm-thick DSGF. The fiber exhibits severe axial heterogeneity and radial irregularity. (**D**) Statistical analysis of the fiber diameter deviation from the average diameter at different axial positions. (**E**) Circularity of FSGF and DSGF versus the corresponding diameter. (**F**) Surface height profile of FSGF and DSGF measured by white-light interference microscopy. The insets show the corresponding topography of FSGF (top) and DSGF (bottom), respectively. (**G** and **H**) FIB-SEM three-dimensional reconstructions showing the microstructure of FSGF (G) and DSGF (H), respectively. The dark blue regions represent the void defects. (**I**) Statistical pore volume distributions within the center and periphery of FSGF (top) and DSGF (bottom), respectively. (**J**) Typical SAXS patterns of FSGF and DSGF with diameters of 100 μm (left) and calculated orientation degree (*f*) with varying fiber diameter (right). (**K**) TEM images showing the crystalline structures of FSGF and DSGF with diameters of 100 μm along the fiber axis.

In comparison with the graphene fibers prepared by common wet spinning approach, the FSGFs with the same diameter show superior structural regularity. The structural advantages were revealed from axial to radial directions at multiple scales. In the axial direction of fibers, FSGFs exhibit a more homogeneous morphology than DSGFs of the same diameter (Fig. 2, A to C, and fig. S16). Specifically, along a 50-mm-long fiber, the fiber diameter is measured at intervals of 3 μm. As the fiber diameter increases from 7 to 100 μm, the standard deviation of diameter at these measured positions of FSGF remains below 3 μm, while that of DSGF dramatically rises from 4 to 26 μm (fig. S17). In the radial direction of fibers, FSGF show a rounder transversal morphology than DSGF. A degree of circularity φ was calculated to quantify the transversal shape of fibers based on the perimeter *C* and area *S* in fiber cross section as $\varphi =2\sqrt{S}/C$. The closer the φ is to 0.56, the closer the fiber cross section is to a perfect circle. As fiber diameter increases from 7 to 100 μm, the φ of FSGF rises up to 0.51, whereas that of DSGF declines to merely 0.14 (Fig. 2E). The higher degree of circularity of FSGF demonstrates a better morphological regularity along the radial direction by using multiscale fusion spinning strategy. In terms of the white light interferometry characterizations, the surface of FSGF is rounder and smoother than that of DSGF. The surface roughness of FSGF is only 2.5 μm, much smaller than 7.5 μm of DSGF (Fig. 2F and fig. S18). The fibers prepared by multiscale fusion spinning suggest a better structural regularity in both axial and radial directions.

In addition, the FSGFs exhibit an excellent structural regularity from macroscale to nanoscale. Benefiting from the dense stacking of graphene sheets, the density of macroscale FSGF is significantly higher than that of DSGF. With increasing fiber diameter from 7 to 100 μm, the density of FSGF remains around 1.94 g cm^−3^ (fig. S19). The consistent density reveals the dense structures of fibers with different diameters. By contrast, the density of DSGF significantly decreases from 1.83 to 1.62 g cm^−3^ with rising fiber diameter. The sharp density decline is attributed to the more severe core-shell structure caused by elevating double-diffusion path in wet spinning as fiber diameter increases. To verify the speculation, the 100-μm-thick GO fibers respectively prepared by multiscale fusion spinning and traditional wet spinning strategies were water-swollen and then freeze-dried to observe the cross-sectional structures. As shown in the SEM characterizations, the fusion-spun fibers appear homogeneous interior sheet structure after swelling (fig. S20). While the traditional wet-spun swollen fibers suggest a severe core-shell structure with a huge hole in the core. This inhomogeneity stems from the loosely stacked sheets in the core of wet-spun fibers.

At the microscale, the difference in the microstructures of graphene fibers fabricated by multiscale fusion spinning and direct wet spinning was further analyzed by focused ion beam–SEM (FIB-SEM) (*16*). According to the three-dimensional microstructural reconstruction, the FSGFs display similar porosity of 0.116 and 0.105% within center and exterior, respectively. Yet, the DSGFs show notably higher porosity of 2.35% within the center than that of 1.38% within the exterior, indicating a severe core-shell structure (Fig. 2, G and H, and figs. S21 and S22). The average pore volume of FSGF within the center is 7.3 × 10^−4^ μm^3^, almost four orders of magnitude smaller than 6.2 μm^3^ of DSGF (Fig. 2I). The spatial size of a typical void for FSGF is 19 nm in width, featuring an oblate shape, while the void width for DSGF is as large as 113 nm, appearing an irregular shape. By using multiscale fusion spinning strategy, the core-shell structure of fibers, especially large-diameter fibers, was eliminated to greatly improve the structural regularity.

At a finer scale, the nanoscale sheet orientation and crystallization structures of FSGFs were investigated. Both small-angle x-ray scattering (SAXS) and wide-angle x-ray scattering (WAXS) were carried out to examine the orientation of graphene sheets of fibers (*39*). With elevating fiber diameter from 7 to 100 μm, the orientation degree of DSGF sharply decreases from 0.77 to 0.51 because of the aggravating core-shell nonuniformity (Fig. 2J and fig. S23). By contrast, the orientation degree of FSGF remain above approximately 0.9 as diameter increased. The FSGF also retains the high atomic orientation. The WAXS orientation degree of FSGF is 0.93, much higher than 0.65 of DSGF (fig. S24). In addition, the high-resolution transmission electron microscopy (HR-TEM) revealed the graphitic lamellar crystallite along the fiber axis with an interlayer spacing of 0.335 nm of FSGF (Fig. 2K and fig. S25). However, the DSGF exhibits a turbulent lamellar stacking of graphene with an interlayer spacing of 0.35 nm. Cross-sectional HR-TEM further confirms that FSGF displays a dense, folded crystalline-lamella morphology without observable interfacial gaps or microcracks, while DSGF contains numerous irregular voids (figs. S26 and S27). In the case of fusion-spun fibers, both of the orientation and crystalline nanostructures were largely optimized. The multiscale fusion spinning strategy substantially limits the growth of defects as fiber diameter increases. This fusion-induced defect suppression mechanism arises from two aspects: the shortened double diffusion path that prevents large pore defects and core-shell structures, and the decrease of uneven surface wrinkle defects through fusion to yield a uniform and circular cross section.

### High tensile strength and bending rigidity with performance uniformity

**Fig. 3.**
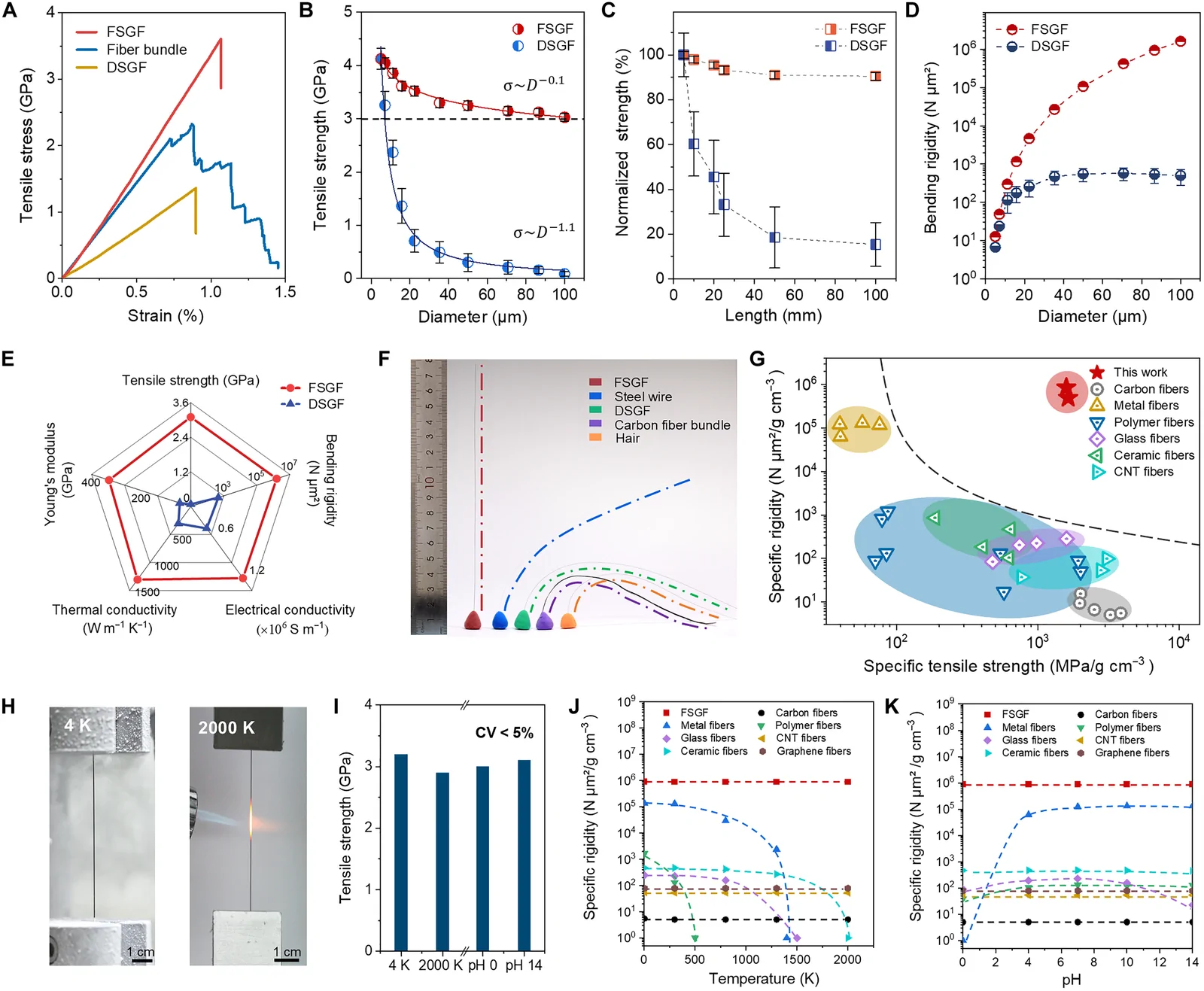
Comprehensive properties and property resistance to extreme environments of FSGFs. (**A**) Tensile curves of FSGF, nonfused graphene fiber bundles, and DSGF. The diameter of FSGF and DSGF is 20 μm. (**B**) Changes in the tensile strength of FSGF and DSGF with increasing fiber diameter. (**C**) Variations of the tensile strength of FSGF and DSGF with increasing gauge length in tensile tests. (**D**) Bending rigidity of FSGF and DSGF as a function of fiber diameter. (**E**) Radar chart showing the mechanical properties as well as thermal and electrical conductivities of FSGF and DSGF. (**F**) Self-supporting digital photographs of FSGF and other types of fibers (the type of carbon fiber bundle is T800). (**G**) Ashby plot of specific bending rigidity and specific tensile strength of different fibers. (**H**) Photographs of FSGF during the in situ tensile tests under 4 K (left) and 2000 K (right), respectively. (**I**) Tensile strength of 100-μm-thick FSGF under extreme temperatures (4 and 2000 K) and chemical conditions (pH 0 and pH 14). (**J**) Specific bending rigidity changes of FSGF and common fibers with varying temperatures ranging from 4 to 2000 K. (**K**) Specific bending rigidity changes of FSGF and common fibers with different pH ranging from 0 to 14.

Because of the structural regularity, the FSGFs exhibit outstanding and uniform mechanical properties. On the one hand, the 20-μm-thick FSGFs fused by 20 filaments show an outstanding tensile strength of up to 3.6 GPa, notably higher than 1.4 GPa of similar thick wet-spun graphene fibers (Fig. 3A). As fiber diameter *D* increases from 7 to 100 μm, the tensile strength σ of FSGF maintains above 3.0 GPa, whereas that of DSGF dramatically reduces to 87 MPa (Fig. 3B). The rapid decline in mechanical performance indicates the high size sensitivity of DSGF. In comparison with σ ∼ *D*^−1.1^ for DSGFs, the exponential decline was nearly eliminated for FSGFs as σ ∼ *D*^−0.1^. Moreover, with elevating test length from 5 to 100 mm, the tensile strength of FSGF did not obviously decrease while that of DSGF sharply decreased by 85% (Fig. 3C). Weibull analysis further shows that the Weibull modulus of FSGF increases with fiber diameter (from 7.55 at 7 μm to 9.96 at 100 μm), different from the conventional decrease trend due to size effect (figs. S28 and S29). The elongation at break of FSGF is 0.9 to 1.2%, similar to that of typical high-performance carbon fibers, such as polyacrylonitrile (PAN)–based (e.g., TORAYCA T300 series) and pitch-based (e.g., Mitsubishi Chemical K13312) carbon fibers. The persistent tensile strength of FSGFs breaks through the Griffith’s law limitation, opening an avenue for developing high-performance fibers (*18*, *40*). On the other hand, the FSGFs simultaneously exhibit a high bending rigidity due to the combination of high elastic modulus and large fiber diameter. On the basis of three-point bending tests (*41*, *42*), the bending rigidity of 100-μm-thick FSGF exceeds that of DSGF by more than four orders of magnitude (Fig. 3D). Compared with 100-μm-thick DSGF, the FSGF with the same diameter suggests higher tensile strength of 3.0 GPa, Young’s modulus of 330 GPa, bending rigidity of 1.6 × 10^6^ N μm^2^, thermal conductivity of 1390 W m^−1^ K^−1^, and electrical conductivity of 1.2 × 10^6^ S m^−1^ (Fig. 3E and figs. S30 to S32). The thermal conductivity of FSGF significantly outperforms that of common metal fibers with similar diameter, such as copper fibers, aluminum fibers, and steel fibers. This advantage was demonstrated by the ice insertion experiments, where the inserting time of FSGF was notably shorter than metal fibers (fig. S33 and movie S2).

Overall, the 100-μm-thick FSGFs exhibit both high tensile strength of 3.0 GPa and bending rigidity of 1.6 × 10^6^ N μm^2^, overcoming the long-term strength-rigidity conflict. Owing to the lightweight merit, the fibers show a combination of ultrahigh specific tensile strength of 1.6 × 10^3^ MPa g^−1^ cm^3^ and specific bending rigidity of up to 8.5 × 10^5^ N μm^2^ g^−1^ cm^3^ (Fig. 3, F and G, and figs. S34 and S35) (table S2). The ultrastrong and ultrarigid fibers fill the blank of conventional high-performance fibers, including the metal fibers with high rigidity but low strength, carbon fibers, carbon nanotube fibers, glass fibers, ceramic fibers, and polymer fibers with high strength but low rigidity (*2*, *5*, *8*, *43*–*45*). Meanwhile, these properties, combined with its exceptional thermal conductivity, render it the fiber with the highest specific bending rigidity and thermal conductivity among existing materials (fig. S36). The in situ Raman measurements during the tensile and bending processes indicate a more intense red or blue shift in G peak of FSGF compared to DSGF, demonstrating a stronger sheet interaction within the FSGF (figs. S37 to S39). Furthermore, no delamination was observed on the fracture surfaces of the FSGFs under different loading modes, further attesting to the tightly interlocked arrangement of the graphene-based sheets (fig. S40). Notably, the multiscale fusion spinning approach was further extended to prepare diverse strong and rigid composite fibers. After incorporating 50% GO, the tensile strength of PAN-based, polyvinyl alcohol (PVA)–based, and sodium alginate (SA)–based composite fibers was well maintained with increasing fiber diameter (figs. S41 and S42). The performance of FSGFs remains stable in various extreme environments, including temperatures from 4 to 2000 K as well as strong acid and base with pH ranging from 0 to 14 (*46*, *47*). Raman spectroscopy shows that the *I*_*D*_*/*I**_*G*_ of FSGF remains as low as 0.06 after exposure to 4 and 2000 K, indicating stable structure under extreme temperatures (fig. S43). SAXS and WAXS further confirm that the orientation degree and interlayer spacing (d_002_) remain unchanged under extreme temperatures (figs. S44 and S45). These results reveal that the intrinsic graphitic microstructure of FSGF is stable across the wide temperature range. Common high-performance polymer, metal, or ceramic fibers melt at high temperature above 800 K (*48*–*50*). While the 100-μm-thick FSGFs simultaneously maintain ultrahigh specific tensile strength of 1.53 × 10^3^ MPa g^−1^ cm^3^ and specific bending rigidity of 8.2 × 10^5^ N μm^2^ g^−1^ cm^3^ in an extreme temperature range of 4 to 2000 K (Fig. 3, H to J, and fig. S46). Besides, the conventional metal, polymer, and glass fibers are easily to be corroded in strong acid/alkali environments (*51*, *52*). Nevertheless, the specific tensile strength and specific bending rigidity of 100-μm-thick FSGFs remain 1.58 × 10^3^ MPa g^−1^ cm^3^ and 8.4 × 10^5^ N μm^2^ g^−1^ cm^3^, respectively, in a broad environmental pH from 0 to 14 (Fig. 3, I and K). The structure and mechanical performance of FSGF remains stable even after soaking for 30 days in the acid solution with pH of 0 or the alkali solution with pH of 14 (fig. S47) (*53*–*55*). Three FSGFs were able to support a 300-g weight equivalent to 1000 times of its own weight whether in acidic or alkaline solution (fig. S48). The stable mechanical performance of FSGF in extreme temperature and chemical conditions guarantees their applications in robots, aerospace, and medical devices.

### Robust robotic components in harsh environments

**Fig. 4.**
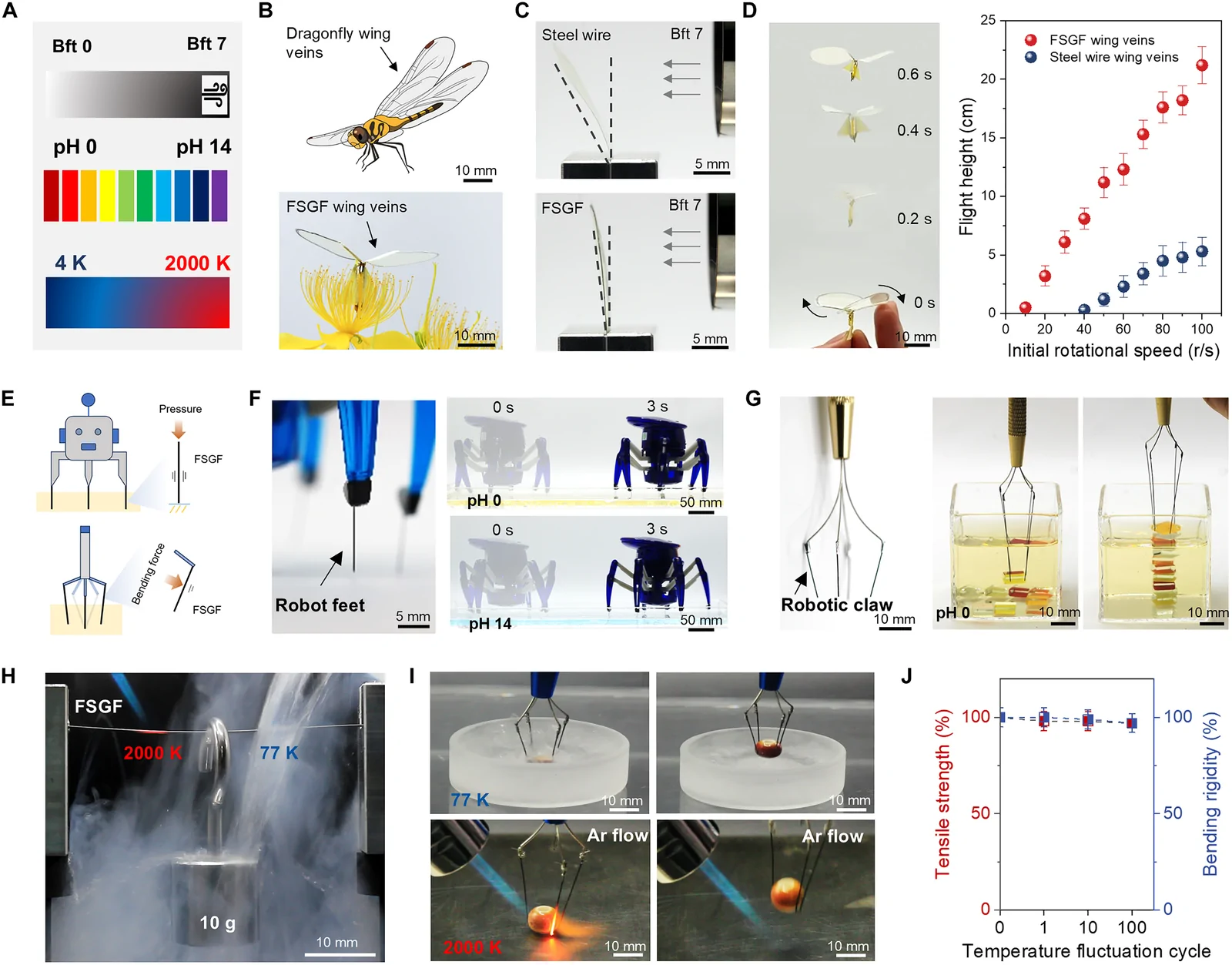
Robust operation of FSGF-integrated microrobots in harsh environments. (**A**) Schematic illustrating the resistance to various extreme conditions of FSGF-integrated microrobots. (**B**) Photographs of a cartoon schematic of a dragonfly (top) and dragonfly-mimic microaerial vehicle with the wing veins of FSGF (bottom). (**C**) Photographs showing the deflection angles of artificial wings with steel wire vein (top) and FSGF vein (bottom) under the high wind of Beaufort scale 7. (**D**) Photograph showing the flight height of a FSGF wing vein–based microaerial vehicle (left) and the flight height comparison of FSGF-integrated and steel wire–integrated microaerial vehicles with varying initial rotational speed (right). (**E**) Schematic illustrations of robots integrated with FSGF feet and claws. (**F**) Photographs showing the FSGF robotic feet (left) and the robust locomotion of FSGF-integrated spider robot in strong acidic and strong alkaline environments (right). (**G**) Photographs demonstrating the FSGF robotic claws (left) and the long-term precise manipulation of small objects using FSGF-integrated robotic gripper in strong acidic environment (right). (**H**) Photograph of 200-μm-thick FSGF bearing a 10-g weight under superimposed extreme conditions of both 2000 K (flame) and 77 K (liquid nitrogen) without deformation failure. (**I**) Typical photographs showing the stable grasp of a bead weighing up to 0.6 g using FSGF-integrated robotic gripper under 77 K (top) and 2000 K (bottom), respectively. (**J**) Tensile strength and bending rigidity retention rates of 100-μm-thick FSGF during cyclic temperature fluctuations between 77 and 2000 K. Illustrations in (B) and (E) were created by the authors using Microsoft PowerPoint.

On the basis of lightweight, ultrahigh strength, ultrahigh rigidity, and thermal and chemical resistance merits, the FSGFs were applied as structural components of microaerial vehicles and robots. These components stay reliable in the extremely harsh environments, such as high wind with Beaufort scale from 0 to 7, strong acid and base with pH from 0 to 14, and extreme cool and hot environments with temperature from 4 to 2000 K, which is unattainable by the traditional fibers (Fig. 4A). First, the strong and rigid graphene fibers were used as wing veins of dragonfly-mimic microaerial vehicles (Fig. 4B and fig. S49). In nature, the dragonflies are capable of a long-distance efficient flight under varying wind conditions in virtue of the lightweight and rigid wing veins (*56*). Inspired by the properties of dragonfly wing veins, the FSGFs with diameter of 100 μm were composited with polyimide films to serve as the wings of micro-rotorcrafts. In the wind resistance tests, the FSGF-based wing demonstrated a better wind resistance than the similar thick steel fiber–based wing. Under a wind speed of Beaufort scale 7, the deflection angle of FSGF-based wing is merely 5°, substantially smaller than 43° of steel fiber–based wing (Fig. 4C). At the same initial rotational speed of 100 r s^−1^, the flight height of FSGF-based micro-rotorcrafts reached as high as 22 cm within 0.6 s, yet that of steel fiber–based micro-rotorcrafts was only 5 cm due to the heavier fiber density (Fig. 4D and movie S3). This simplified wing design serves as a proof of concept to clearly illustrate the material advantages of FSGFs compared to steel fibers.

Second, the extraordinary and stable mechanical properties of graphene fibers enable them to be applied as feet and claws of robots (Fig. 4E). In the case of feet of spider robot, six 200-μm-thick FSGFs were able to bear a 200-g robot weight. With the assistance of FSGF feet, the spider robot continuously walked at a speed of 10 cm s^−1^ in strong acid solution with pH of 0 or strong base solution with pH of 14 (Fig. 4F and movie S4). In addition, four 200-μm-thick FSGFs were used as the claws of robotic gripper. The claws securely grasped a weight as heavy as 5 g from strong acid or alkali solution and then released it in the external environment (fig. S50A). Meanwhile, the FSGF claws persistently and precisely manipulate small-sized objects in strong acidic or alkali solution for more than 5 min. For example, the FSGF claws successfully stacked nine jumbled 1-cm-wide quartz blocks vertically into a straight line in concentrated hydrochloric acid solution with pH of 0 or sodium hydroxide solution with pH of 14 (Fig. 4G, fig. S50B, movie S5, and table S3). Notably, the traditional metal fibers like steel fibers were corroded in liquid metal of gallium within 5 s, failing to serve as robotic claws in corrosive environments (fig. S51) (*57*). While the FSGF claws were capable to persistently grasp and release beads of 0.3 g from liquid metal (movie S6). The stable and efficient actions of FSGF-based robotic components facilitate the applications of robots in various corrosive environments.

Because of the resistance to extremely broad temperature range, the FSGF-based robotic claws realized stable manipulations in extreme cold environments of 77 K and extreme hot environments of up to 2000 K. Amazingly, FSGF with a diameter of 200 μm stably resisted the lateral force of 0.1 N even when two ends were simultaneously exposed to 77 and 2000 K (Fig. 4H). Leveraging the extraordinary cold and thermal resistance, the four FSGF-based robotic claws stably grasped and released a 0.6-g bead in liquid nitrogen of 77 K. Subsequently, the claws grasped and released the weight in methane flame of 2000 K (Fig. 4I and movie S7). After 100 cycles of extreme cold-heat shock, the claws still maintained a stable manipulation. The tensile strength and bending rigidity of the FSGF did not obviously degrade during the 100 cycles of cold-heat shock (Fig. 4J). Furthermore, by leveraging the stable electrical conductivity of FSGF from 4 to 2000 K and in extreme acid/alkali conditions, the FSGF-based claws were used as action-detection integrated components. Under constant current, FSGFs enable in situ identification of conductors and insulators (e.g., graphite versus ceramic) and damage detection of conductive objects by grasping them in extreme environments (77 K, 2000 K, pH 0, and pH 14). The electrical signals remained stable in more than 50 grasping cycles (figs. S52 and S53). The fibers with both high tensile strength of 3.0 GPa and high bending rigidity reaching 1.6 × 10^6^ N μm^2^ open an avenue for the applications in harsh environments, boosting the development of advanced bionic functions, smart robots, medical devices and spacecraft.

## DISCUSSION

In this work, we continuously prepared graphene fibers with the combination of high tensile strength of 3.0 GPa and exceptional bending rigidity of 1.6 × 10^6^ N μm^2^ via multiscale fusion spinning strategy. Continuous fusion and stretching greatly improve sheet alignment and reduce surface wrinkles in single fibers, yielding densified, thick graphene fibers with enhanced tensile strength and bending rigidity, which circumvent the traditional limitations of Griffith’s law. The fibers also demonstrate high thermal conductivity of 1390 W m^−1^ K^−1^ and electrical conductivity of 1.2 × 10^6^ S m^−1^. The fibers are used as wing veins, feet, and claws of microaerial vehicles and robots, showing the resistance to various extreme environments of wind from 0 to 7 Beaufort scale, pH from 0 to 14, and temperature from 4 to 2000 K. These advancements hold great promise for the next-generation bionic aircraft, smart robots, and construction engineering.

## MATERIALS AND METHODS

### Materials

All reagents and chemicals were used as received without further purification, including GO aqueous dispersion (1.0 wt %) and GO/*N*,*N*′-dimethylformamide (DMF) dispersion (1.0 wt %) from Hangzhou Gaoxi Technology Co. Ltd. (average lateral sheet size 39 μm); DMF, ethyl acetate, ethanol, hydrochloric acid, sodium hydroxide, PAN (*M*_w_ = 250,000), titanium dioxide (TiO_2_, ∼20 nm), and aluminum oxide (Al_2_O_3_, ∼30 nm) from Aladdin Chemistry Co. Ltd.; PVA (*M*– = 145,000) and stainless steel wires from Sigma-Aldrich; SA from Macklin; copper and aluminum wires from Alfa Aesar; polyimide films (Kapton HN, 7.5 μm thick) from DuPont; a commercial spider robot (Hexbug Spider) from Spin Master; and a miniature four-finger robotic gripper (10-mm maximum jaw opening) from Gugao Precision Tools Store.

### Fabrication of FSGFs

Continuous graphene fibers were prepared via multiscale fusion spinning. The GO/DMF spinning dope (8 mg g^−1^) was extruded into an ethyl acetate coagulation bath through a multihole spinneret (each hole diameter: 80 μm). The resulting multiple filaments were solidified, dried, and then continuously stretched in a plasticization bath of ethanol/water (3:1 v/v) with a draw ratio of 35%. Subsequently, the highly oriented filament bundle was swollen in a water-ethanol bath and dried, during which the multiple filaments adaptively fused into a single thick fiber. The fused fiber was further stretched (draw ratio 30%) and dried to obtain FSGOF. After sequential drying at 60°C and 200°C, the fibers were thermally reduced at 1000°C and 2800°C to produce FSGFs. For comparison, DSGFs were prepared using a single-hole spinneret with the same spinning dope. The resulting filament was stretched in an ethanol-based plasticization bath with a draw ratio below 10% due to limited solvent intercalation, followed by the same drying and thermal reduction steps as for FSGFs.

### Fabrication of strong and rigid composite fibers via multiscale fusion spinning

The multiscale fusion spinning strategy was extended to prepare composite fibers. For PAN/GO composite fibers, PAN/DMF and GO/DMF solutions (10 mg g^−1^ each, 1:1 mass ratio) were mixed to form a composite spinning dope. After degassing, the dope was extruded through a multihole spinneret into an ethyl acetate coagulation bath. The resulting multiple filaments were stretched, swollen in DMF, dried to fuse into a single fiber, and then further stretched and dried to obtain fusion-spun PAN/GO composite fibers. PVA/GO and SA/GO composite fibers were prepared following the same procedure.

### Fabrication of FSGF-integrated robots

FSGFs were used to construct the following robotic components. (i) Wing veins of microaerial vehicles: One hundred–micrometer-thick FSGOFs were partially swollen in water/ethanol (1:5 w/w), bent into a dragonfly-wing shape, fixed with concentrated GO, carbonized at 2800°C under argon, and adhered to polyimide films using ultraviolet (UV)–curable adhesive. Steel wires (0.2 mm diameter) were used as a control. (ii) Feet of spider robot: Six 200-μm-thick FSGF segments (2 cm each) were attached to the legs of a commercial spider robot with UV-curable adhesive. (iii) Claws of robotic gripper: Four 200-μm-thick FSGFs were fixed onto the connections of a miniature four-finger gripper. (iv) Action-detection integrated gripper: Copper wires were attached to the FSGF claws using conductive silver paste, with two opposing claws serving as a pair of electrodes. A four-wire method was used to measure electrical signals during grasping.

### Characterizations

The thickness of GO sheets was characterized by atomic force microscopy (Bruker Multimode 8). The microscale morphologies of fibers were observed by SEM (Hitachi S4800) at 3 kV, and three-dimensional microstructures were reconstructed using FIB-SEM (Helios G3 UC system) with 10-nm slicing intervals. EDS was performed at 15 kV for elemental analysis. Fiber density was evaluated by the sink-float method using tetrabromoethane, carbon tetrachloride, and hexamethylene (*36*, *39*). SAXS and WAXS measurements were performed at the Shanghai Synchrotron Radiation Facility on beamlines BL10U1 (SAXS: sample-to-detector distance, 4500 mm) and BL16B1 (SAXS: 2000 mm; WAXS: 72 mm) to characterize graphene sheet orientation (*26*). Crystalline structures were examined by spherical aberration-corrected TEM (FEI Titan G2 60-300) at 80 kV. Chemical compositions were determined by x-ray photoelectron spectroscopy (Escalab 250Xi) under ultrahigh vacuum using monochromatic Al Kα radiation (*16*). In situ SEM and in situ Raman spectroscopy (Renishaw, Via-Reflex, 532-nm excitation) were conducted during tensile or bending deformation to monitor structural evolution and strain-induced spectral shifts. Electrical conductivities were measured by a standard four-probe method (Keithley 2611B) at room temperature. To trace the fusion process, labeled GO filaments (TiO_2_ and Al_2_O_3_) were brought into contact under the same swelling/drying conditions; samples at different fusion stages were freeze-dried and examined by SEM/EDS. Fusion of a 50-filament bundle was also traced by SEM.

### Mechanical performance tests under room temperature

Tensile strength was measured using a universal testing machine (Instron Legend 2344) at 1 mm min^−1^. At least 10 samples were tested per fiber type. Bending rigidity (*B*) was evaluated by two methods.

Three-point bending test: A fiber was placed across a 10-mm span and loaded at the midspan at 1 mm min^−1^. *B* was calculated as follows (*41*, *42*)

B=FL3192δ (1)

where *F* is fracture load, δ is midspan deflection, and *L* is support span.

Modulus-diameter method: *B* was calculated as follows (*41*)

$$B=\mathrm{EI},\;\text{with}\;I=\frac{\pi D^{4}}{64}$$ (2)

where *E* is Young’s modulus (approximated as bending modulus) and *D* is fiber diameter. For fibers with irregular cross section (e.g., DSGFs), only the three-point bending method was used.

### Mechanical performance tests under extreme temperatures

Tensile tests at 4 K were performed using a testing machine equipped with a liquid helium closed-circulation system (SANS CMT5000). Tensile tests at 2000 K were conducted using a universal testing machine (Instron Legend 2344) under argon atmosphere, during which the fibers were heated by a 2000-K flame gun while being stretched. Stress-strain curves were recorded, and mechanical properties were derived accordingly.

### The homogeneity evaluation of mechanical performance

To evaluate mechanical homogeneity, tensile strengths were compared for FSGFs and DSGFs with diameters from 5 to 100 μm (gauge length, 5 mm) and for fibers with gauge lengths from 5 to 100 mm (diameter 100 μm). All tests used a tensile rate of 1 mm min^−1^. The coefficient of variation of strength was calculated as the ratio of SD to the mean value.

### Thermal conductivity measurements

Thermal conductivity was measured using a modified steady-state electrothermal method (*6*). Under high vacuum (∼10^−4^ Pa), a direct current was applied to the fiber, and the axial temperature distribution was monitored by an infrared camera (FLIR T630sc). The thermal conductivity κ was calculated as follows

$$\kappa =\frac{\mathrm{UIL}}{4A_{c}\left(T_{0}-T_{a}\right)}$$ (3)

where *U* is voltage drop, *I* is the current, *L* is half the distance between the two central contacts, $A_{c}$ is the fiber cross-sectional area, $T_{0}$ is the midpoint temperature, and $T_{a}$ is the edge temperature.

### Extreme environment tolerance of FSGF

The tolerance of FSGF to high wind was evaluated by comparing FSGF-based and steel wire–based artificial dragonfly wings under varying wind speeds (Beaufort scale 0 to 7), recording the bending angle. Acid/alkali tolerance was tested by immersing 100-μm-thick FSGF in 1 mM HCl or NaOH for up to 100 days, followed by structural and mechanical characterization. Cyclic temperature fluctuation tolerance was assessed by alternately immersing fibers in liquid nitrogen (77 K, 1 min) and heating in argon at 2000 K (1 min) for multiple cycles, with mechanical properties measured after each cycle.
