# Supplementary material for: Bioinspired strong and rigid fusion-spun graphene fibers for harsh robotics
Source: Sci Adv. 2026 Sep 4;12(36):eaeg6444. doi: 10.1126/sciadv.aeg6444 (PMC13544223; doi:10.1126/sciadv.aeg6444)
Supplement: Supplementary file 1 [file sciadv.aeg6444_sm.pdf]

Science Advances
AAAS

Supplementary Materials for

Bioinspired strong and rigid fusion-spun graphene fibers for harsh robotics

Yiwei Zhang *et al.*

Corresponding author: Dan Chang, dchang68@zju.edu.cn; Zhen Xu, zhenxu@zju.edu.cn;
Chao Gao, chaogao@zju.edu.cn

Sci. Adv. **12**, eaeg6444 (2026)
DOI: 10.1126/sciadv.aeg6444

The PDF file includes:

Supplementary Text
Figs. S1 to S53
Tables S1 to S3
Legends for movies S1 to S7

Other Supplementary Material for this manuscript includes the following:

Movies S1 to S7

Supplementary Text

Calculation of the sheet interaction energy at filament interface during fusion

The interaction energy between GO sheets at the filament interface during fusion of multiple filaments was calculated. Based on the DLVO theory, the condensed state is dominated by the attractive van der Waals (vdW) force and repulsive electrostatic interaction. The vdW force between two thin sheets is formulated as follows:

$$WvdW(d) = -\frac{AH}{12\pi}\left(\frac{1}{d^2} + \frac{1}{(d+2t)^2} - \frac{2}{(d+t)^2}\right) \quad (1)$$

where H, A, d, and t are the Hamaker constant, the contact area, the interlayer spacing, and the thickness of the sheets, respectively. The H between two interacting semi-infinite GO sheets across a medium can be estimated as follows:

$$H = \frac{3}{4}kT\left(\frac{\varepsilon_1 - \varepsilon_2}{\varepsilon_1 + \varepsilon_2}\right)^2 + \frac{3hv_e}{8\sqrt{2}}\frac{(n_1^2 - n_2^2)^2}{(n_1^2 + n_2^2)\left(2\sqrt{n_1^2 + n_2^2}\right)} \quad (2)$$

where k is the Boltzmann constant, T is the absolute temperature, ε and n are the dielectric constant and refractive index of medium and sheets, h is the Planck constant, and v_e ($\approx 3 \times 10^{15}$ s^{-1}) is the main absorption frequency in UV region. The dielectric constant of GO sheets and water are 3 and 78.5, respectively. The refractive index of GO sheets and water are 1.75 and 1.33, respectively. The thickness of GO is about 1.1 nm. The value of H is 44.3×10^{-21} J. Therefore, the vdW potential energy between GO sheets versus d can be calculated as:

$$EvdW(d) = -\frac{H}{12\pi}\left(\frac{1}{d^2} + \frac{1}{(d+2t)^2} - \frac{2}{(d+t)^2}\right) \quad (3)$$

The electrostatic double layer (EL) potential between two identical interfaces is calculated based on the linearized Poisson−Boltzmann approximation for two planar surfaces:

$$P_{EL} = \frac{2\lambda\delta^2}{\varepsilon\varepsilon_0}\exp\left(-\frac{d}{\lambda}\right) \quad (4)$$

where δ is the surface charge density, ε_0 is the permittivity of vacuum, ε is the relative dielectric constant of solvent, and λ is the Debye length, which is given by

$$\lambda = \sqrt{\frac{\varepsilon\varepsilon_0 k_b T}{2N_A e^2 I}} \quad (5)$$

where N_A is the Avogadro number, e is the elementary charge, and I is the ionic strength of solution.

It is assumed that the ionic strength at the fusing interface doesn't change with increase of

concentration of gel GO fiber. The pH of pure GO aqueous dispersions is about 5 and the concentration of ionized groups is about 0.01 mol L^{-1}. The groups are ionized from -COOH and -OH, thereby the valency of ionized groups is +1 or -1. Therefore, λ is calculated as about 4.3 nm. δ of GO sheets dispersed in water was measured by pH titration and is about 0.02 C m^{-2}. Consequently, the electrostatic potential energy between GO sheets can be calculated as:

$$E_{EL} = \frac{2 \times 4.3nm(0.02Cm^{-2})^3}{\varepsilon\varepsilon_0} \exp\left(-\frac{d}{4.3nm}\right) \tag{6}$$

The DLVO interactions between two GO sheets can be calculated as:

$$E_{DLVO} = E_{vdW} + E_{EL} \tag{7}$$

As the interlayer spacing reaches the critical concentration, the stacking of GO sheets occurs and following condition is satisfied:

$$\frac{\partial}{\partial d} E_{DLVO} = 0 \tag{8}$$

Calculation of the theoretical diameter of FSGF

The theoretical diameter of FSGF was calculated according to the filament number in the multiscale fusion spinning process. Assuming that there are no voids between the fused fibers, the cross-sectional area of the fused fiber is equal to the sum of the cross-sectional area of individual filaments. Therefore, the theoretical diameter of FSGF satisfies the following equation:

$$\pi\left(\frac{D}{2}\right)^2 = n\pi\left(\frac{d}{2}\right)^2 \tag{9}$$

where D and d denote the diameters of FSGF and individual filaments, respectively. n is the number of multiple filaments in fusion. Accordingly, the theoretical diameter D of FSGF is given by:

$$D = \sqrt{n}d \tag{10}$$

Calculation of circularity degree

The degree of circularity of fibers was defined based on the deviations of circumference (C) and cross-sectional area (S) with the equivalent circle. The circumference and cross-sectional area of fibers with radius of R can be calculated as $C = 2\pi R$ and $S = \pi R^2$, respectively. The cross-sectional shape deviation from a standard circle causes an increase in C and a decrease in S. The circularity φ of fibers is defined as:

$$\varphi = \frac{2\sqrt{S}}{C} \tag{11}$$

where φ of a standard circle is $\frac{1}{\sqrt{\pi}}$.

Calculation of orientation degree in SAXS and WAXS

The orientation of fibers was characterized by WAXS and SAXS, respectively. The scattering vector q is defined as $q = 4\pi sin\ \theta/\lambda$, where θ is the scattering angle. The sheet orientation degree of fibers was obtained by converting the orientation distribution into a Hermans ordering parameter h, which is defined as:

$$h = \langle \frac{3}{2}cos^2\varphi - \frac{1}{2} \rangle \tag{12}$$

where φ is the azimuthal angle. By extending the average, the equation is calculated as:

$$h = \int_0^{\pi} I(\varphi)\left(\frac{3}{2}cos^2\varphi - \frac{1}{2}\right) sin\varphi d\varphi \tag{13}$$

which is normalized according to:

$$\int_0^{\pi} I(\varphi) sin\varphi d\varphi = 1 \tag{14}$$

where $I(\varphi)$ is the density distribution along a constant q.

The interlayer spacing is calculated by $d = 2\pi/q^*$, where q^* denotes the wave vector of Iq^2 peak. The density I is obtained by integrating the 2D patterns.

Calculation of the coefficient of variation (CV) of fiber diameter

To quantify the morphological uniformity of fibers along the axis, the CV values of fiber diameter were calculated. Specifically, the diameter of a 5 cm-long fiber was measured at different axial positions with an interval of 500 μm based on the SEM image. The CV of diameter is calculated as the ratio of the standard deviation of diameters to the average diameter, which is given by:

$$CV_d = \frac{SD_d}{\overline{D}} \tag{15}$$

where SD_d is the standard deviation of diameters. $\overline{D}$ is the average diameter.

Calculation of the average porosity

Based on the FIB-SEM reconstructions, the average porosity of fibers (P) was calculated as:

$$P = \frac{\sum V_i}{V} \tag{16}$$

where V_i is the volume of a single pore, V is the total volume of the FIB reconstructed sample.

The number and size of micro-voids within the fibers were analyzed by the three-dimensional reconstruction of FIB-SEM. The average pore volume $\bar{V}$ was calculated as follows:

$$\bar{V} = \sum_i \left(\frac{V_i}{\sum V_i} \cdot V_i \right) \tag{17}$$

where V_i is the volume of a single pore.

Calculation of mechanical requirements for FSGF in robotic applications

(1) Micro-aerial vehicle wing vein (anti-wind load)

The FSGF wing vein is simplified as a cantilever beam. Under a distributed wind load, the equivalent tip force F is derived from the measured deflection angle θ at the free end. The deflection formula for a cantilever beam with a tip load gives:

$$\theta = \frac{FL^2}{2EI} \tag{18}$$

where L is the beam length, E is the elastic modulus, and I is the area moment of inertia. The required bending rigidity EI is then obtained from the target deflection angle.

The maximum bending stress is obtained from the root bending moment and the section modulus:

$$\sigma = \frac{M}{W} = \frac{FL}{W}, \qquad \text{with } W = \frac{\pi d^3}{32} \tag{19}$$

where M is the bending moment at the root, W is the section modulus, and d is the fiber diameter.

(2) Spider robot foot buckling resistance

The fiber is clamped at the upper end and pinned at the lower end (effective length factor K=0.7). Using Euler's buckling formula, the required bending rigidity to avoid buckling is:

$$P_{cr} = \frac{\pi^2 EI}{(KL)^2} \tag{20}$$

where E is the elastic modulus, I is the moment of inertia, and L is the foot length.

(3) Robotic gripper claw (bending during grasping)

The gripper claw is analogous to the cantilever beam model in part (1). When grasping an object, a tip force F is applied at the free end. The deflection angle θ is limited by the gripping precision.

Calculation of Weibull modulus for FSGF with different diameters

To provide statistical evidence of defect accumulation control with increasing fiber diameter, Weibull analysis was performed on FSGF and DSGF of different diameters. Tensile tests were

carried out on FSGF and DSGF with four different diameters using at least 30 specimens per diameter. The cumulative failure probability was fitted with the two-parameter Weibull distribution:

$$P_f = 1 - exp\left[-\left(\frac{\sigma}{\sigma_0}\right)^m\right] \quad (21)$$

where P_f is the cumulative failure probability, σ is the tensile strength, σ_0 is the scale parameter (characteristic strength), and m is the Weibull modulus. The Weibull modulus m quantifies the dispersion of strength: a higher m indicates lower variability and more homogeneous defect distribution. The modulus was determined by linear regression of $ln[-ln(1-P_f)]$ versus $ln\sigma$.

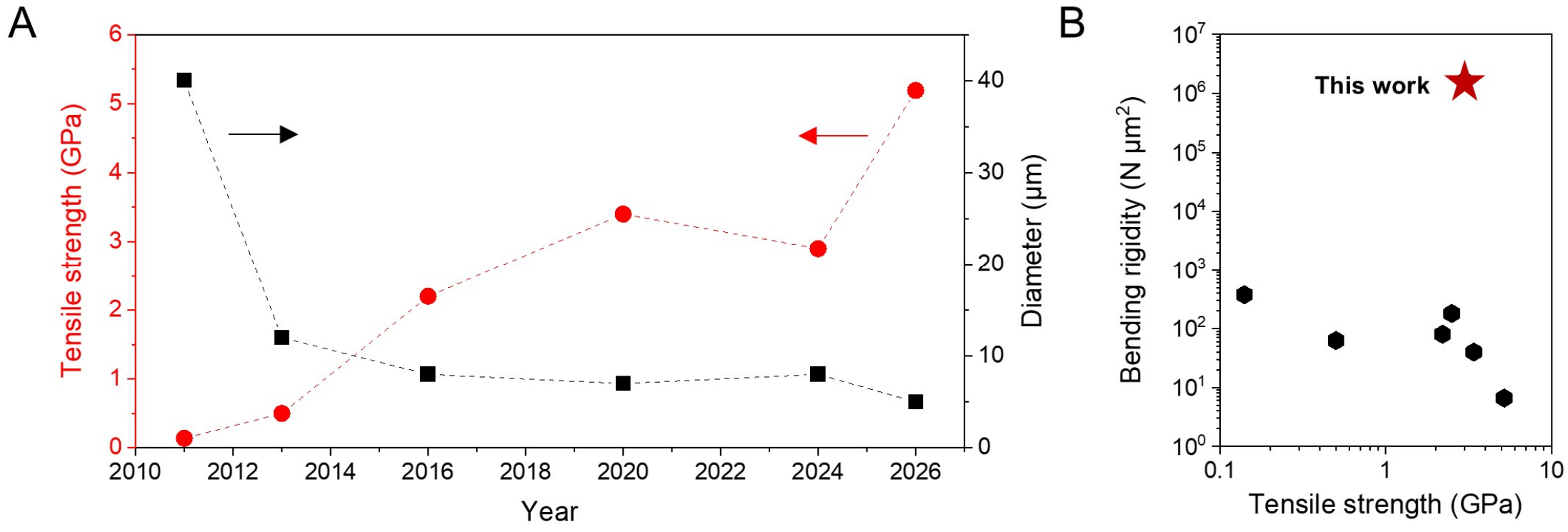

Fig. S1. Trends of fiber properties in previous works on graphene fibers. (**A**) Trends of tensile strength and fiber diameter. (**B**) Trends of tensile strength and bending rigidity.

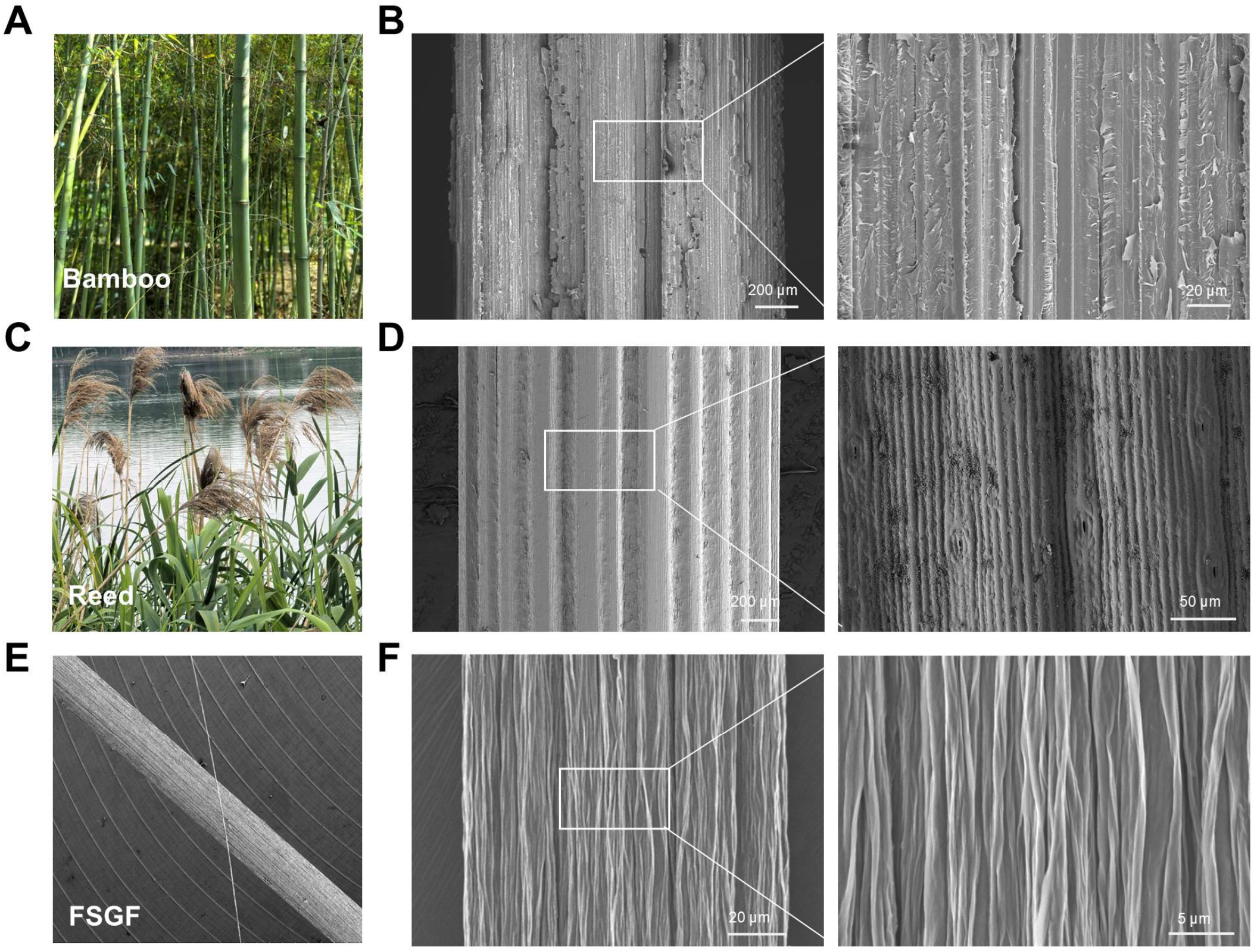

Fig. S2. The structure of plant stems and FSGF. (**A**) Digital photograph of bamboos. (**B**) SEM images showing the lateral view (left) and magnified region (right) of a bamboo pole split lengthwise. (**C**) Digital photograph of reeds. (**D**) SEM images of the lateral surface (left) and magnified region (right) of reeds. (**E**) SEM image of 100 μm-thick FSGF and a single filament. (**F**) SEM images of the lateral surface (left) and magnified region (right) of FSGF. Photos in (A) and (C) were taken by the authors at Zhejiang University Zijingang Campus.

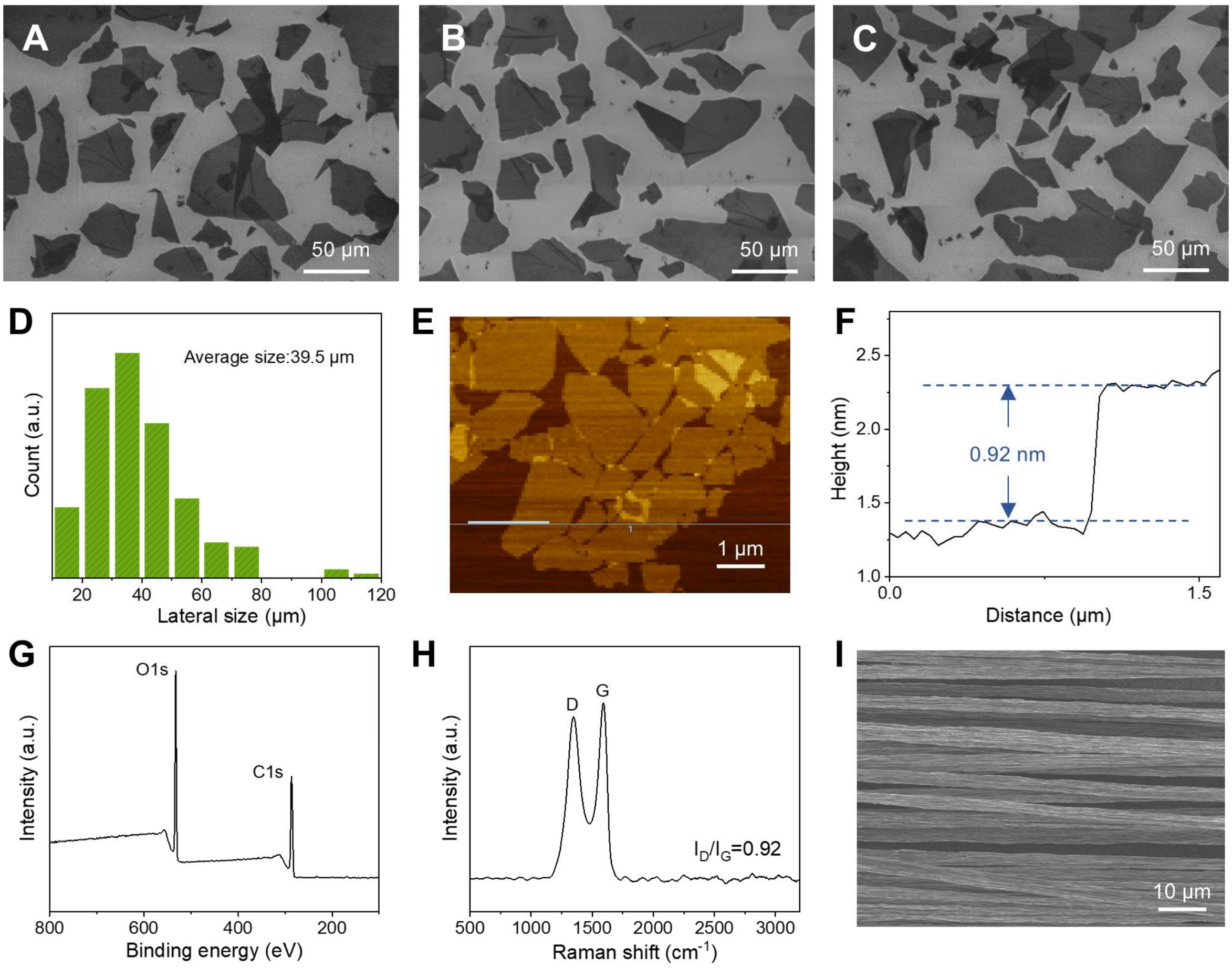

Fig. S3. Characterizations of GO sheets and GO filaments. (**A** to **C**) SEM images of GO sheets. (**D**) Statistical distribution of lateral dimensions of GO sheets. (**E** and **F**) Atomic force microscope (AFM) image (E) demonstrating ~0.92 nm thickness of monolayer GO sheet (F). (**G** and **H**) X-ray photoelectron spectroscopy (XPS) spectrum (G) and Raman spectrum (H) of the GO sheets. (**I**) Unfused GO filaments spun through a 100-hole spinneret.

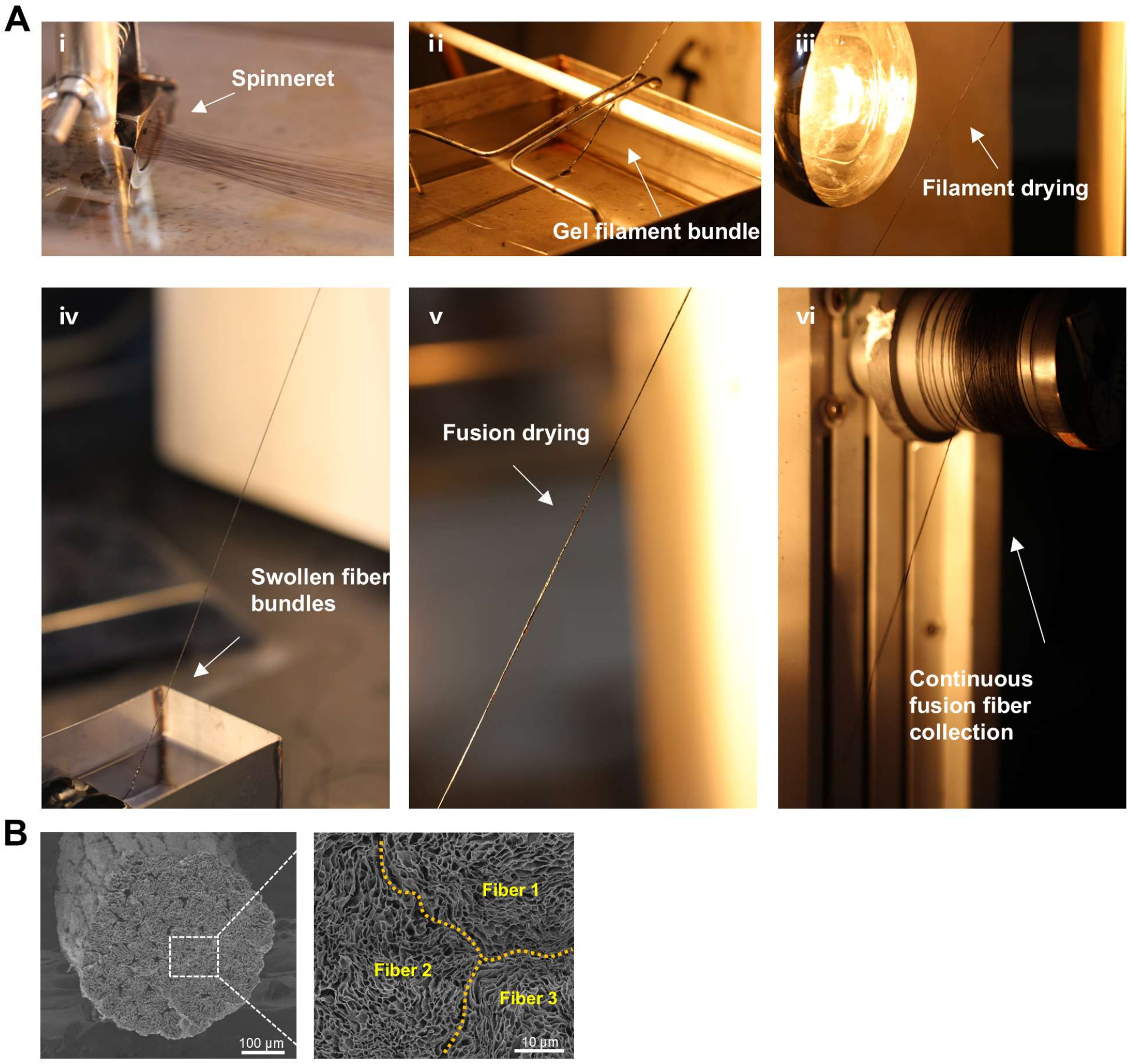

Fig. S4. Multiscale fusion spinning process. (**A**) Photographs showing the critical stages in multiscale fusion spinning process, including: extruding the bundle of 100 filaments through a 100-hole spinneret (i); pulling out the gel filament bundle from the coagulation bath (ii); The fiber bundle is dried under infrared lamp irradiation (iii). The fiber bundles swell in a swelling bath and are raised together (iv). The swollen fiber bundles are adaptively deformed and fused during drying (v). The dried fusion fibers are continuously collected on a large diameter wire takeaway (vi). (**B**) SEM images showing the cross-sectional views of FSGOF before fusion (for the SEM image of the dry state after fusion, see Fig. 1G in the main text).

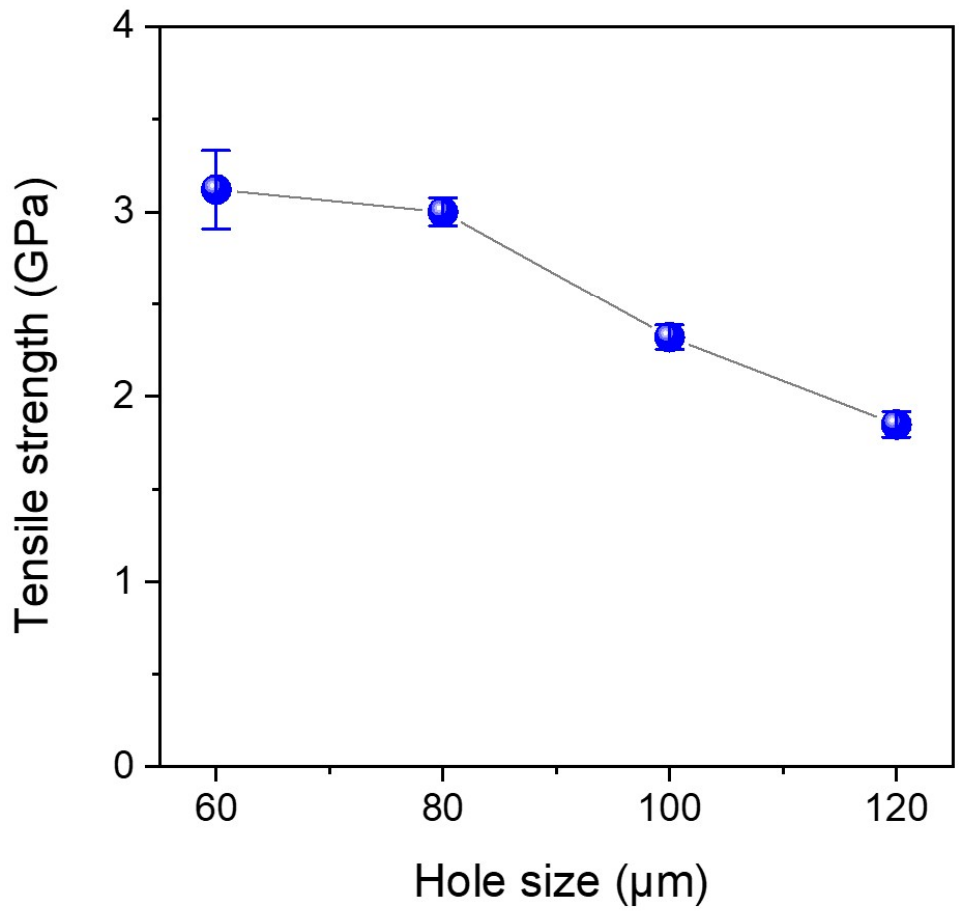

Fig S5. The tensile strength of FSGF prepared by different individual hole size in fusion spinning process.

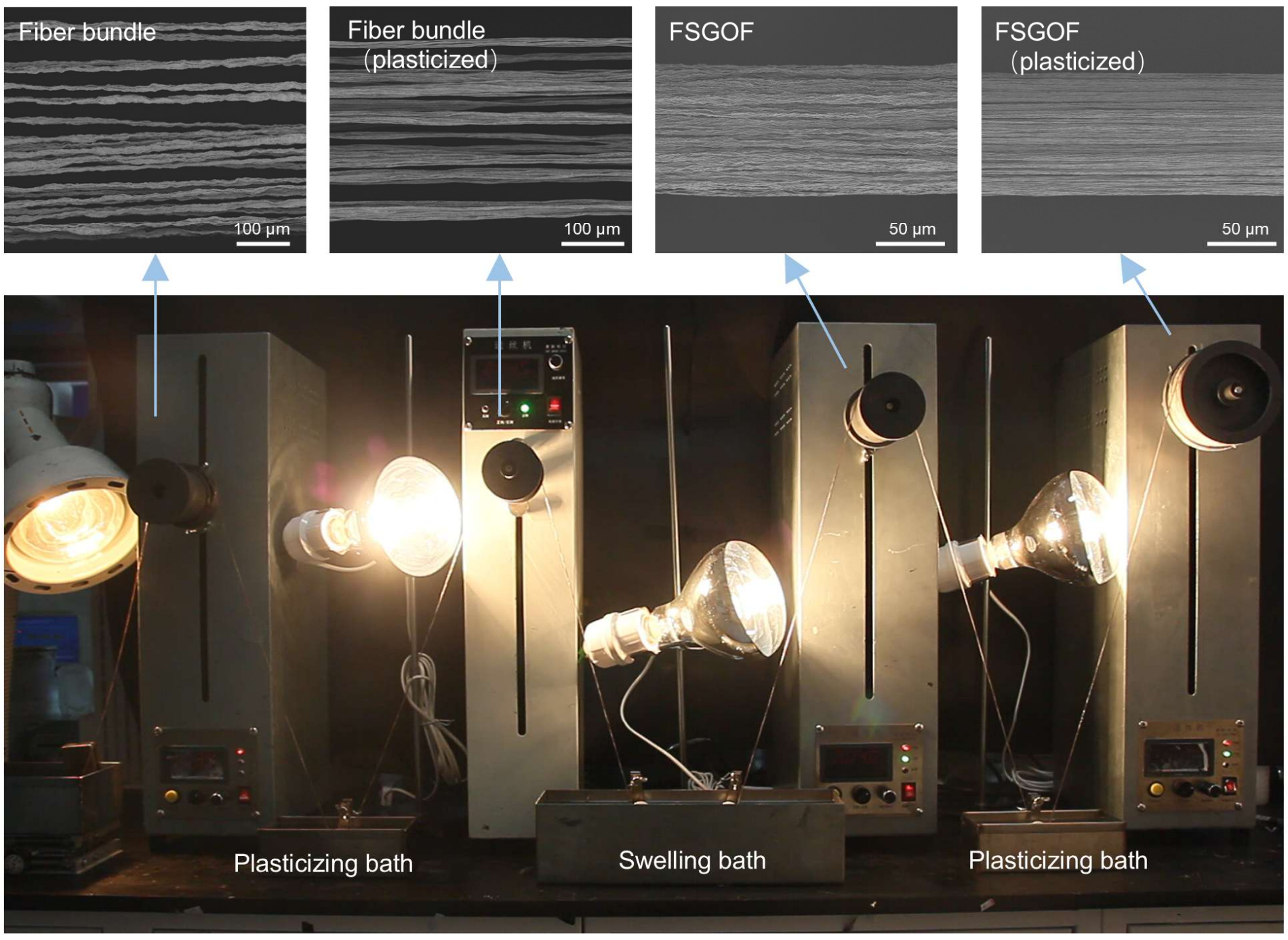

Fig. S6. Continuous preparation process of fibers and SEM images (Top) of fibers at corresponding stages.

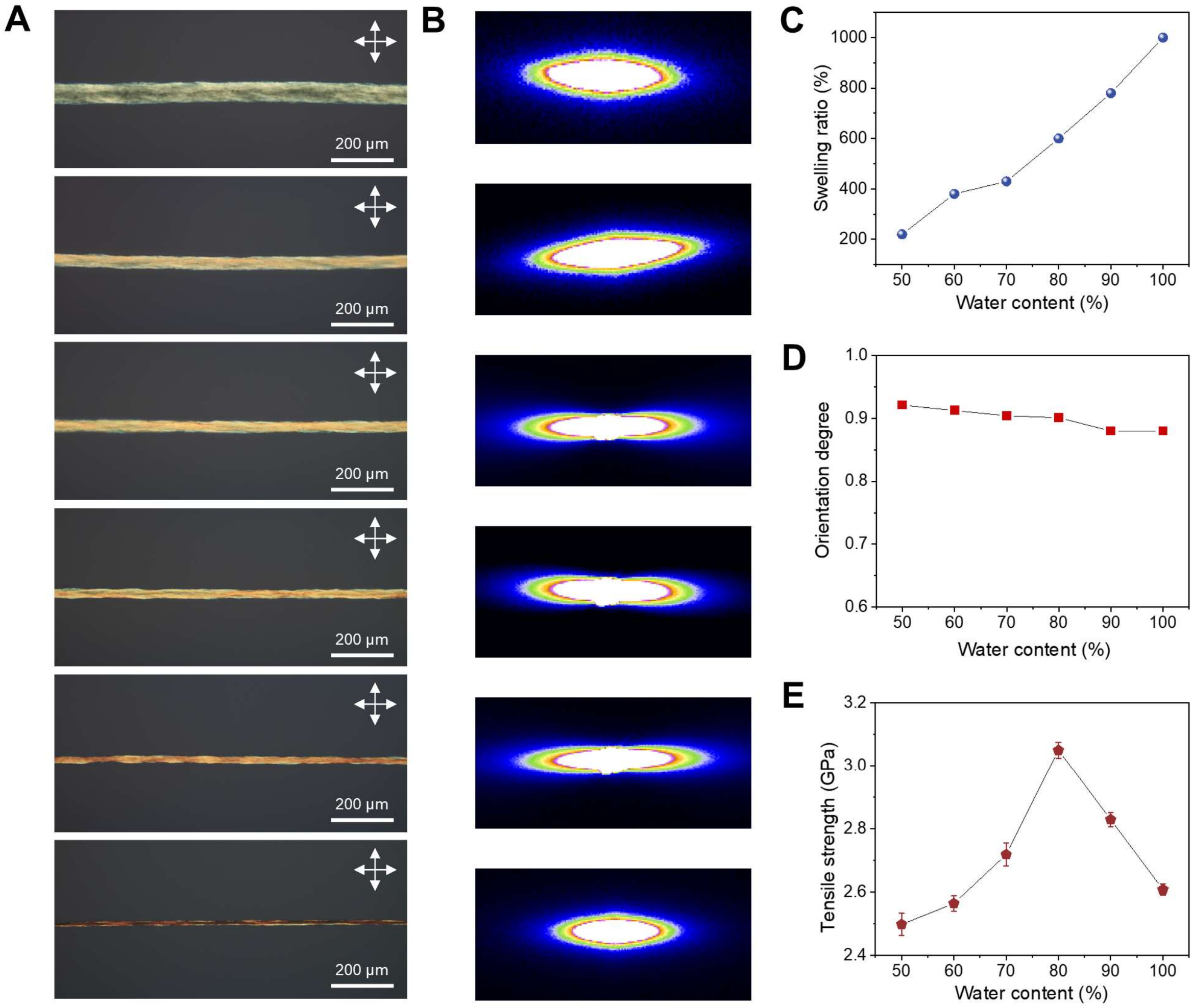

Fig. S7. Influence of swelling ratio to the filament structure and mechanical performance of the resultant FSGF. (**A**) Polarized optical micrographs of a single swollen GO filament in the swelling bath with different volume ratios of water and ethanol, comprising 10:0, 9:1, 8:2, 7:3, 6:4 and 5:5 respectively (from top to bottom). (**B**) SAXS patterns of the swollen GO filaments corresponding to (A). (**C**) Swelling ratio of GO filaments versus the water content in water-ethanol swelling bath. With the increase of water content in the solvent, the swelling degree rises from 220% to 1000%. (**D**) The orientation of gel fibers decreased slightly with the increase of swelling degree. (**E**) FSGF strength with different fiber swelling degrees.

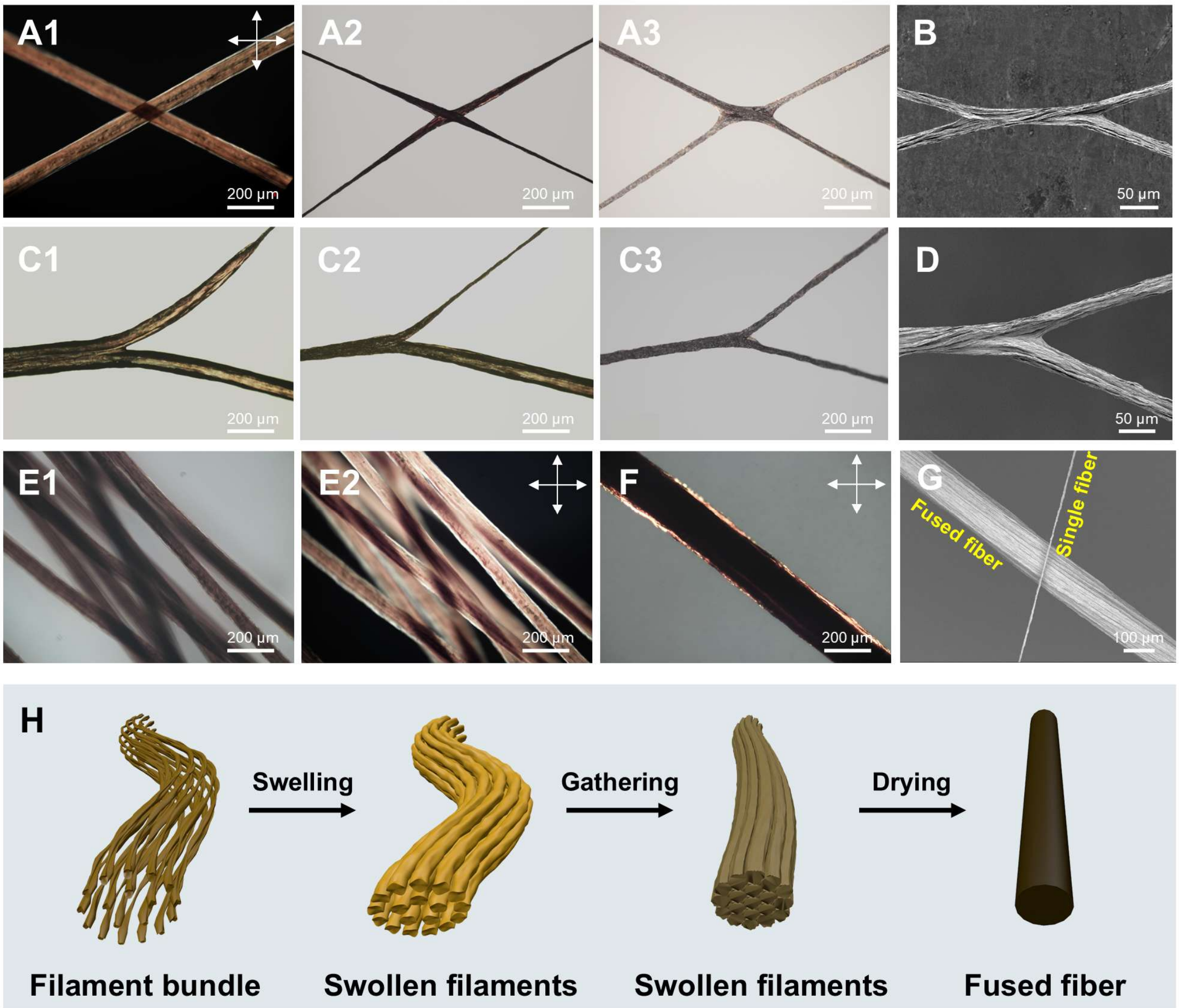

Fig. S8. Fusion process of swollen GO filaments without the limitation of filament number. (**A1** to **A3**) Sequential polarized optical and optical micrographs showing the point fusion process of two swollen GO filaments. (**B**) SEM image of the fused point of two GO filaments corresponding to (A3). **(C1 to C3)** Tracking of line fusion processing of swelling GO fiber by optical micrographs. (**D**) Corresponding SEM image of fused joint of two single fiber in (C). (**E1** to **E2**) optical micrographs and polarized optical micrographs images of swelling fiber bundles before fusion. (**F**) optical micrographs image of fused graphene oxide fiber. (**G**) Comparison of wet spinning single graphene oxide fiber and fused graphene oxide fiber (the same SEM image as Fig. 1E in the main text). (**H**) Schematic diagram of fusion of single graphene oxide fiber to produce FSGOF.

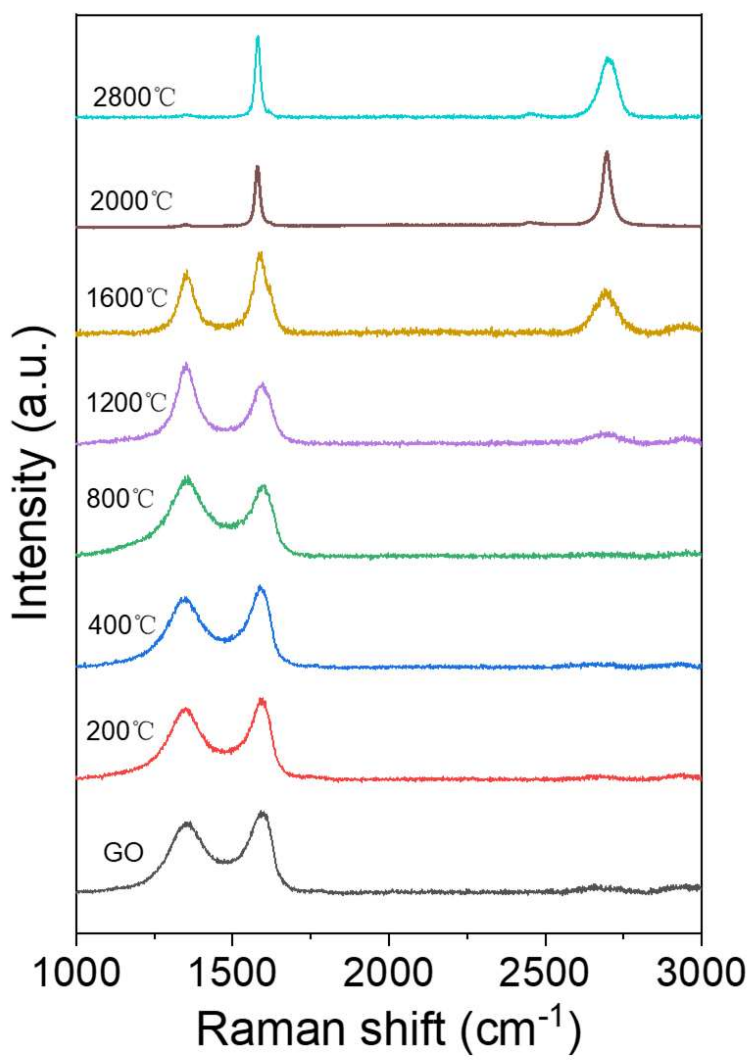

Fig. S9. Raman spectrum of FSGOF and the fibers annealed at different temperatures.

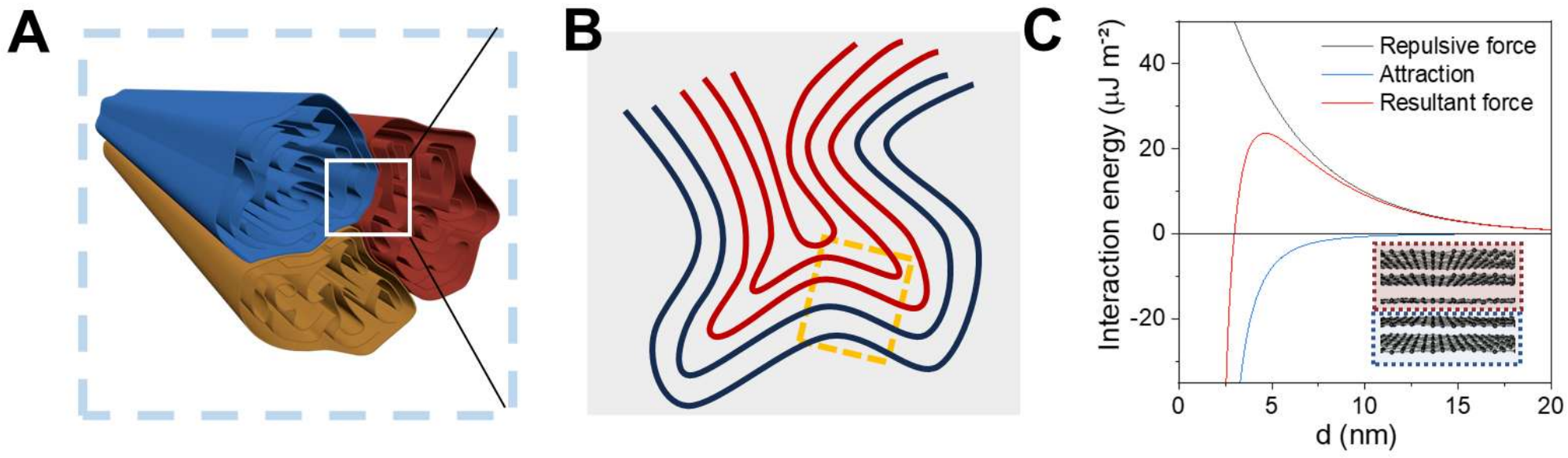

Fig. S10. Interfacial interactions among multiple GO filaments after multiscale fusion spinning. (**A**) Schematic illustrating the adaptive deformation at the contacting interfaces of multiple GO filaments in FSGOF. (**B**) Schematic diagram of the sheet arrangement structure of adjacent GO filaments in FSGOF. (**C**) Interaction energy between the interfacial GO sheets versus the sheet distance. Inset shows the diagram of interfacial GO sheets of two adjacent GO filaments during fusion.

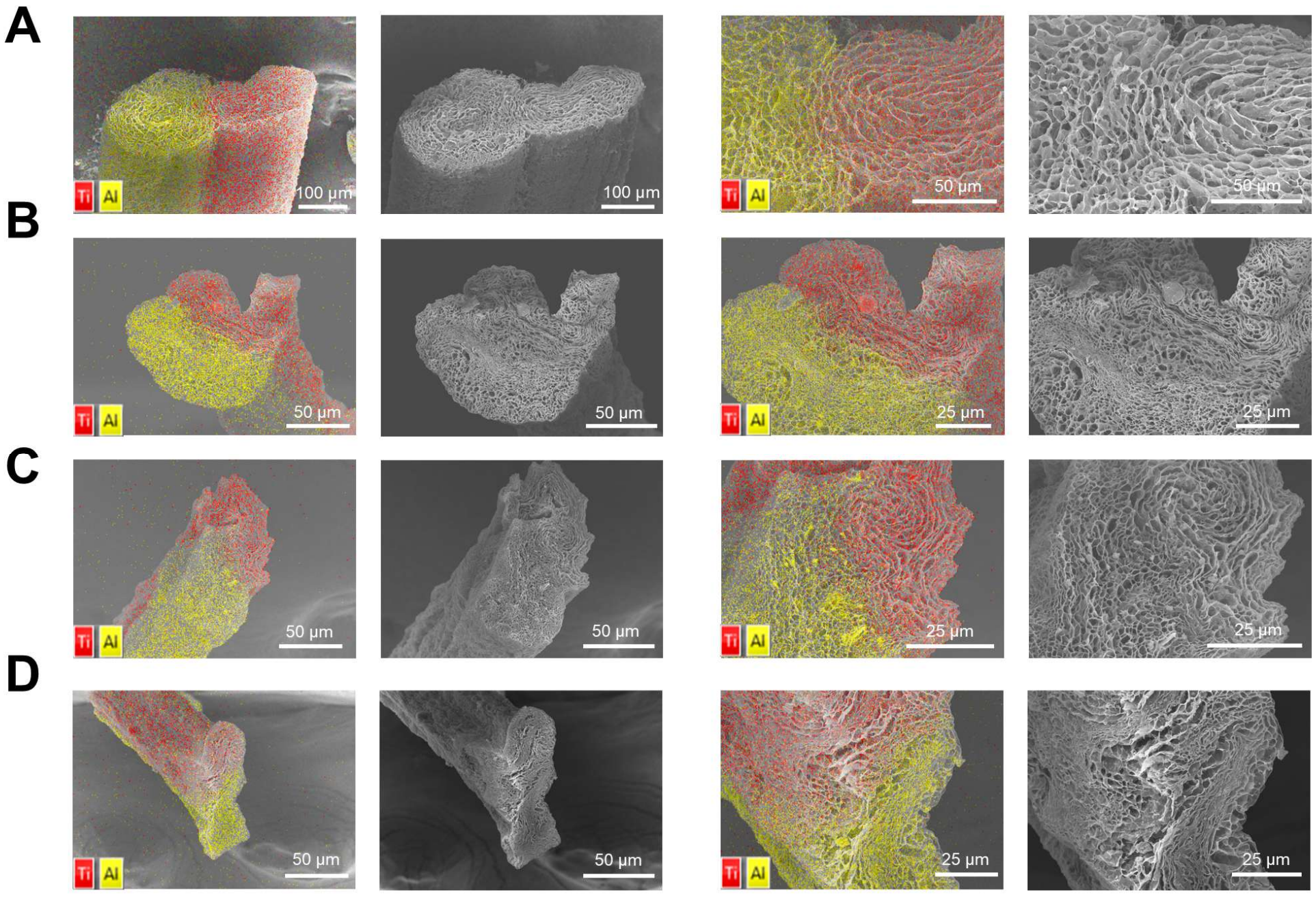

Fig. S11. Fusion process of two element-labeled fibers (Ti and Al elements). (**A**) Initial contact: two adjacent fibers with no significant deformation at the interface. (**B** and **C**) Partial fusion: complementary deformation appears at the interface, indicating the onset of fiber fusion. (**D**) Complete fusion: interlocking morphology appears at the fusion boundary of the two fibers.

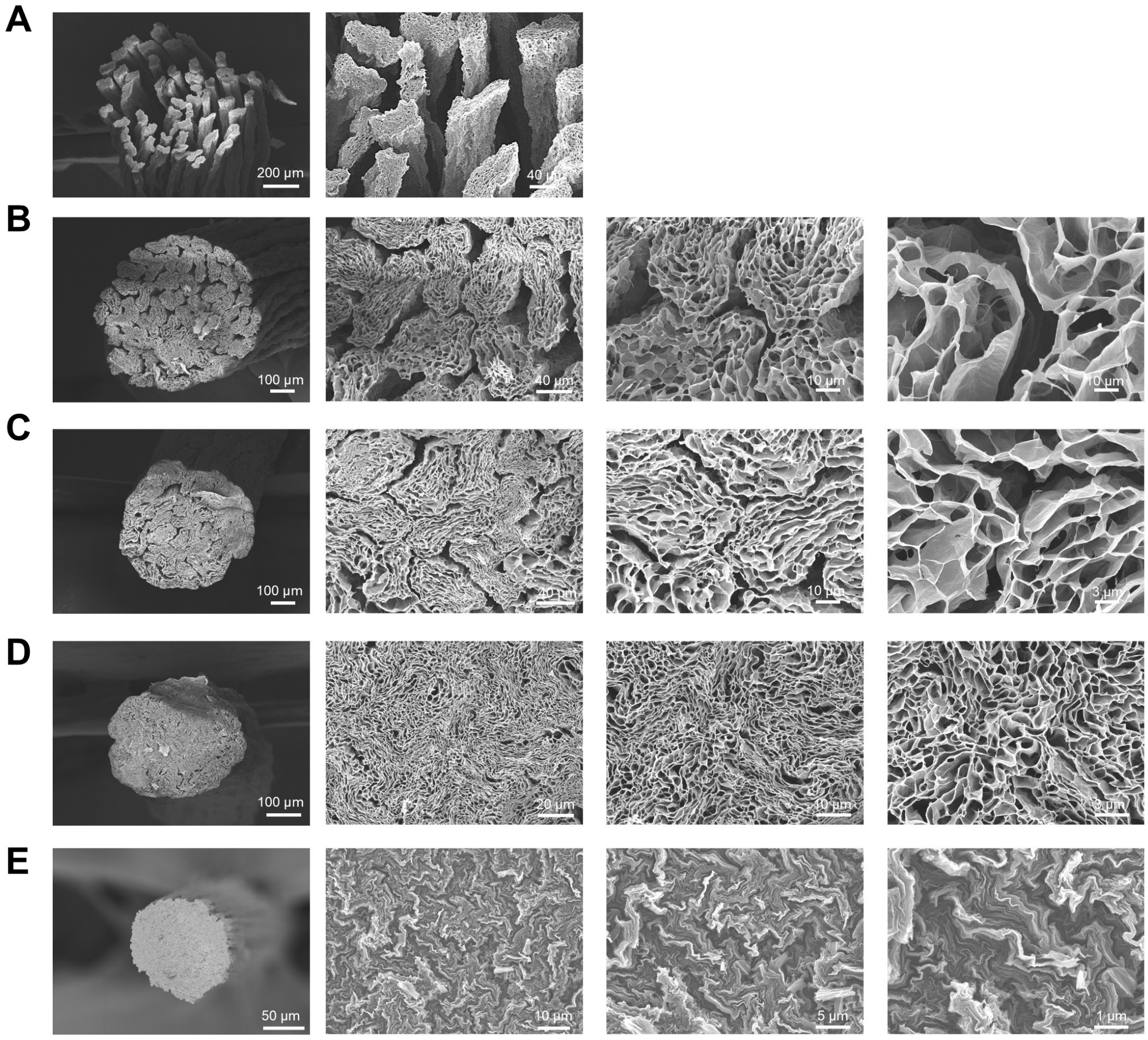

Fig. S12. Fusion process of a filament bundle. (**A**) Before fusion: individual filaments are separate with clear gaps. (**B** to **D**) During fusion: solvent evaporation induces adaptive deformation; cross-sections become polygonal, gaps close, and adjacent filaments conform to each other's shape. (**E**) After complete fusion: filaments merge into a single uniform, nearly circular fiber with no residual gaps or interfaces.

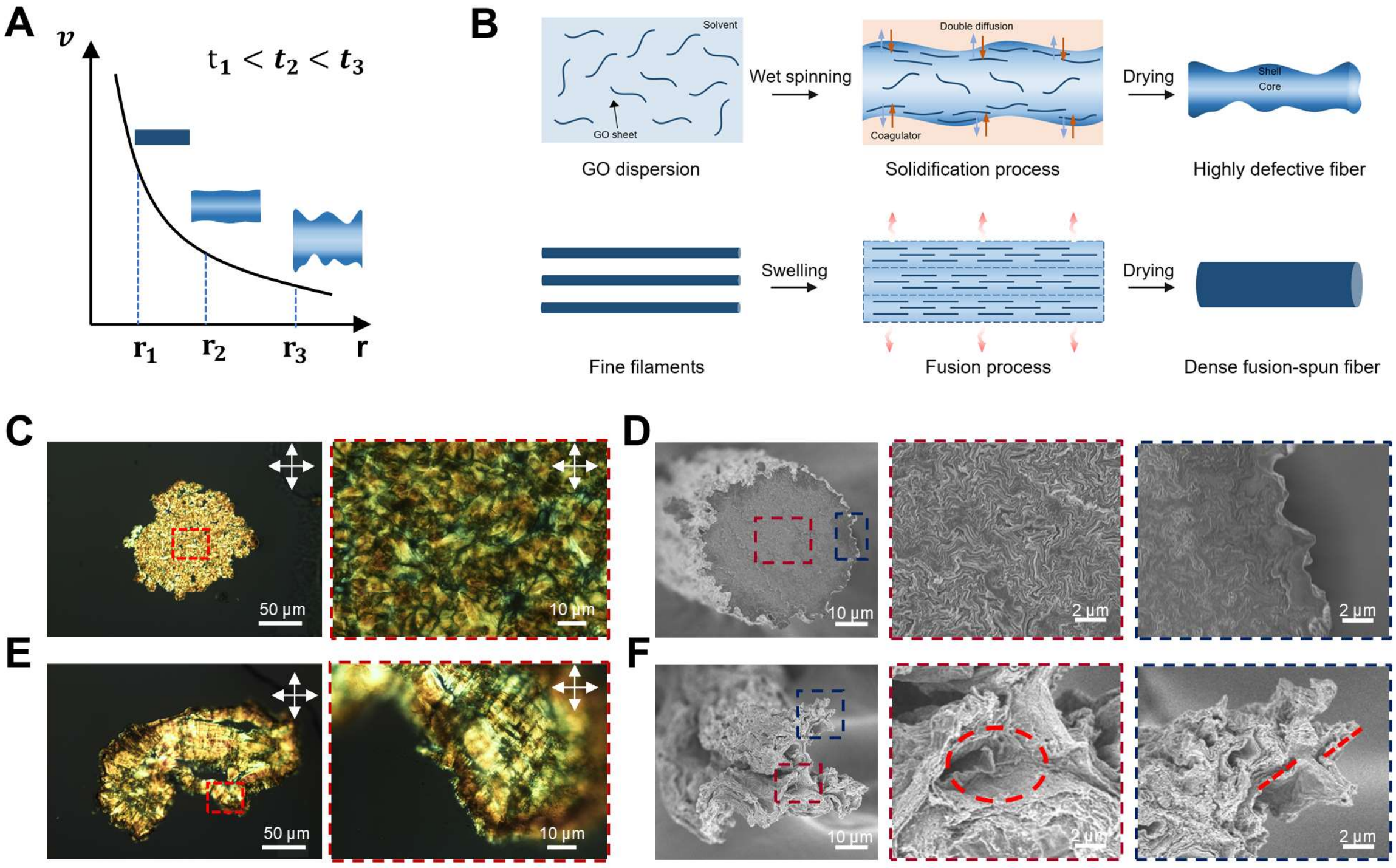

Fig. S13. Suppression of core-shell heterogeneity by multiscale fusion spinning. (**A**) Schematic illustrating the relationship between double diffusion velocity and fiber radius in conventional wet spinning. Prolonged diffusion paths in larger-diameter fibers lead to severe core-shell structural heterogeneity. (**B**) Comparative schematic of the solidification processes and resultant morphologies for FSGOF (uniform) and DSGOF (core-shell). (**C**) Polarized optical micrograph of an axially sectioned swollen FSGOF, showing uniform liquid crystal alignment. (**D**) SEM image of the FSGOF cross-section and a magnified view of its homogeneous core region (this cross-section is the same sample SEM image as Fig. 1G in the main text). (**E**) Polarized optical micrograph of an axially sectioned swollen DSGOF, revealing structural heterogeneity. (**F**) SEM image of the DSGOF cross-section and a magnified view of its pronounced core-shell defect region.

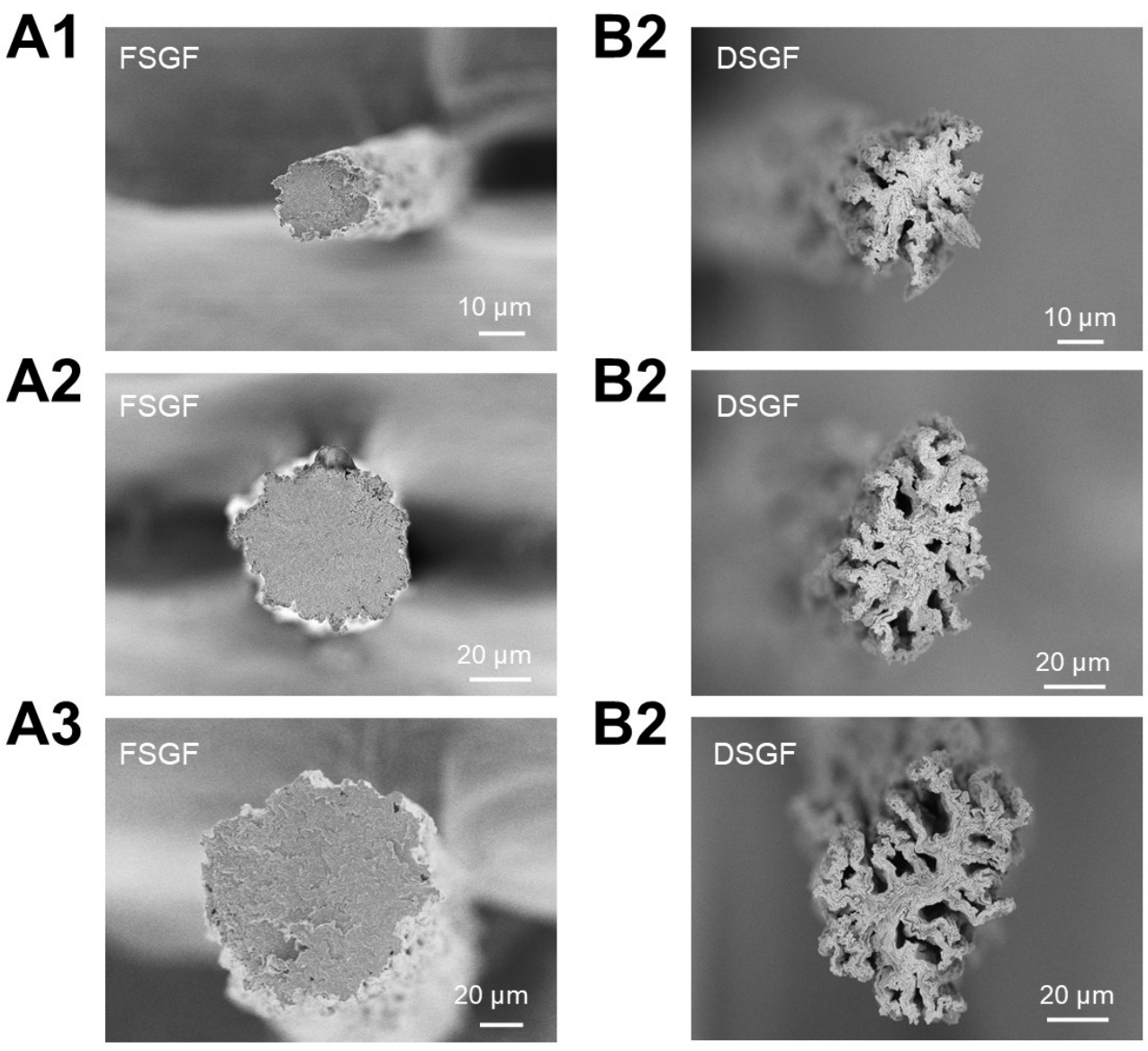

Fig. S14. Morphological comparisons between FSGF and DSGF with varying diameters. (**A1** to **A3**) SEM images of FSGF with gradually increasing diameters (A3 and Fig. 2B2 in the main text are the same sample SEM); (B1 to B3) SEM images of DSGF with gradually increasing diameters (B3 and Fig. 2C2 in the main text are the same sample SEM).

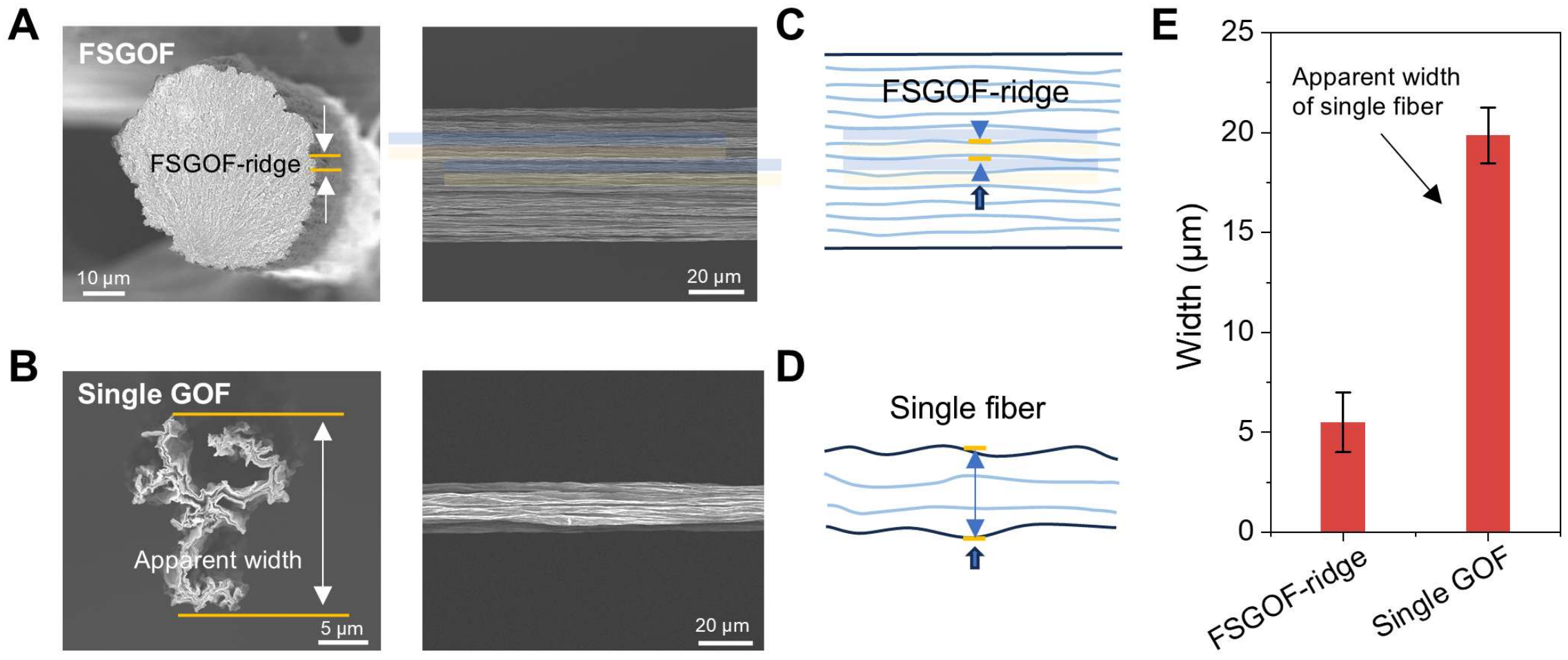

Fig. S15. Surface ridge analysis reveals the fusion of FSGOF. (**A** and **B**) SEM images of the cross-sections and surface characteristic ridges of FSGOF and a single fiber. (**C** and **D**) Schematics illustrating the measurement of ridge width of FSGOF and the apparent width of a single fiber (corresponding to the left images in A and B). (**E**) Comparison between the ridge length on FSGOF and the contour length of a single filament. The significantly shorter ridge length demonstrates that FSGOF is an entity formed by complete fusion, rather than a loose bundle of individual filaments.

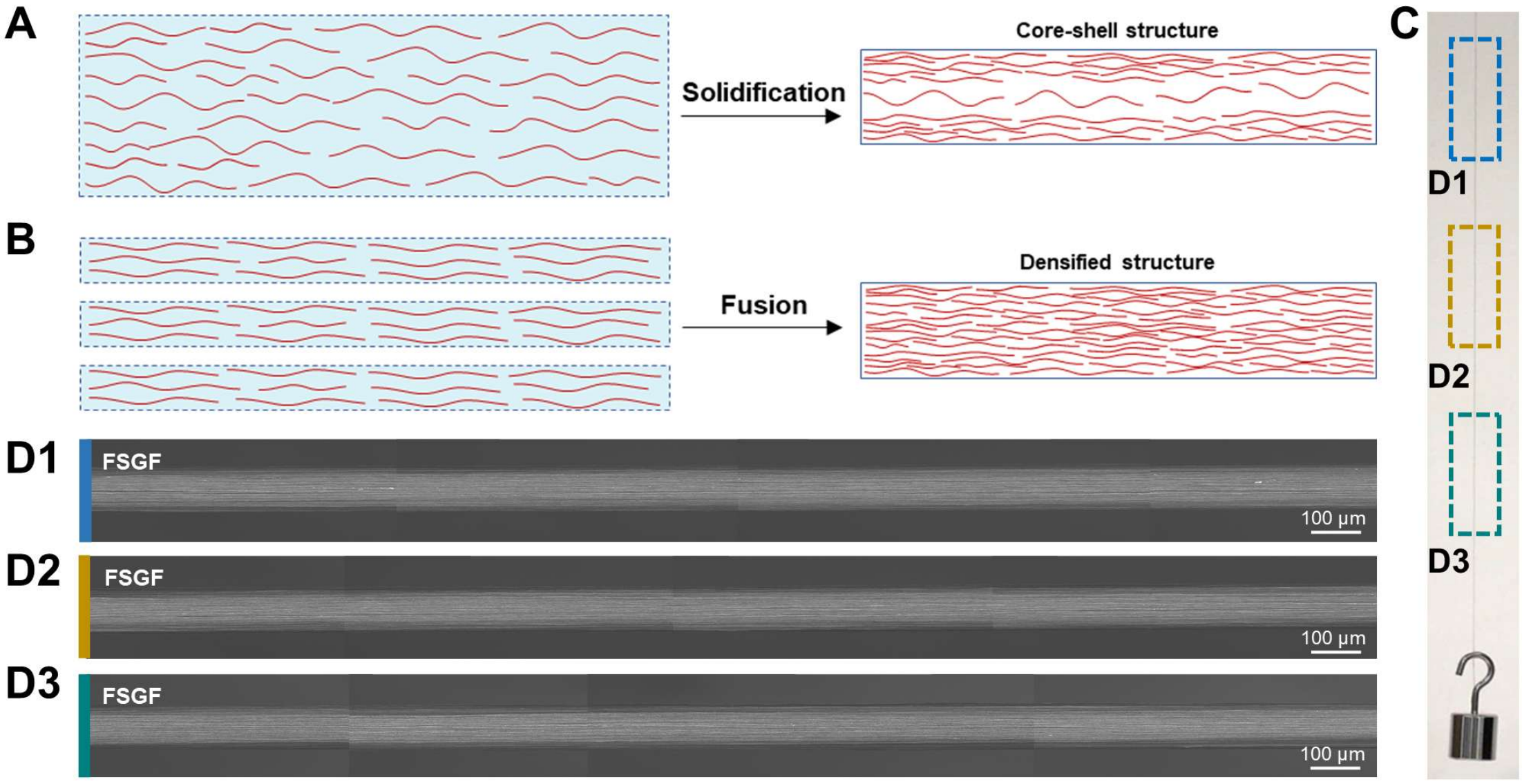

Fig. S16. Structural evolution and morphology of fibers during solidification. (**A**) Schematic depicting the sheet arrangement during solidification of a large-diameter DSGF, leading to dense shell and sparse core (core-shell heterogeneity). (**B**) Schematic illustrating the sheet rearrangement during the drying and fusion process of FSGF, resulting in uniformly dense packing without core-shell structure. (**C**) Photograph of a meter-long FSGF (diameter: 100 μm) supporting a 100 g weight. (**D1 to D3**) SEM images taken at three distinct axial positions (color-coded in (C)) of the same FSGF, demonstrating uniform cross-sectional morphology along its length (some of the SEM images are from the same sample as Fig. 2B1 in the main text).

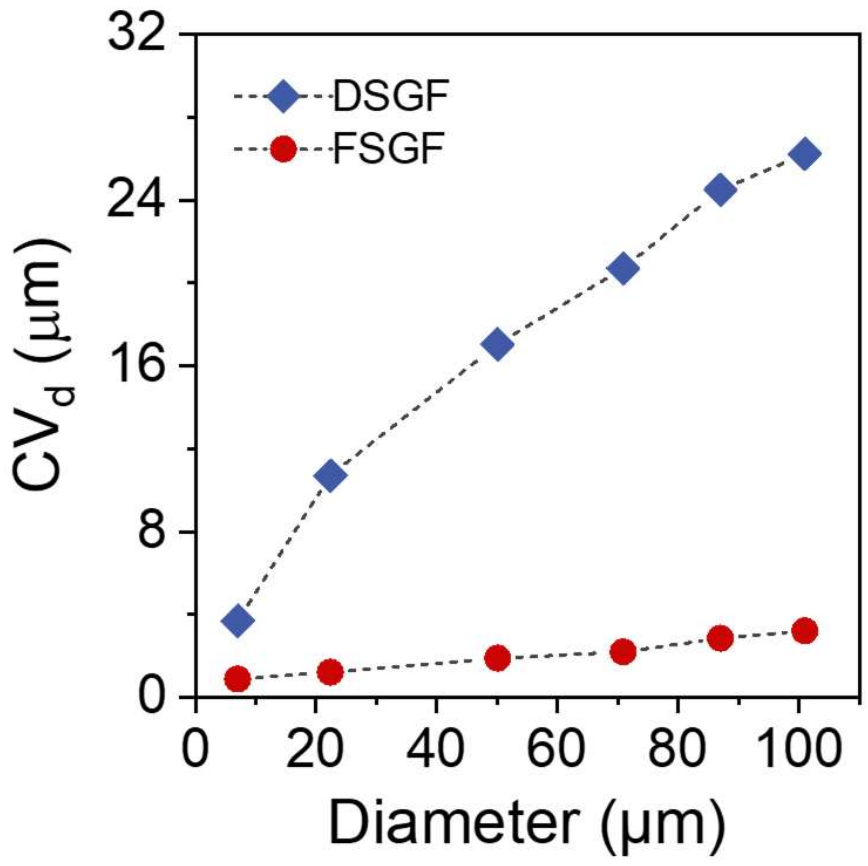

Fig. S17. Axial diameter uniformity of FSGF and DSGF.

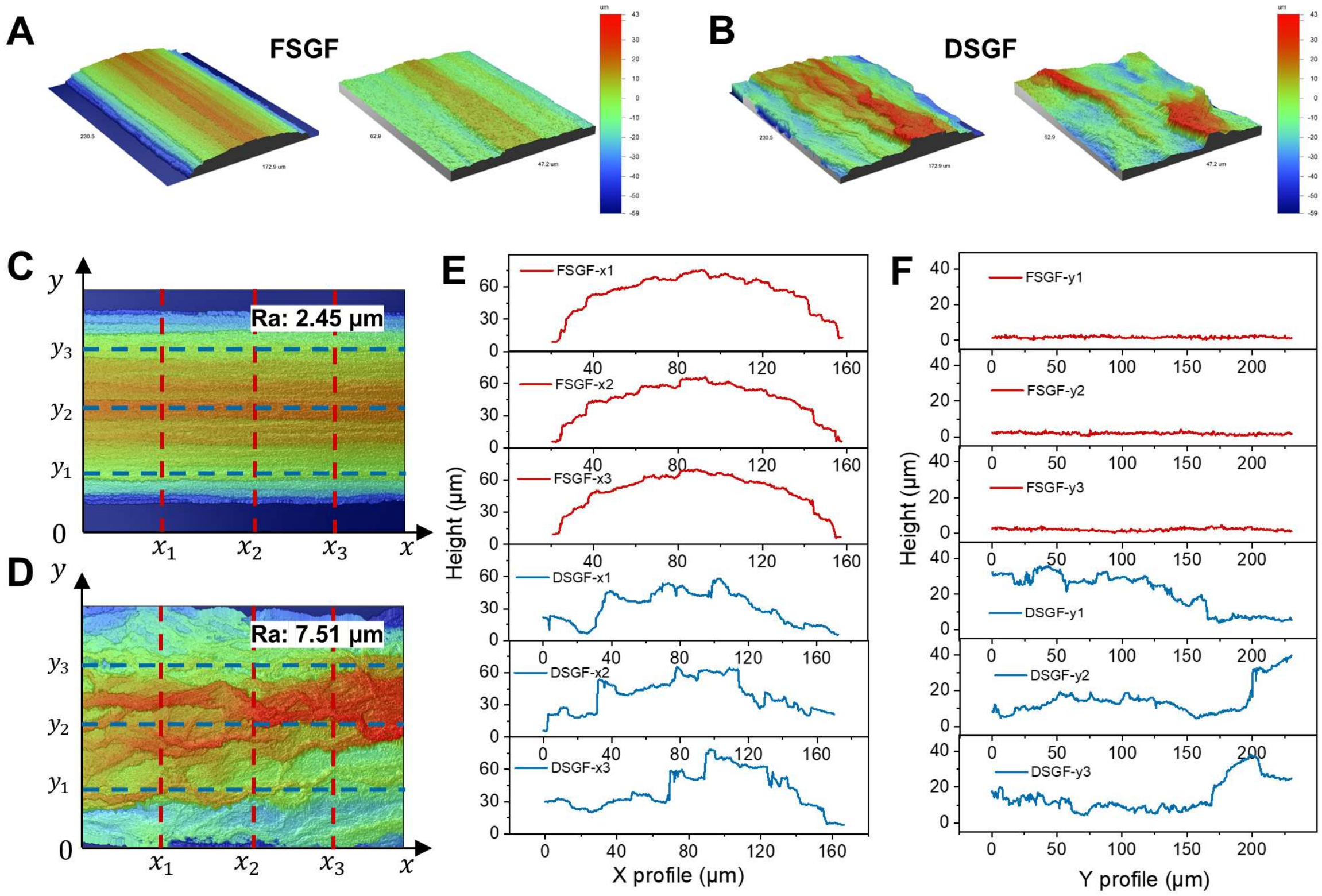

Fig. S18. Surface topography of FSGF and DSGF characterized by white light interferometry. (**A** and **B**) Three-dimensional surface reconstructions of FSGF (A) and DSGF (B). (**C** and **D**) Corresponding top-view profiles and surface roughness values (Ra) for FSGF (C) and DSGF (D). (**E** and **F**) Contour profiles measured at different axial and radial positions on FSGF (E) and DSGF (F).

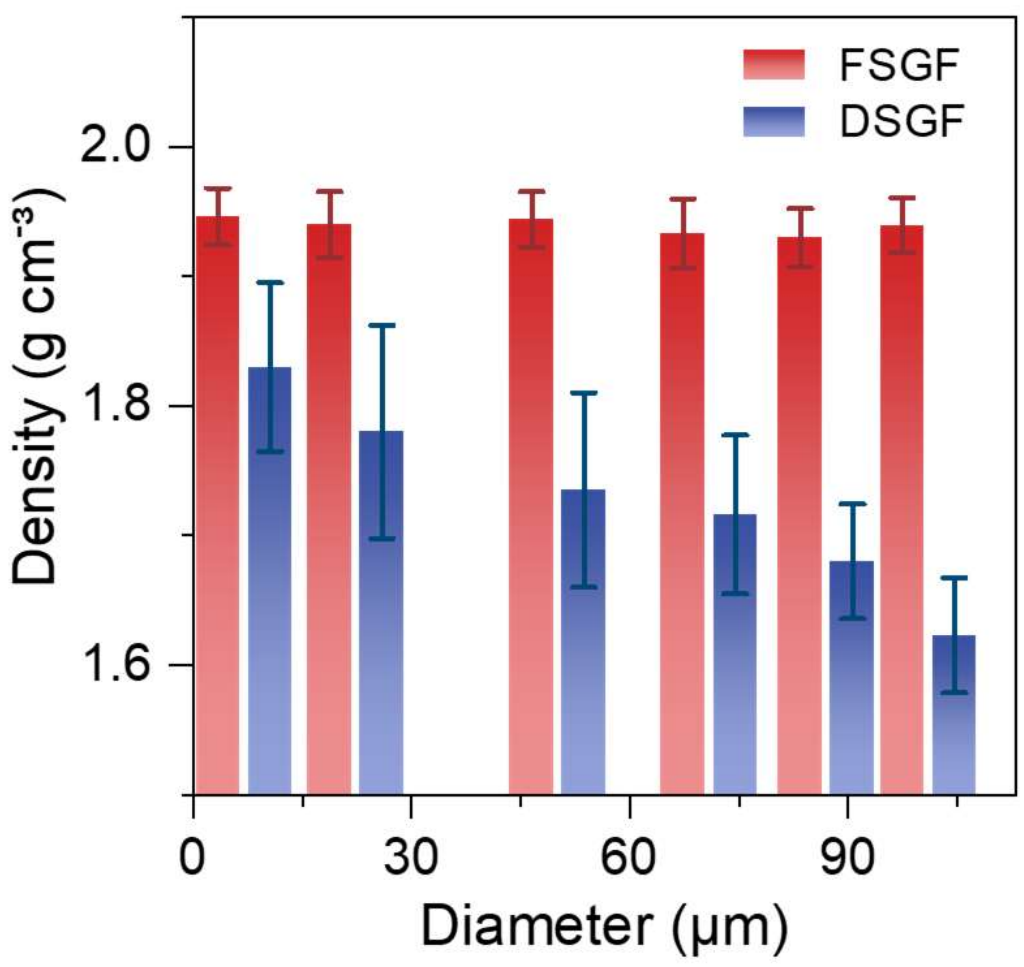

Fig. S19. Density of FSGF and DSGF of different diameters.

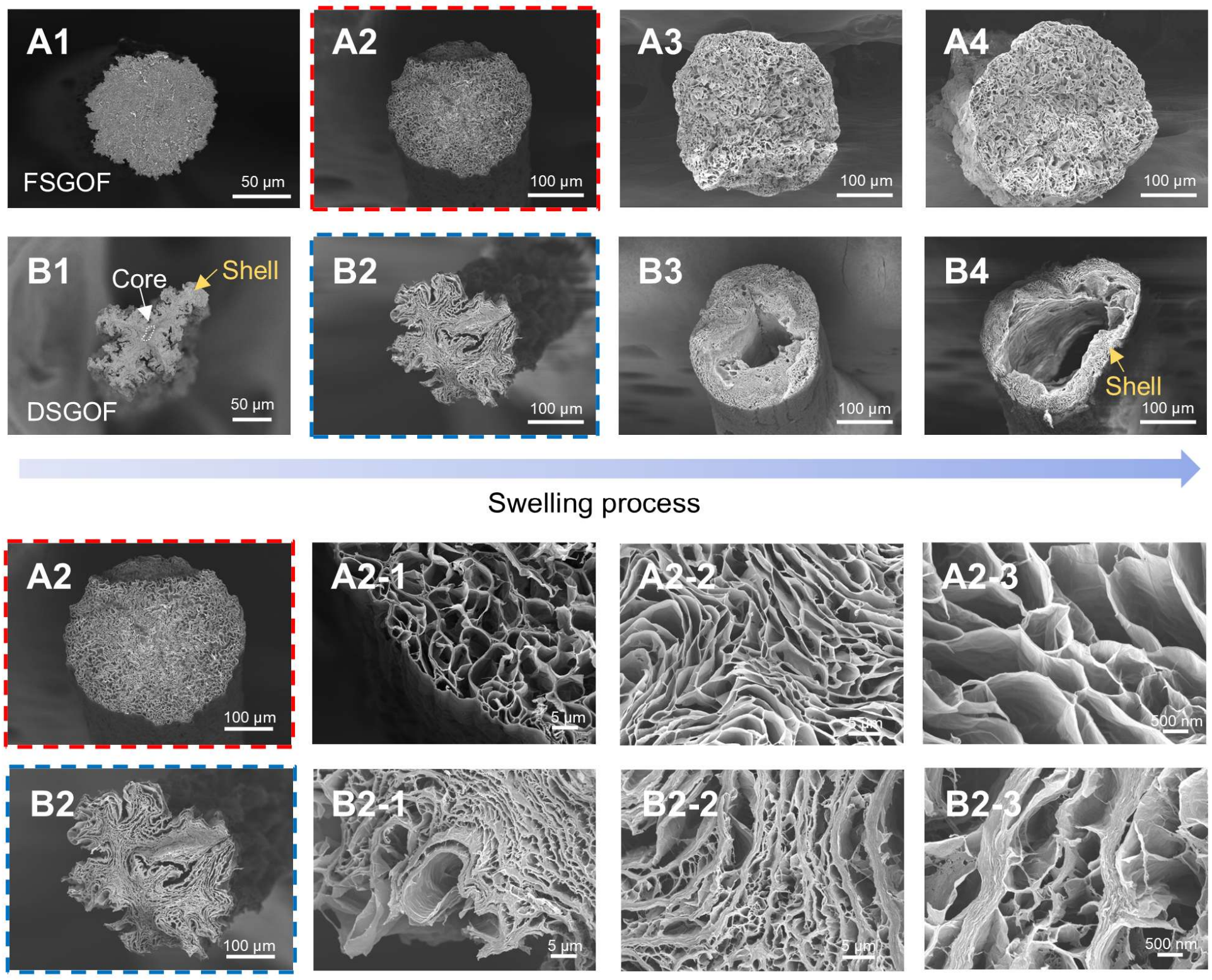

Fig. S20. Structural homogeneity of FSGOF versus DSGOF upon swelling. (**A1** to **A4** and **B1** to **B4**) Cross-sectional SEM images of freeze-dried FSGOF (A1 to A4) and DSGOF (B1 to B4) at different swelling ratios (0%, 50%, 100%, 200%), revealing the persistence of a core-shell structure in DSGOF. (**A2-1** to **A2-3**) Representative SEM images of a 50% swollen, freeze-dried FSGOF: sheet arrangement in the shell region (A2-1), sheet arrangement in the core region (A2-2), and uniformly stacked GO lamellae (A2-3), demonstrating structural homogeneity. (**B2-1** to **B2-3**) Representative SEM images of a 50% swollen, freeze-dried DSGOF: densely packed sheets in the shell region (B2-1), loosely packed sheets in the core region (B2-2), and unevenly stacked GO lamellae (B2-3), highlighting the distinct core-shell heterogeneity.

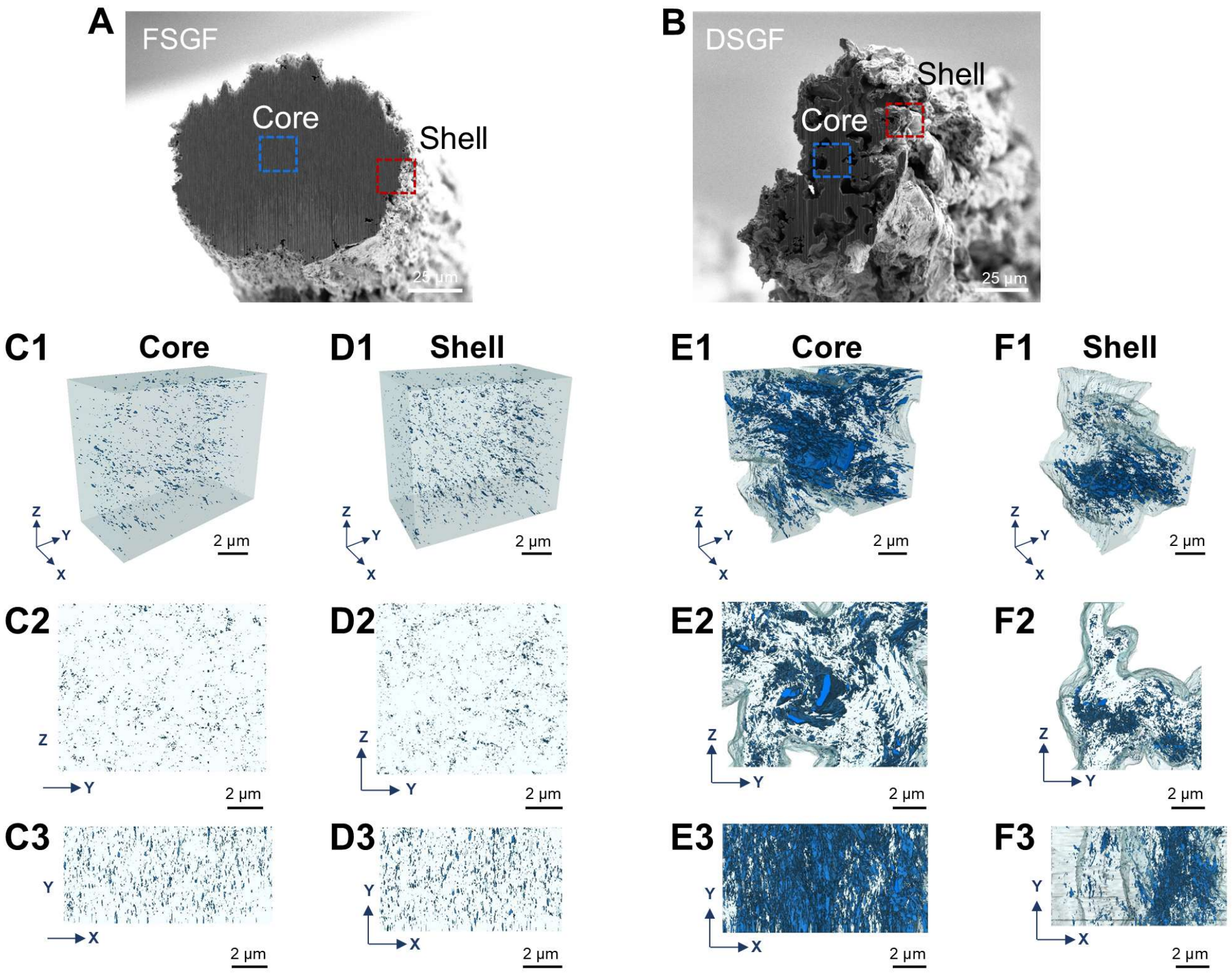

Fig. S21. Three-dimensional structural analysis of FSGF and DSGF by focused ion beam-scanning electron microscopy. (**A** and **B**) Cross-sectional SEM images of FSGF (A) and DSGF (B) acquired via FIB milling, indicating the regions for 3D reconstruction. (**C** and **D**) 3D-rendered models of the core (C) and shell (D) volumes in FSGF, presented as perspective (C1 and D1), top (C2 and D2), and side (C3 and D3) views, demonstrating structural uniformity. (**E** and **F**) 3D-rendered models of the core (E) and shell (F) volumes in DSGF, presented as perspective (E1 and F1), top (E2 and F2), and side (E3 and F3) views, revealing pronounced structural heterogeneity between core and shell regions.

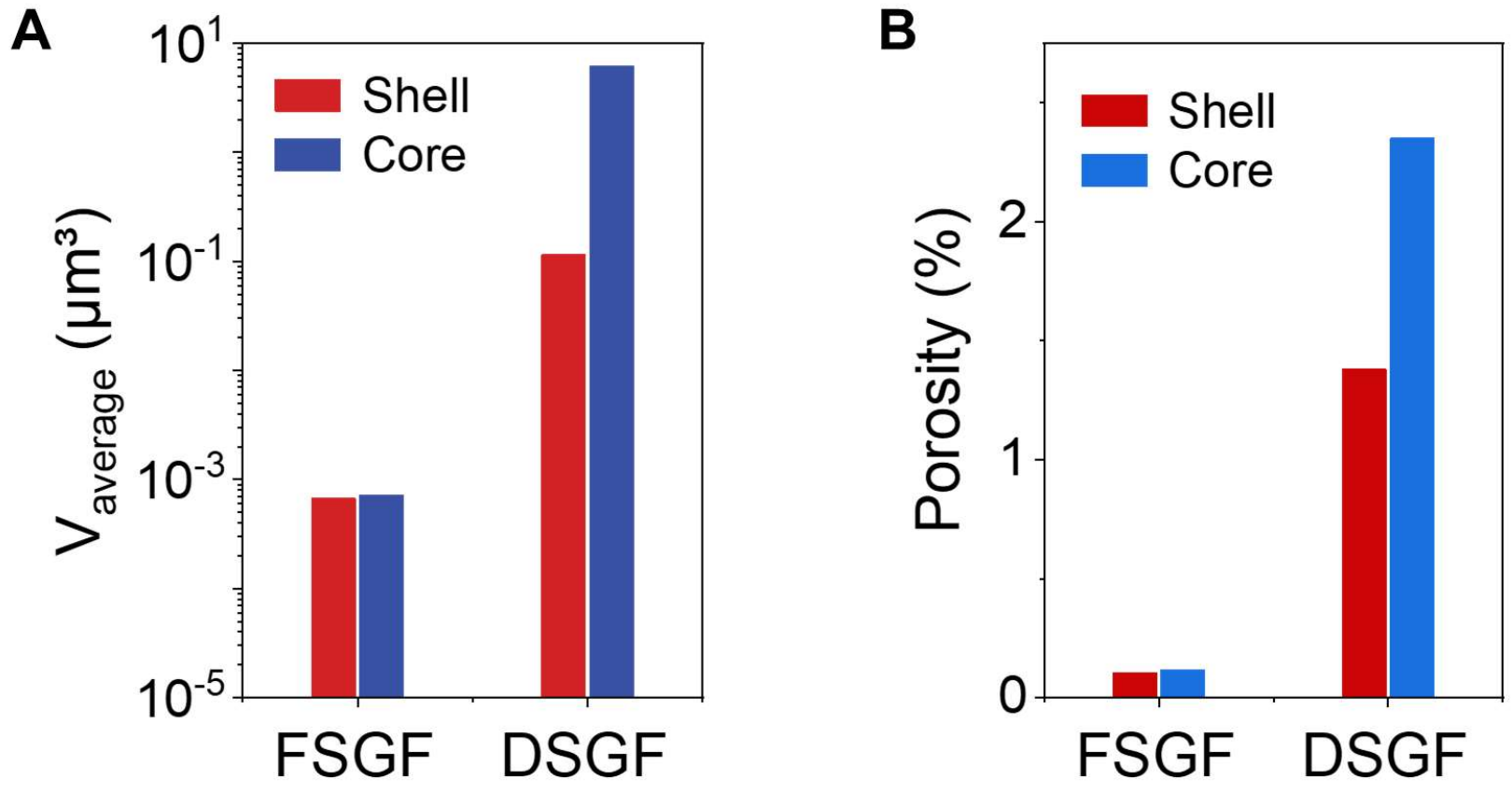

Fig. S22. Quantitative analysis of pore structure in FSGF and DSGF. (**A**) Comparison of average void volume ($V_{average}$) in the core and shell regions of FSGF and DSGF. (**B**) Comparison of porosity in the core and shell regions of FSGF and DSGF.

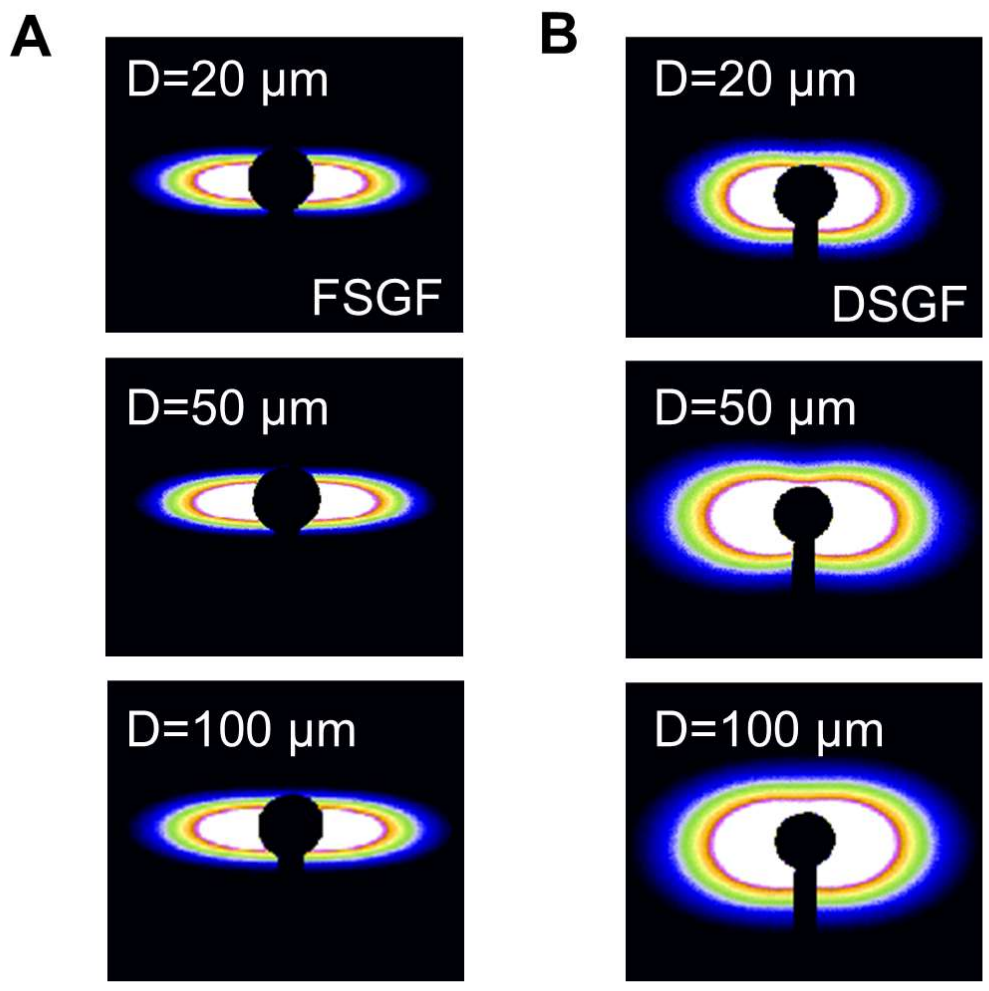

Fig. S23. SAXS 2D patterns of FSGF (A) and DSGF (B) with varying diameters.

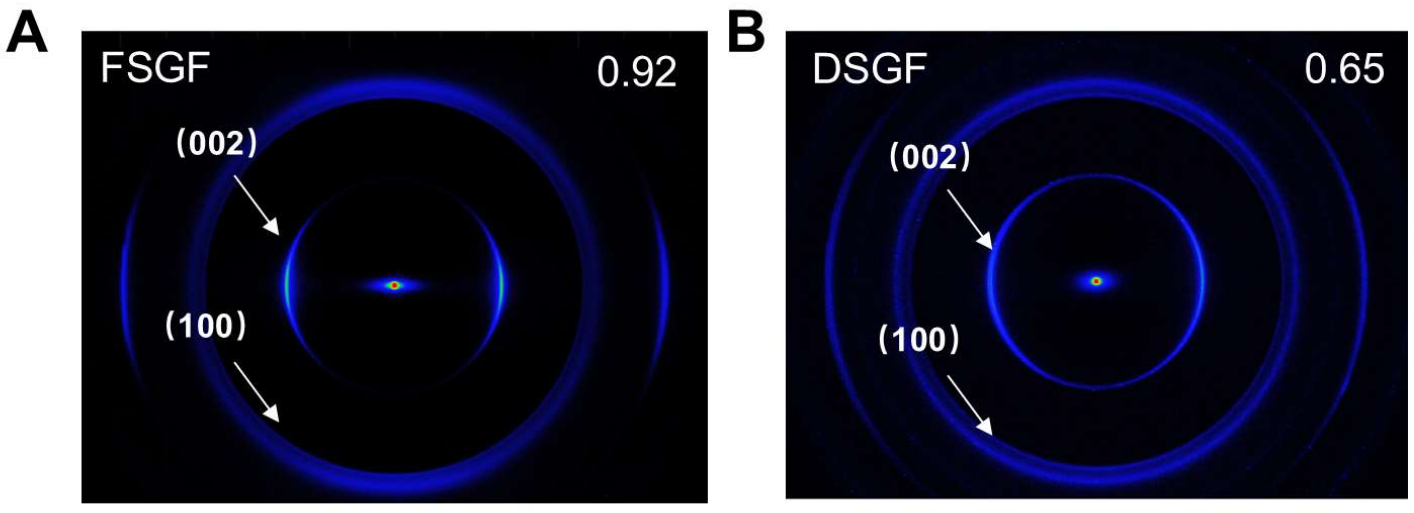

Fig. S24. WAXS 2D patterns of FSGF (A) and DSGF (B) with diameter of 100 μm.

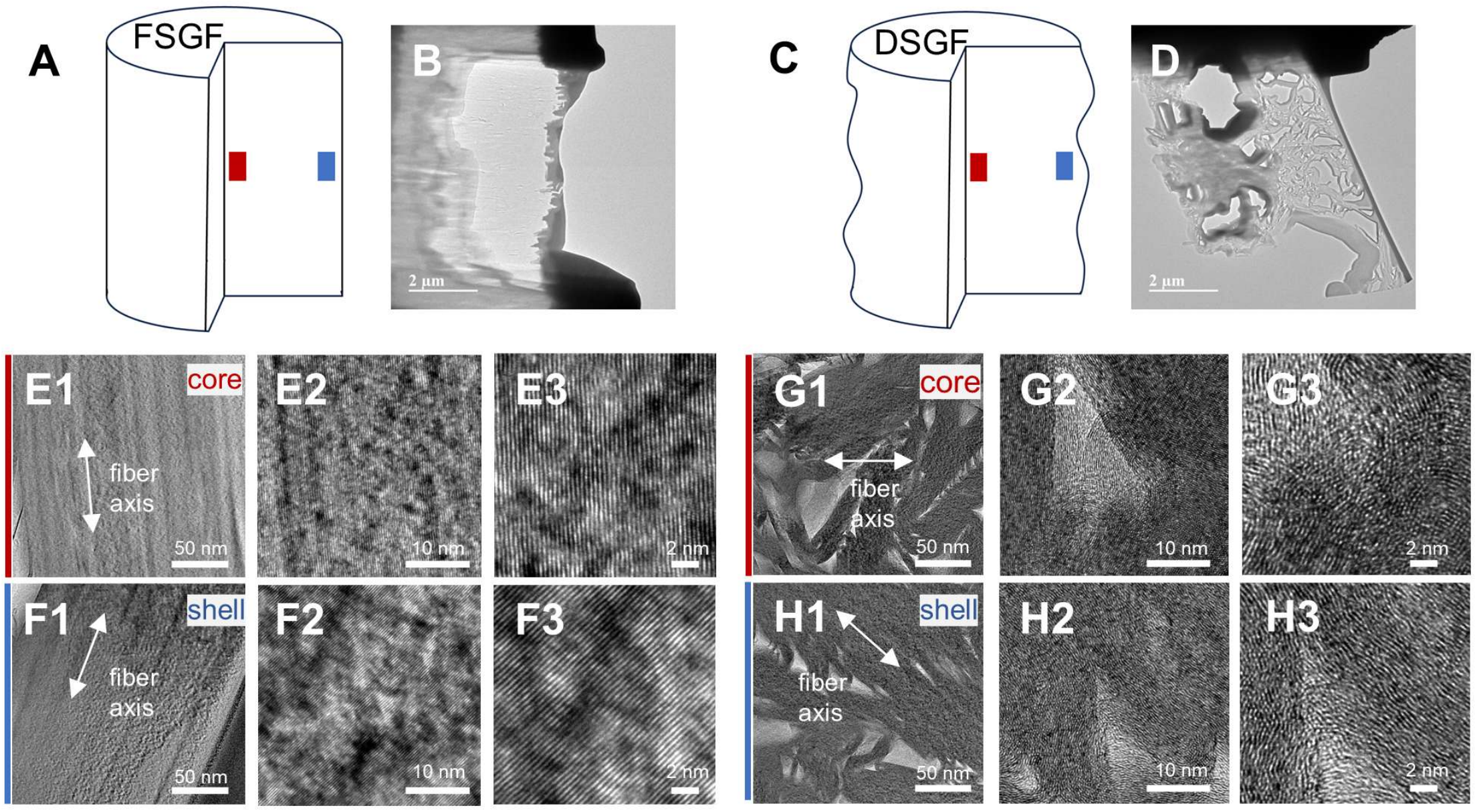

Fig. S25. Comparative analysis of core-shell structural differences in FSGF and DSGF by HR-TEM. (**A**) Schematic illustrating the core and shell sampling regions (red and blue, respectively) in FSGF. (**B**) SEM image of the FIB-milled axial cross-section of FSGF. (**C**) Schematic illustrating the core and shell sampling regions in DSGF. (**D**) SEM image of the FIB-milled axial cross-section of DSGF. (**E1** to **E3** and **F1** to **F3**) Representative HR-TEM images of the core (E1 to E3) and shell (F1 to F3) regions in FSGF (E1 and E3 are the same TEM image as Fig. 2K in the main text). (**G1** to **G3** and **H1** to **H3**) Representative HR-TEM images of the core (G1 to G3) and shell (H1 to H3) regions in DSGF (G1 and G3 are the same TEM image as Fig. 2K in the main text).

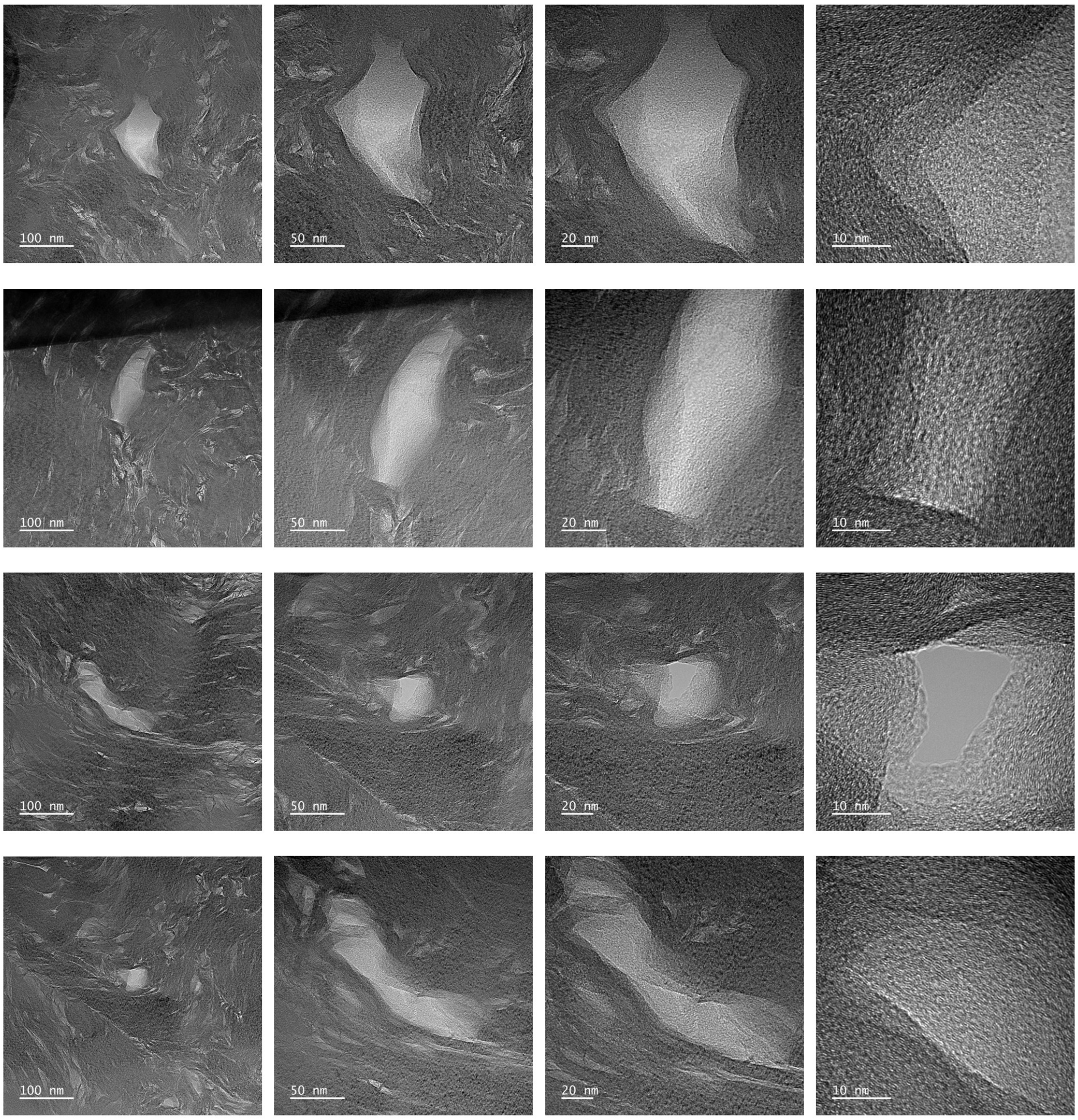

Fig. S26. TEM image of a focused ion beam (FIB) sectioned radial cross-section of DSGF.

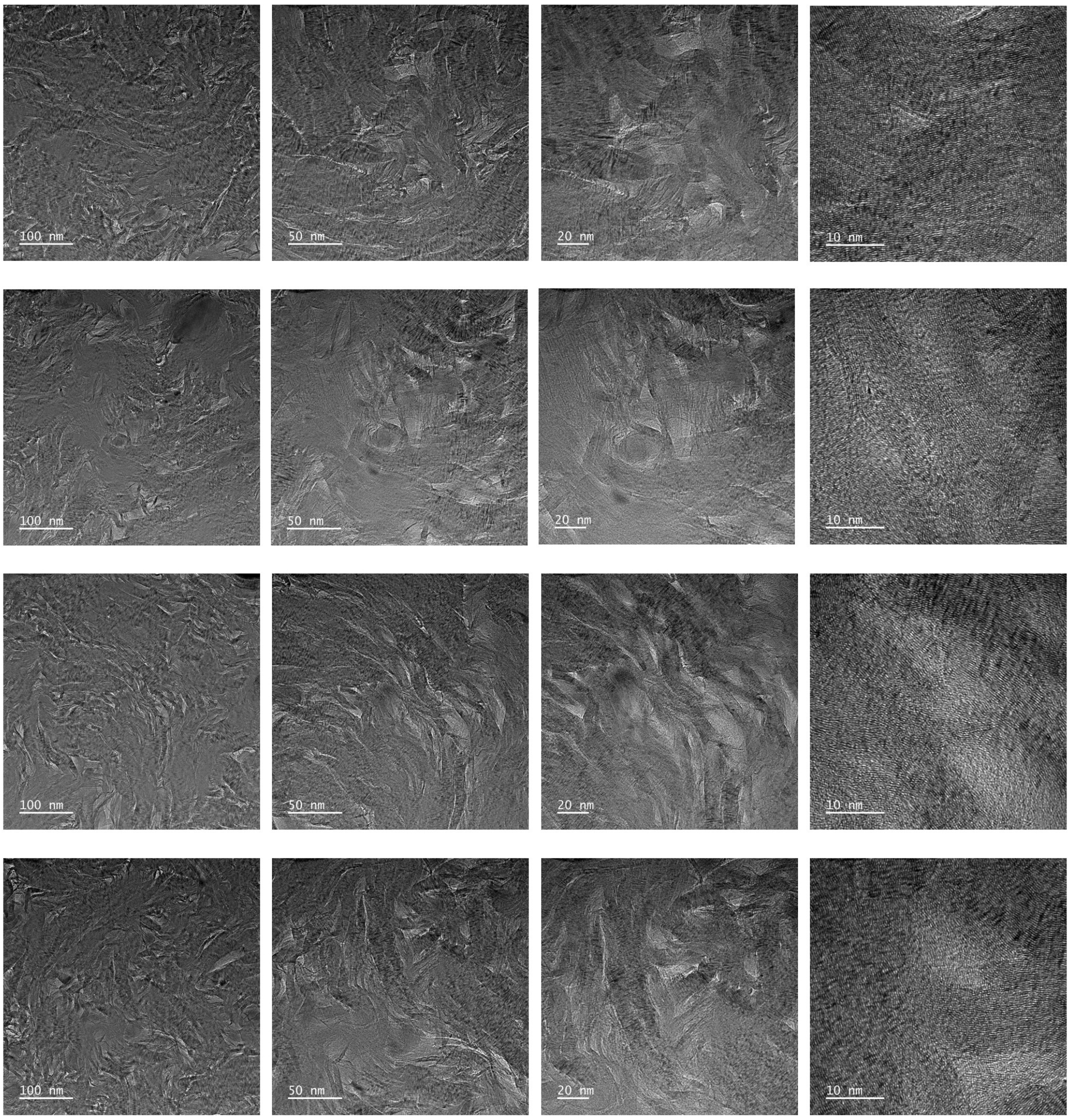

Fig. S27. TEM image of a focused ion beam (FIB) sectioned radial cross-section of FSGF.

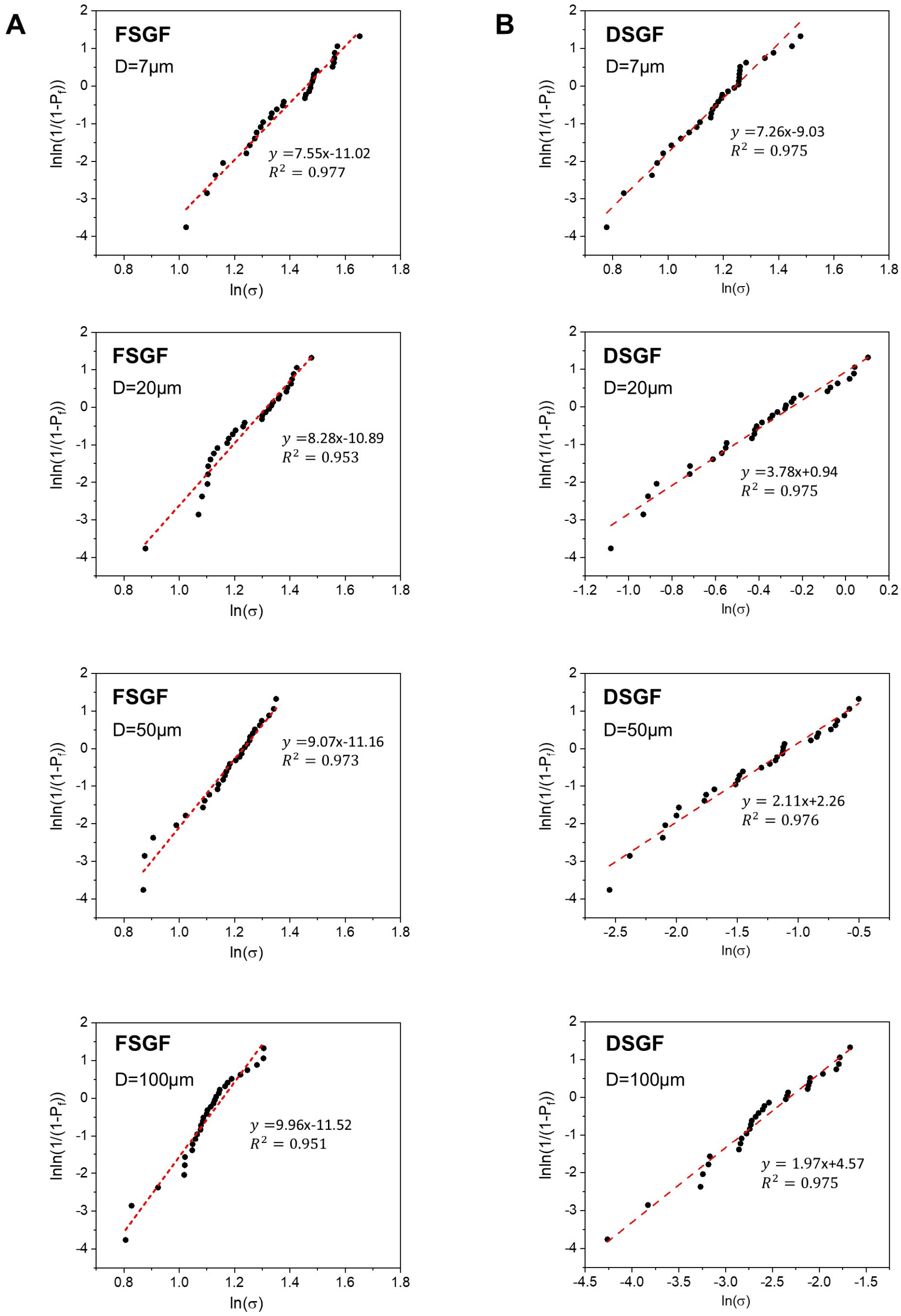

Fig. S28. Individual Weibull plot of failure stress of fibers for different diameters of FSGF and DSGF. The dashed lines represent linear fit lines.

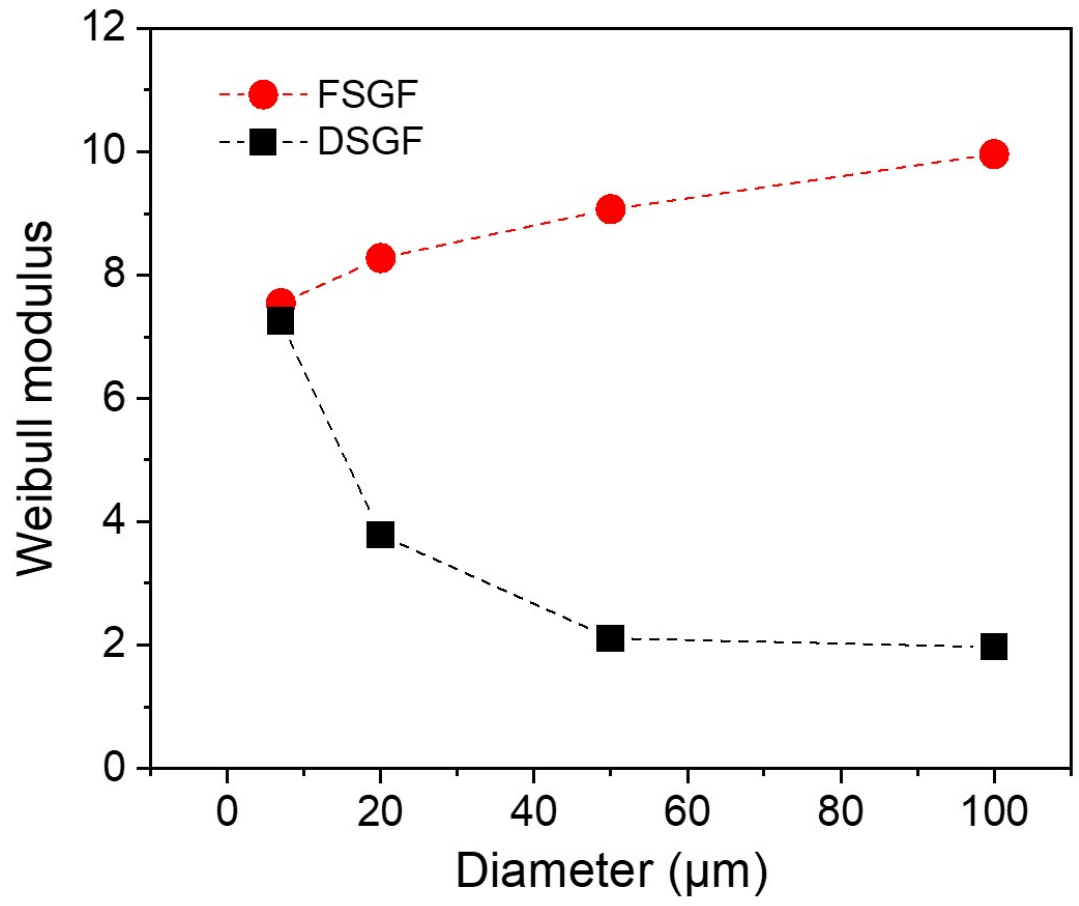

Fig. S29. Weibull modulus of FSGF and DSGF with different diameters.

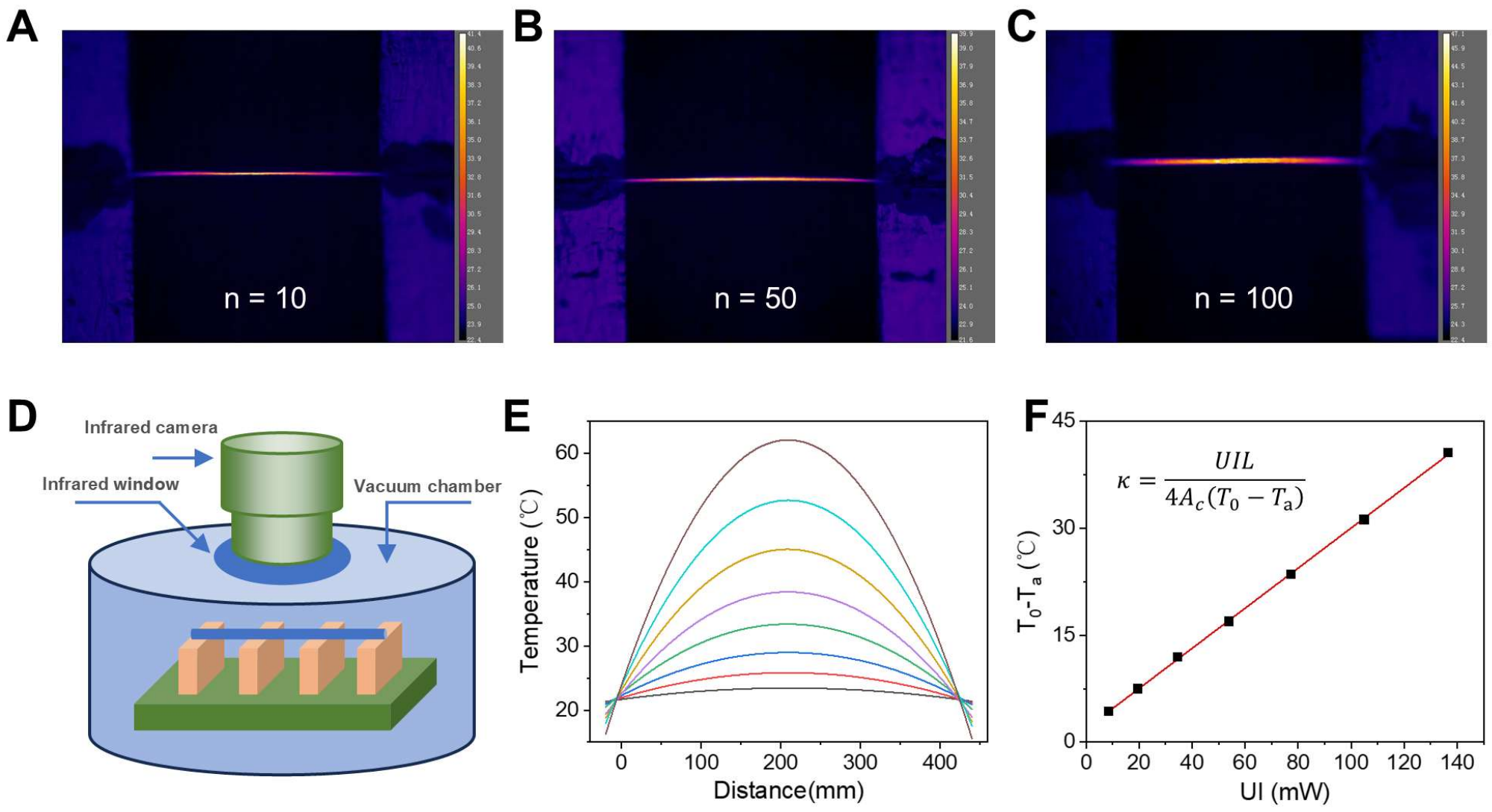

Fig. S30. Thermal conductivity of FSGF of different diameters. (**A** to **C**) Representative infrared thermography images of FSGF with diameters of 20 μm (A), 50 μm (B), and 100 μm (C) during steady-state electro-thermal measurement. (**D**) Schematic of the custom-built setup for steady-state thermal conductivity measurement. (**E** and **F**) Data processing workflow (E) and the governing formula (F) for calculating thermal conductivity from the steady-state temperature profile and electrical input.

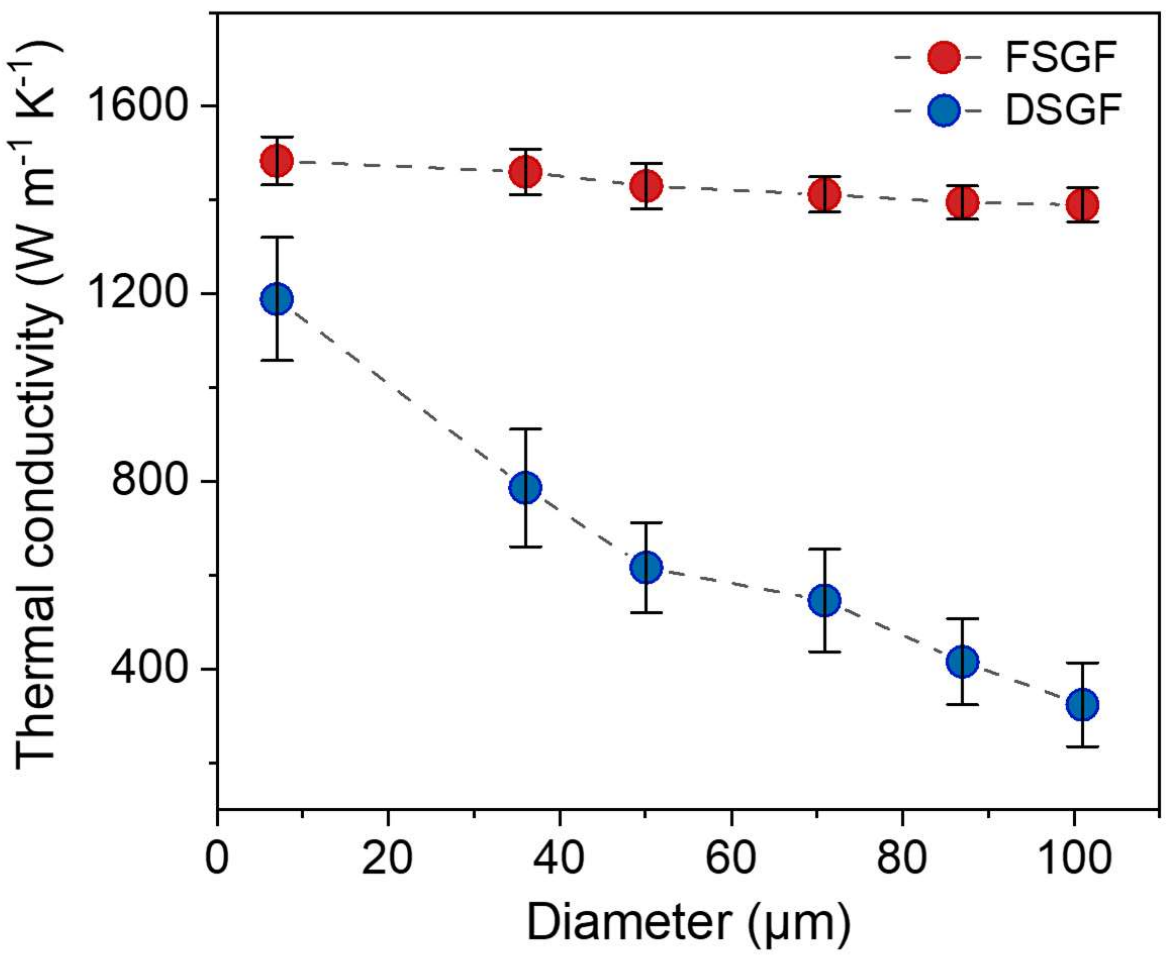

Fig. S31. Thermal conductivity versus fiber diameter for FSGF and DSGF.

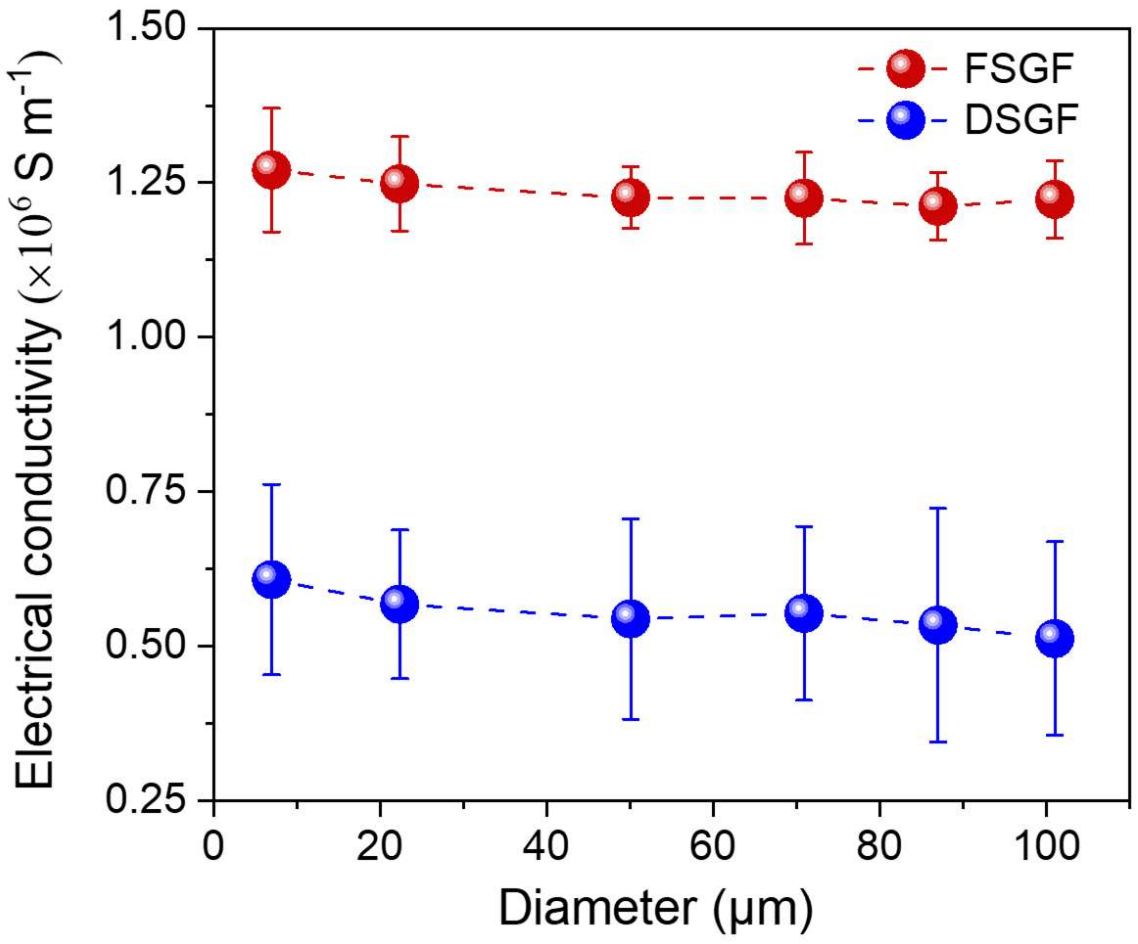

Fig. S32. Electrical conductivity versus fiber diameter for FSGF and DSGF.

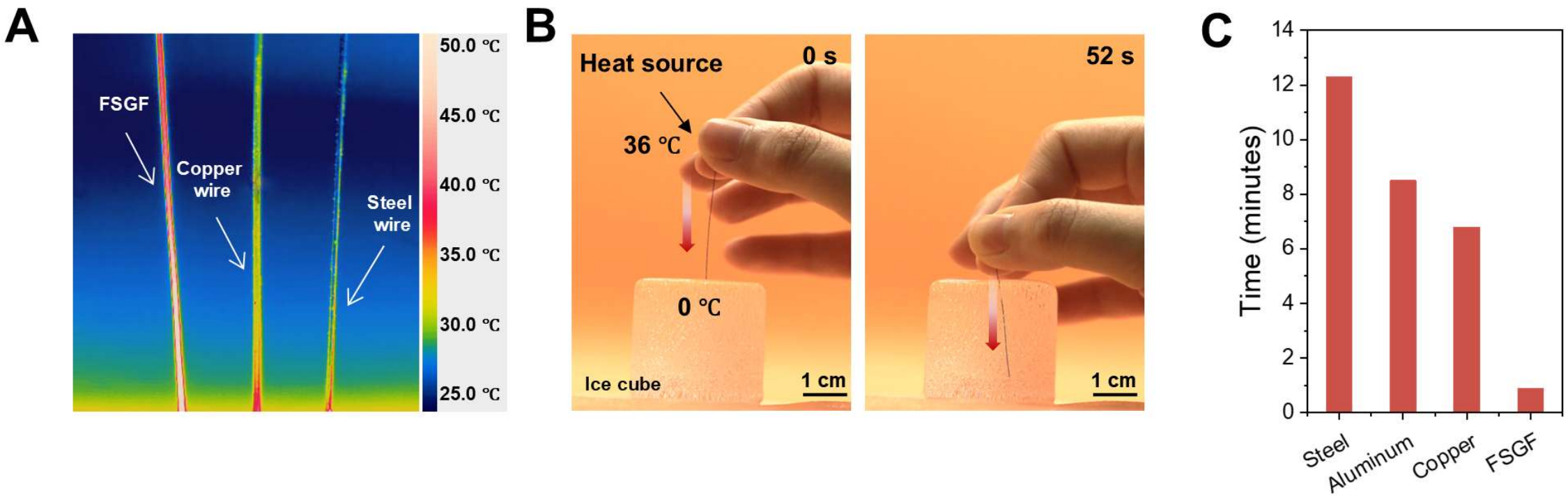

Fig. S33. Comparison of thermal transport and ice-melting penetration performance between FSGF and metal wires. (**A**) Infrared thermograph showing the temperature distribution along a 100 μm FSGF, copper wire, and steel wire (each 100 μm in diameter) attached to a microheater. The color scale (right) corresponds to temperature in °C. (**B**) Schematic and photographic sequence of ice penetration by a handheld FSGF, demonstrating heat transfer from the hand to melt ice and achieve a 2 cm depth in 52 s. (**C**) Comparison of the time required for FSGF and for steel, aluminum, and copper wires (all 100 μm diameter) to penetrate an ice block to the same depth under identical conditions.

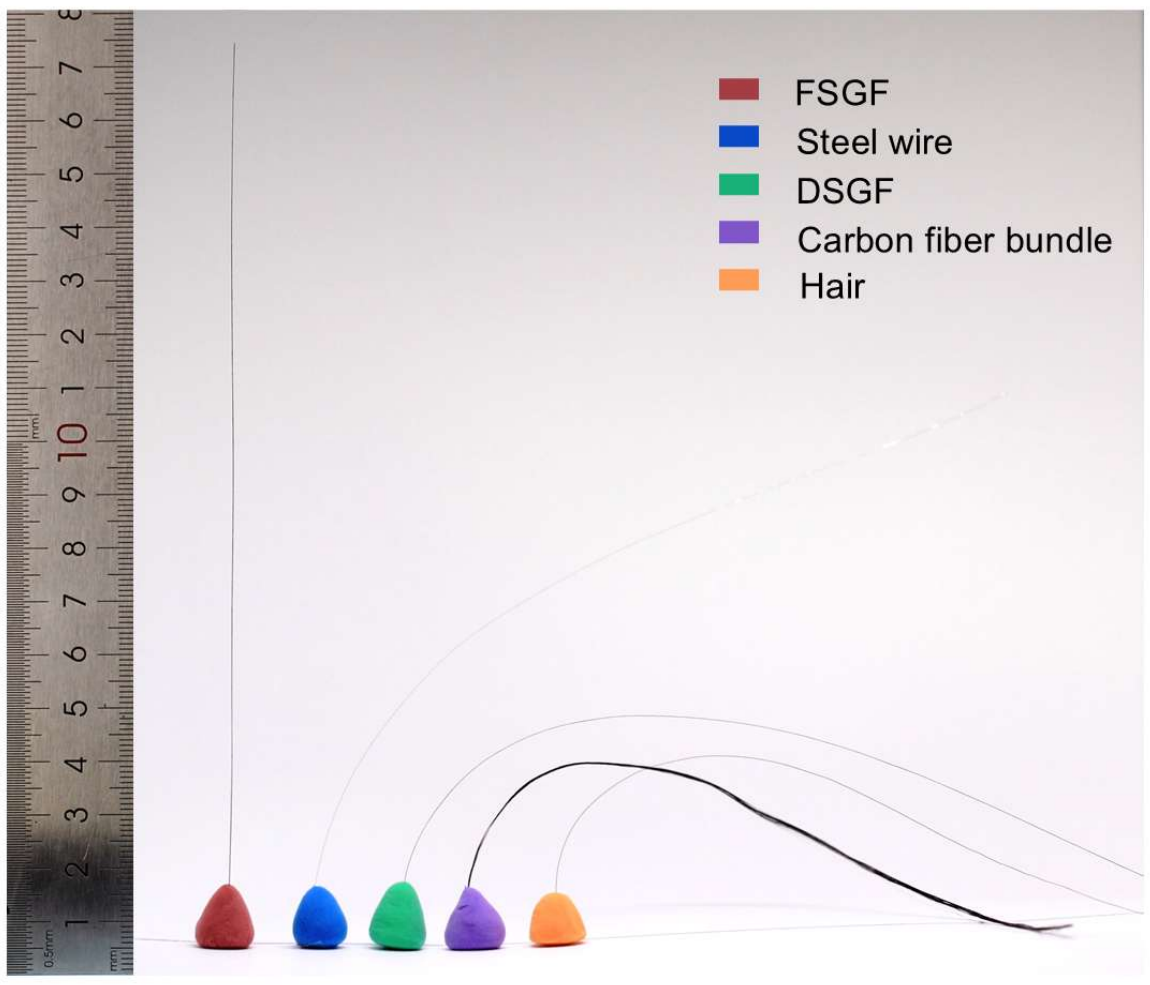

Fig. S34. Self-supporting digital photograph of FSGF and other types of fibers (this image is the original photograph of Fig. 3F).

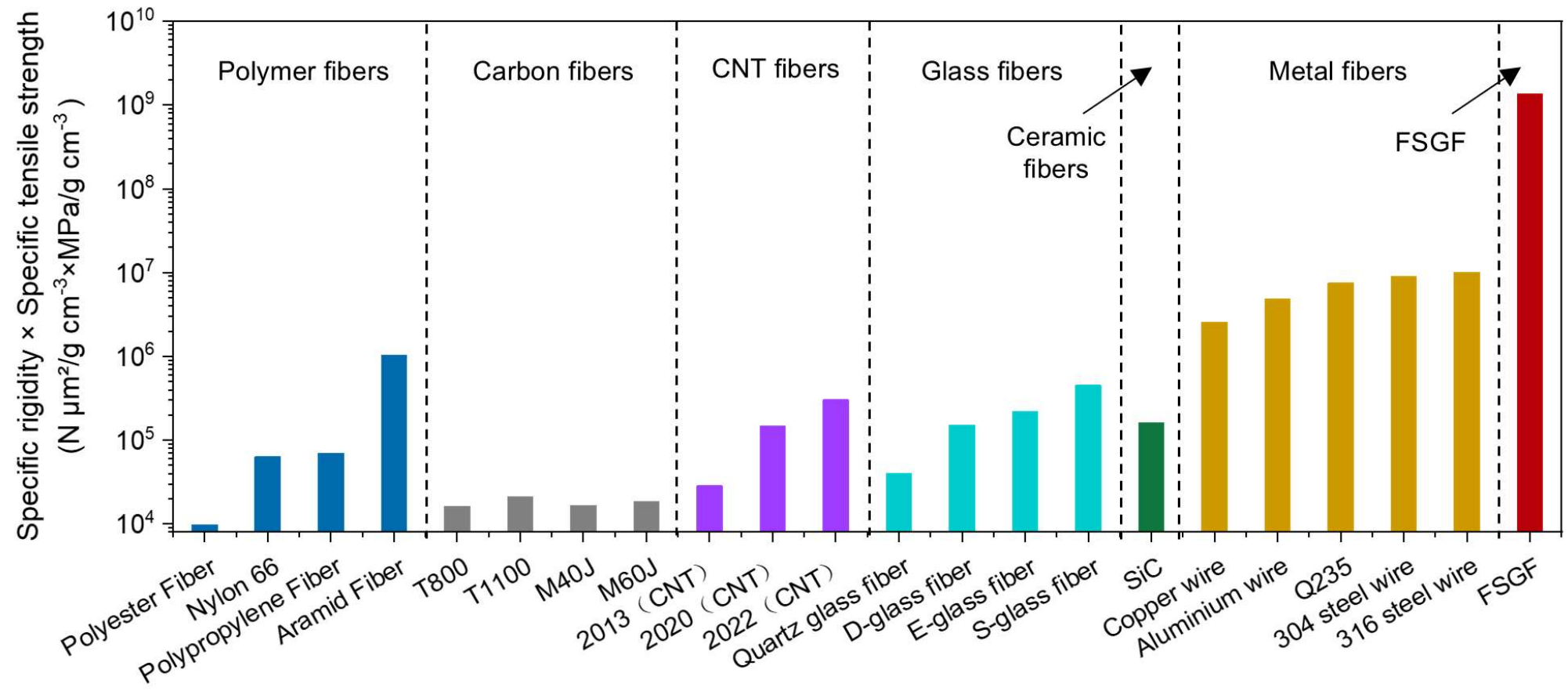

Fig. S35. Comparative analysis of the product of specific tensile strength and specific bending rigidity among various fiber categories.

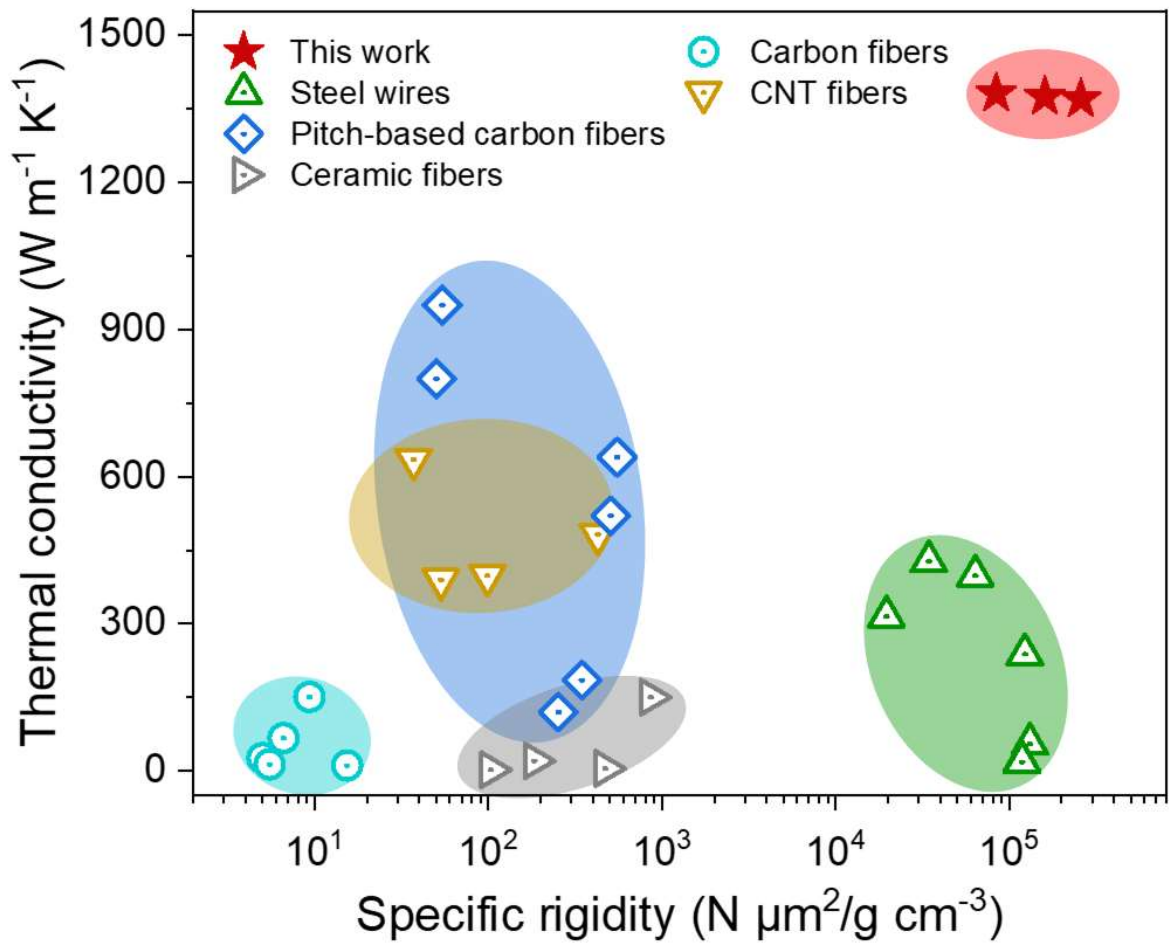

Fig. S36. Ashby plots of specific rigidity and thermal conductivity, comparing FSGF with various fibers including carbon fibers, silks, steel wires, CNT fibers, pitch-fibers, metal fibers, and ceramic fibers.

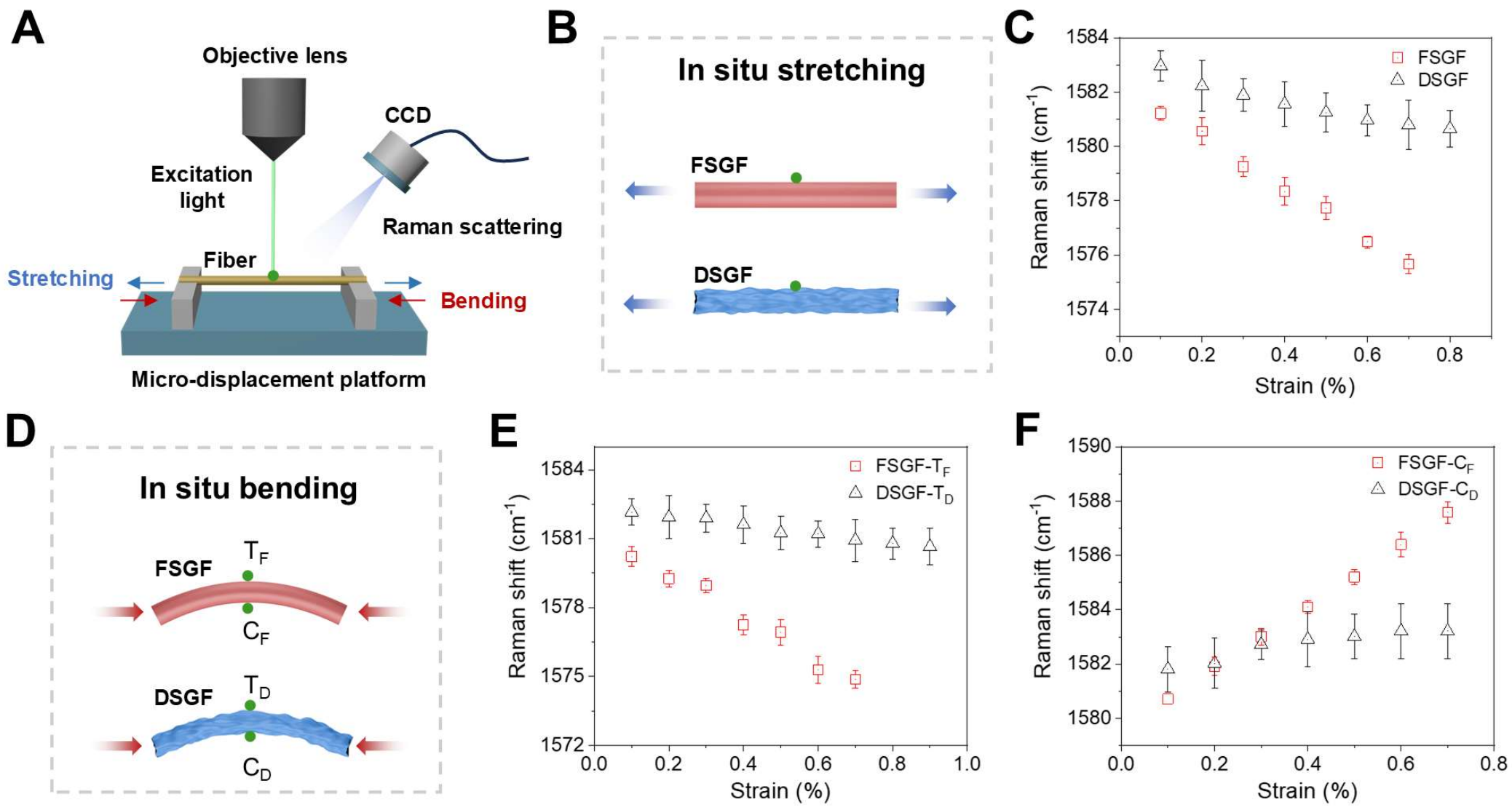

Fig. S37. In situ Raman analysis of FSGF and DSGF under tensile and bending strains. (**A**) Schematic of the custom-built setup for in situ Raman spectroscopy during mechanical testing. (**B**) Schematic of the tensile testing configuration, with the green dot indicating the laser measurement spot. (**C**) Raman G-band shifts as a function of tensile strain for FSGF and DSGF. (**D**) Schematic of the bending (compression/tension) configuration. Measurement points are labeled: TF and TD on the tensile side, CF and CD on the compressive side for FSGF and DSGF, respectively. (**E** and **F**) Corresponding Raman G-band shifts measured on the tensile side (E) and the compressive side (F) during bending.

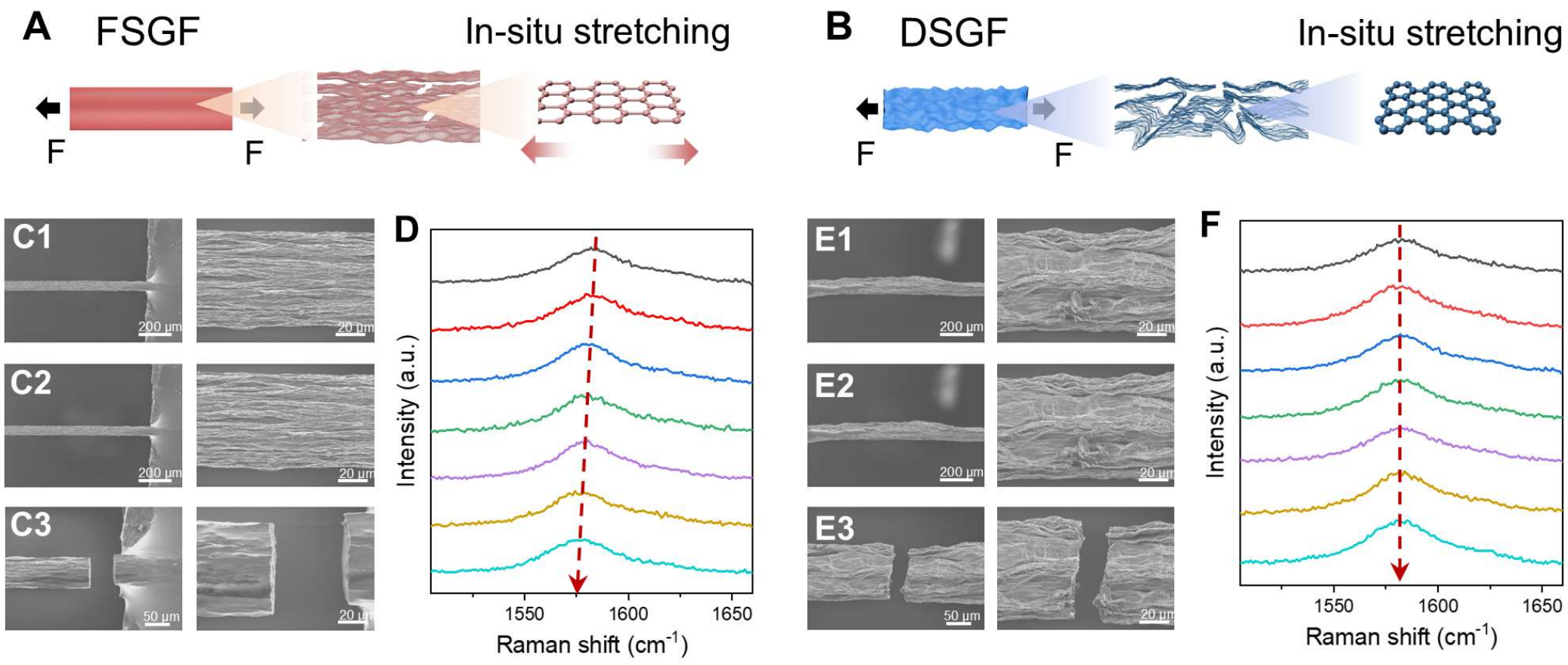

Fig. S38. In situ tensile fracture behavior and Raman response of FSGF and DSGF. (**A** and **B**) Schematics illustrating the evolution of internal sheet arrangement during tensile loading for FSGF (A) and DSGF (B). (**C1** to **C3**) Sequential in situ SEM images capturing the tensile fracture process of FSGF, showing a neat cross-section and brittle fracture mode. (**D**) Corresponding in situ Raman spectra tracking the G-band shift to lower wavenumbers with increasing tensile strain for FSGF. (**E1** to **E3**) Sequential in situ SEM images capturing the tensile fracture process of DSGF, showing an irregular cross-section and ductile fracture mode. (**F**) Corresponding in situ Raman spectra showing negligible G-band shift for DSGF under tension.

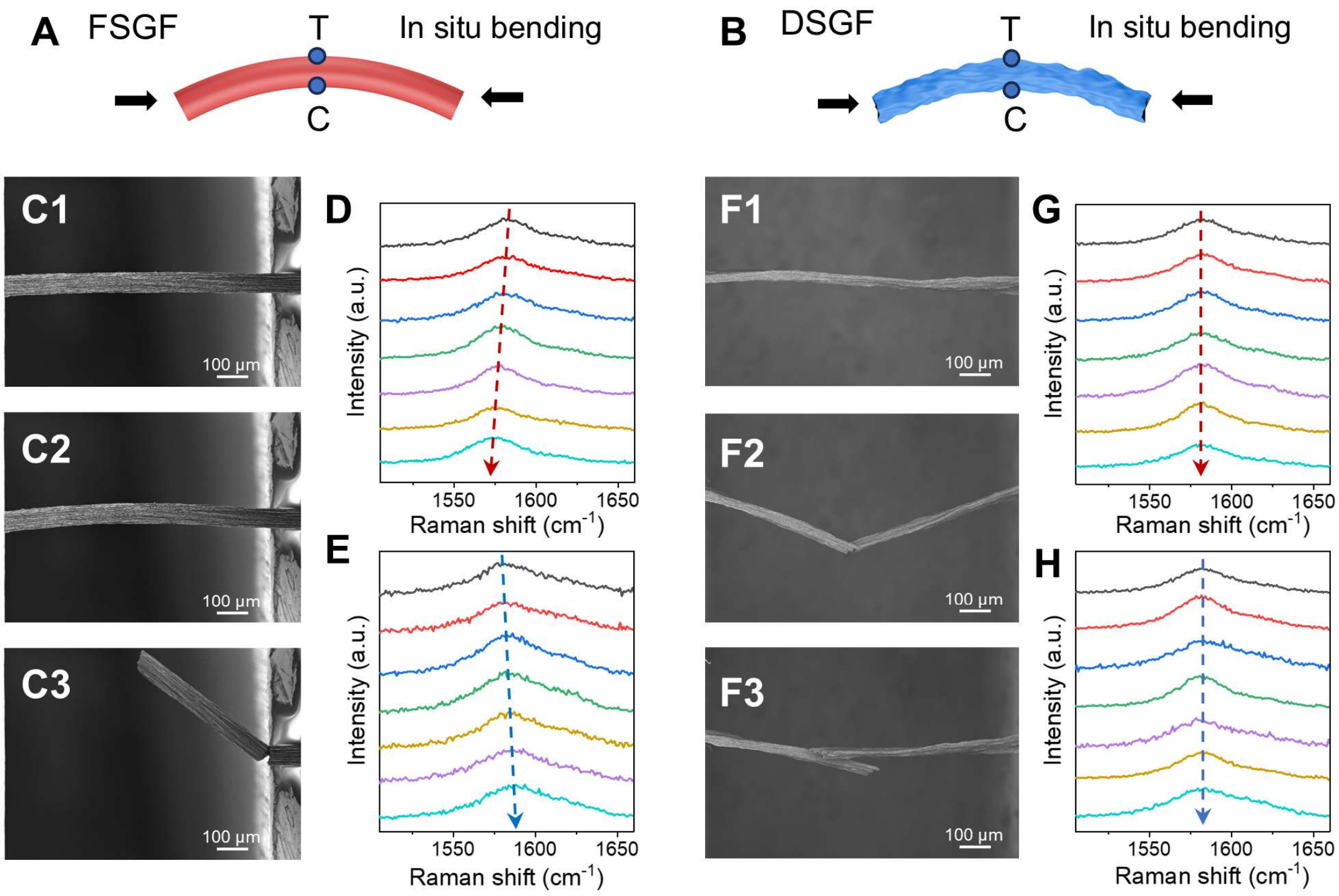

Fig. S39. In situ bending response of FSGF and DSGF. (**A** and **B**) Schematics of the bending process for FSGF (A) and DSGF (B). (**C1** to **C3**) Sequential in situ SEM images tracking the compressive/bending deformation of FSGF until failure. (**D** and **E**) In situ Raman G-band shifts monitored on the tensile side (D) and compressive side (E) of FSGF during bending. (**F1** to **F3**) Sequential in situ SEM images showing the bending and subsequent failure of DSGF. (**G** and **H**) In situ Raman G-band shifts monitored on the tensile side (G) and compressive side (H) of DSGF during bending, showing minimal shift.

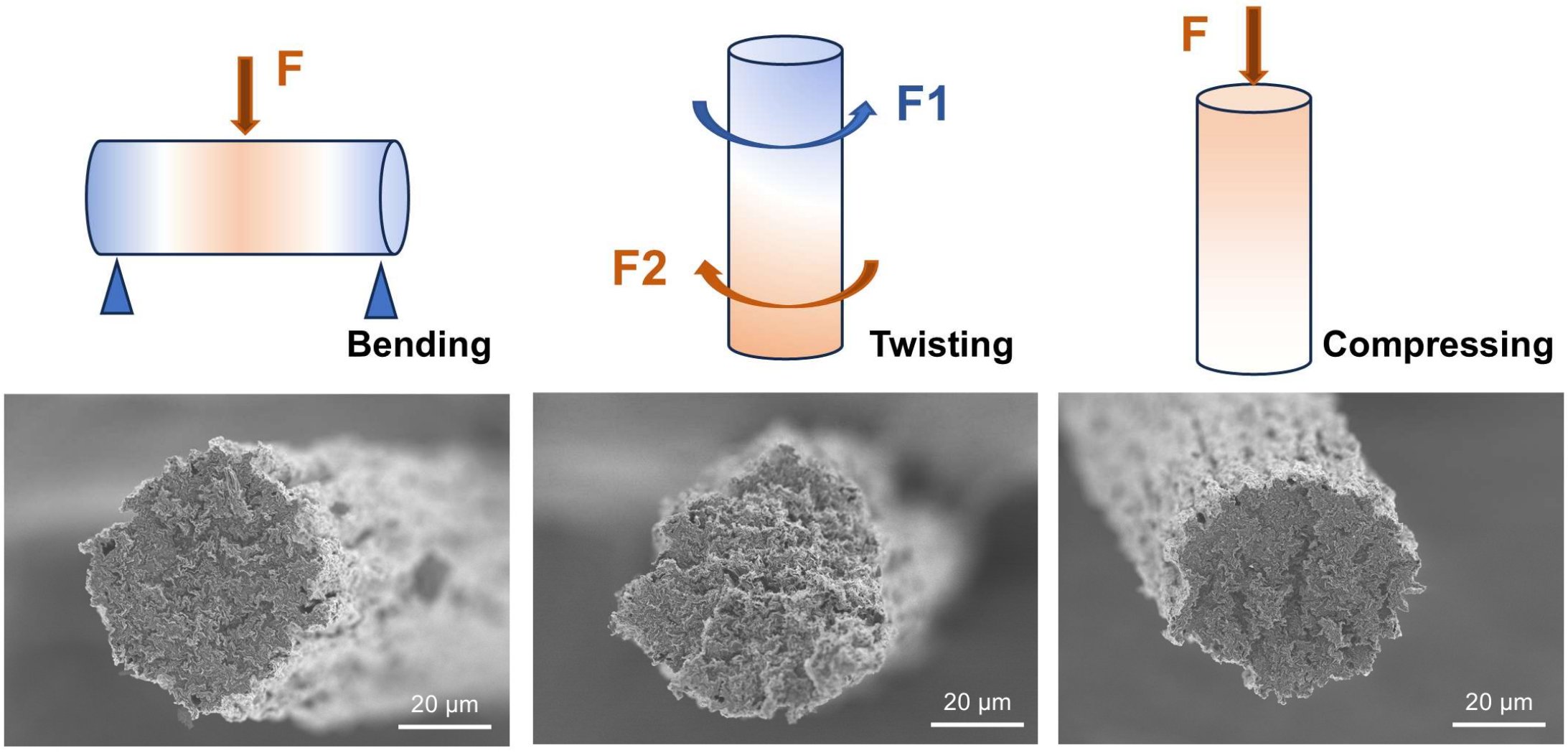

Fig. S40. Cross-sectional morphology of FSGF under different stress modes.

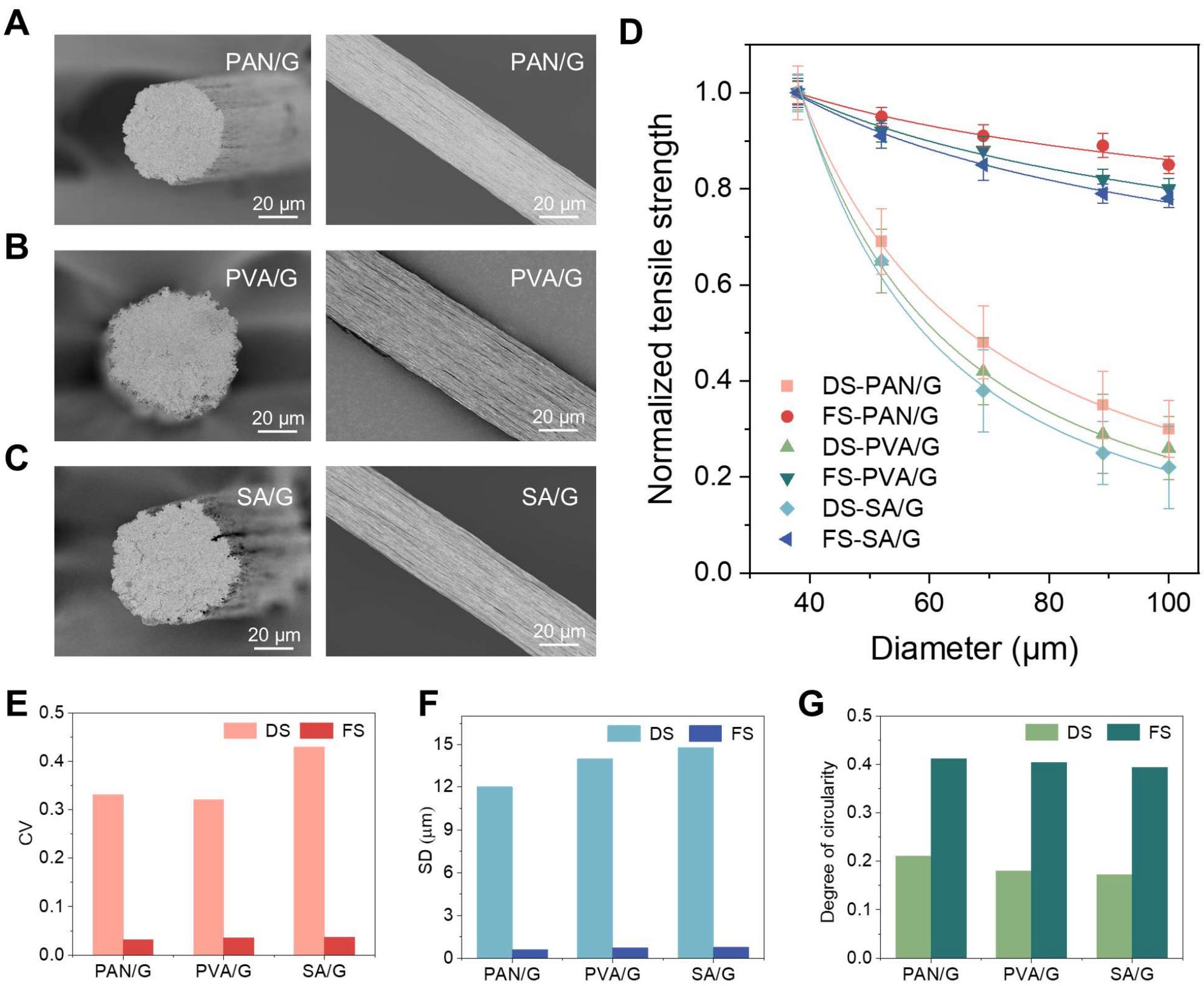

Fig. S41. Properties of GO-based composite fibers fabricated via multiscale fusion spinning. (**A** to **C**) Cross-sectional and lateral SEM images of fusion-spun composite fibers of GO with PAN (A), PVA (B), and SA (C). (**D**) Normalized tensile strength of fusion-spun GO composite fibers (FS) and direct-spun fibers (DS) as a function of diameter. (**E**) Coefficient of variation of tensile strength for FS and DS. (**F**) Standard deviation of fiber diameter along the axial direction for FS and DS. (**G**) Circularity of the fiber cross-section for FS and DS.

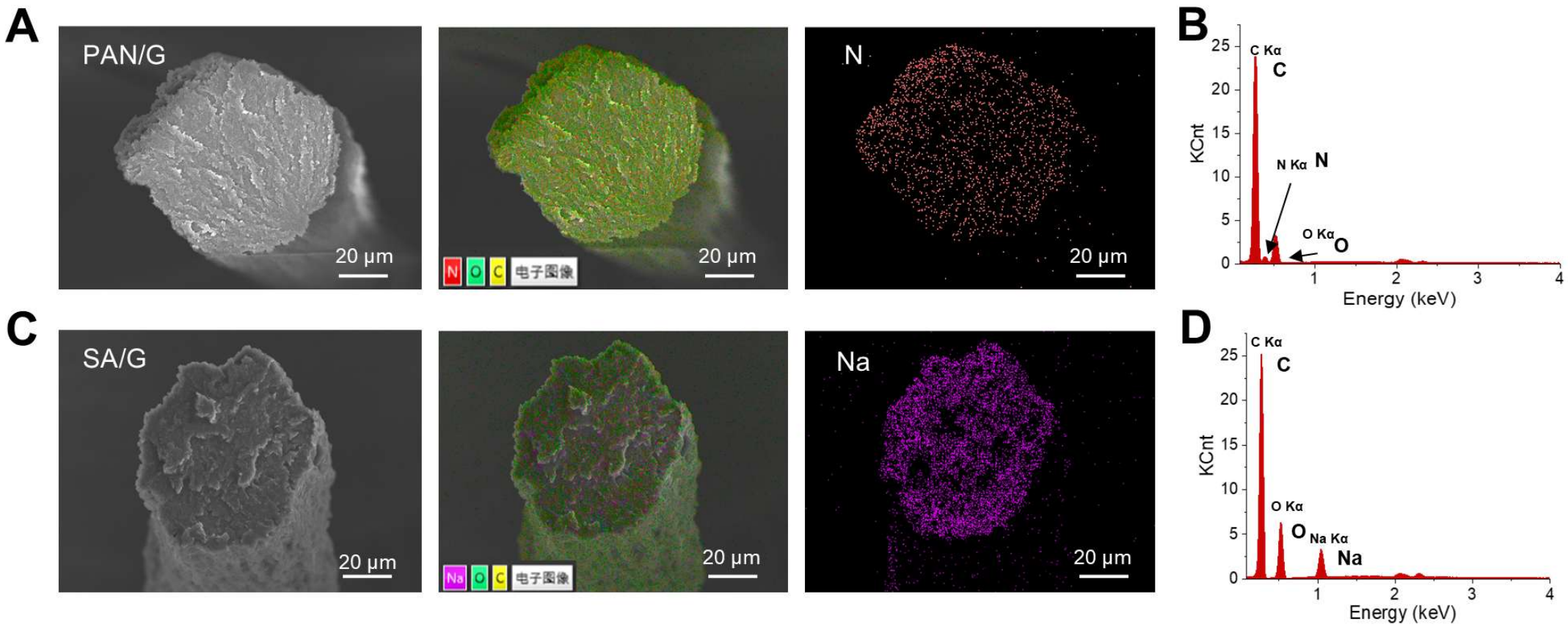

Fig. S42. Elemental analysis of GO composite fibers. (**A**) Overlay of EDS elemental maps for carbon (yellow), oxygen (green), and nitrogen (red) in a PAN/G composite fiber, confirming homogeneous incorporation of PAN. (**B**) Representative EDS spectrum of the PAN/G composite fiber. (**C**) Overlay of EDS elemental maps for carbon (yellow), oxygen (green), and sodium (purple) in a SA/G composite fiber, confirming homogeneous incorporation of SA. (**D**) Representative EDS spectrum of the SA/G composite fiber.

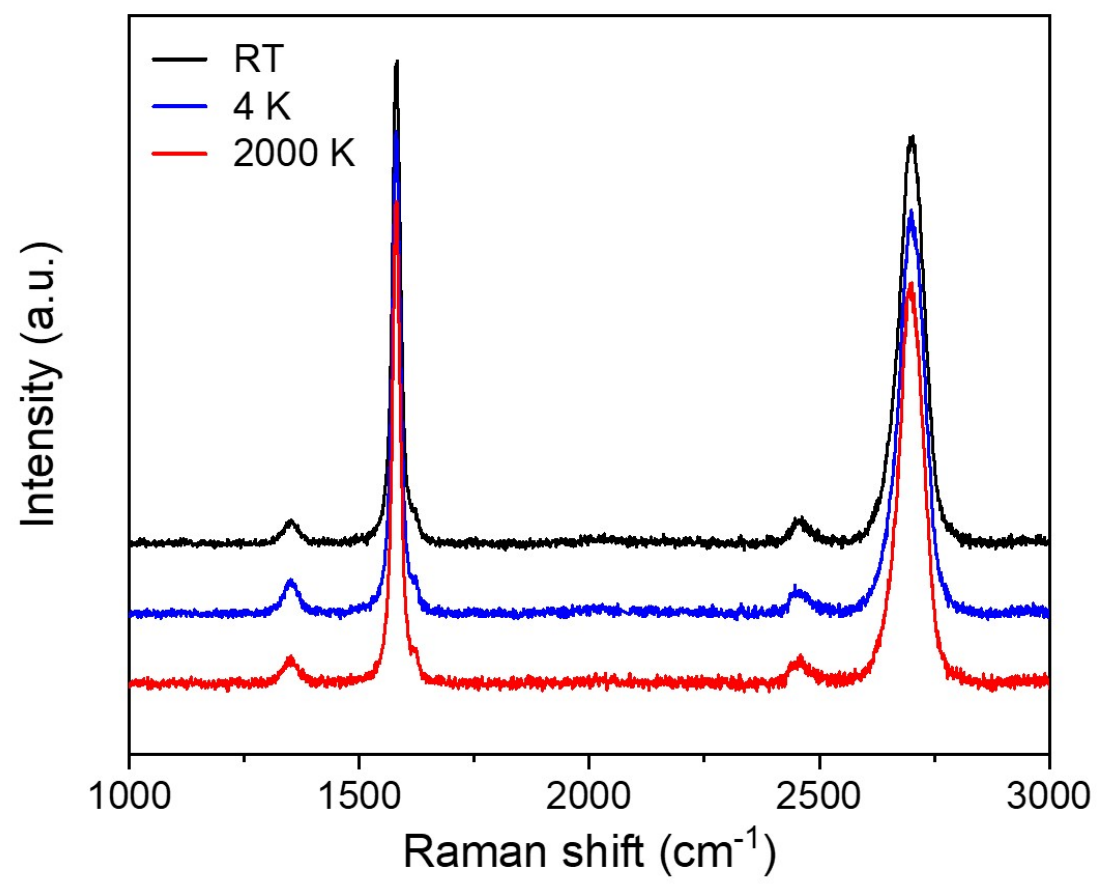

Fig. S43. Raman spectra of FSGF before and after exposure to 4 K and 2000 K.

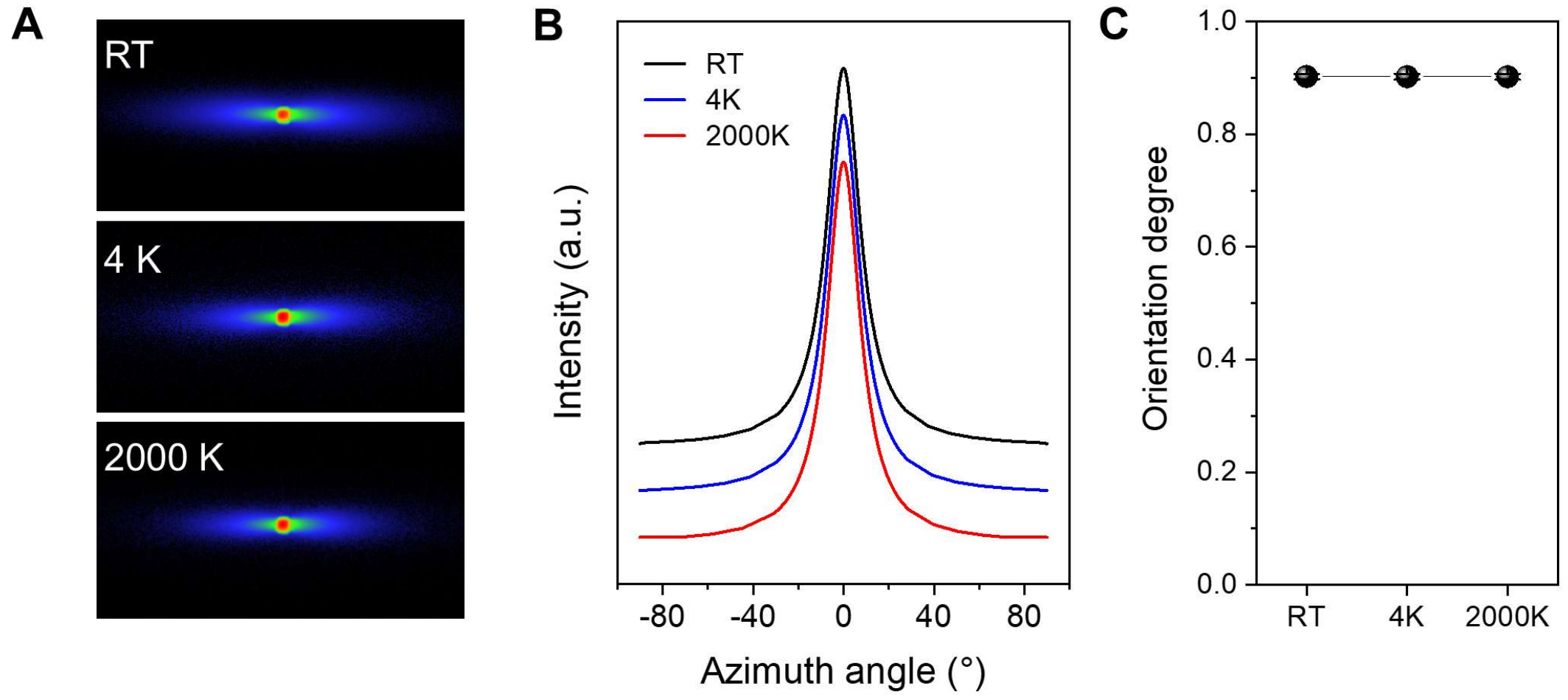

Fig. S44. Small-angle X-ray scattering (SAXS) characterization of FSGF at different temperatures. (**A**) Typical SAXS patterns after treatment at room temperature, 4 K, and 2000 K (Ar atmosphere). (**B**) SAXS peak integration at different treatment temperatures. (**C**) Orientation degree at different treatment temperatures.

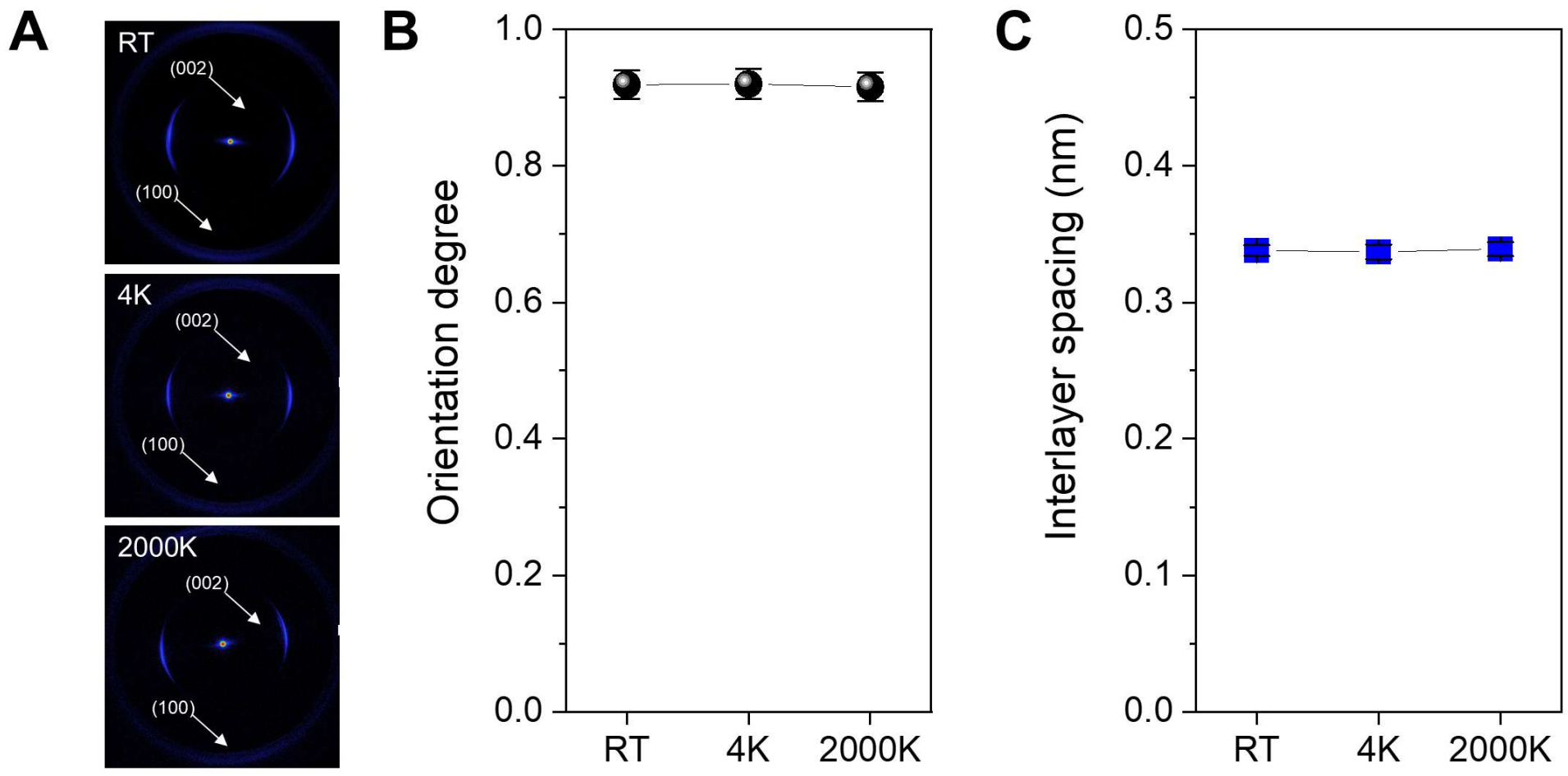

Fig. S45. Wide-angle X-ray scattering (WAXS) characterization of FSGF at different temperatures. (**A**) WAXS 2D patterns after treatment at room temperature, 4 K, and 2000 K (Ar atmosphere). (**B**) Orientation degree at different treatment temperatures. (**C**) Interlayer spacing at different treatment temperatures.

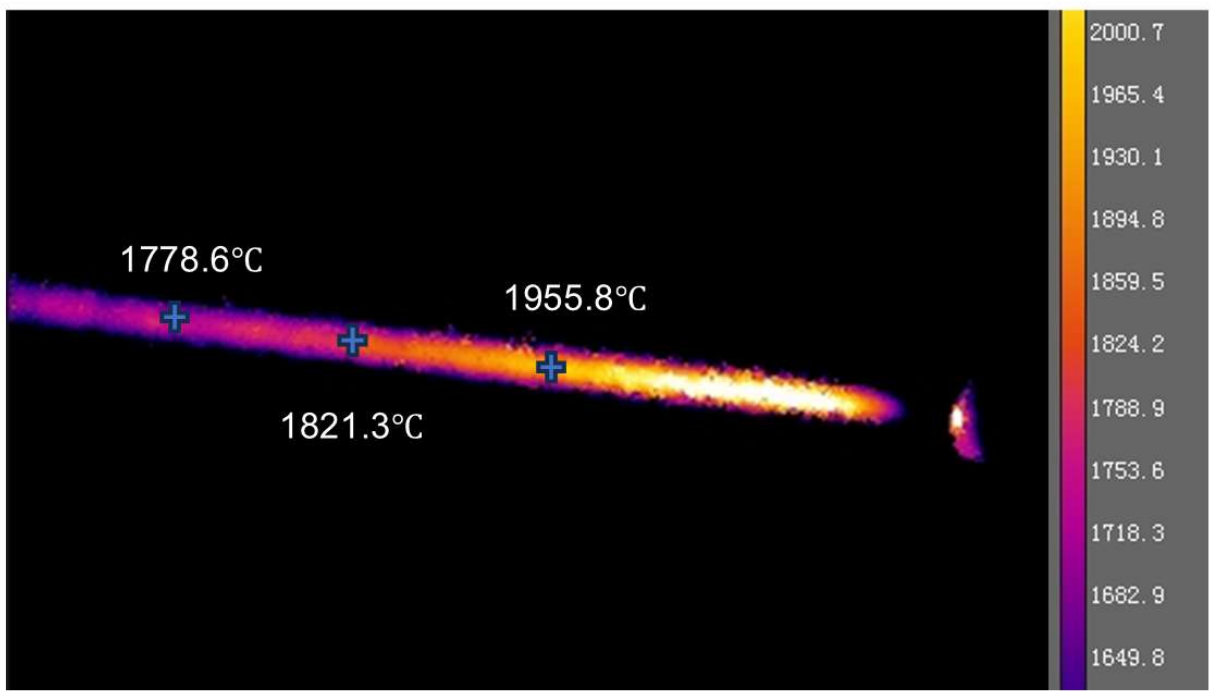

Fig. S46. Infrared image of a methane flame gun fire.

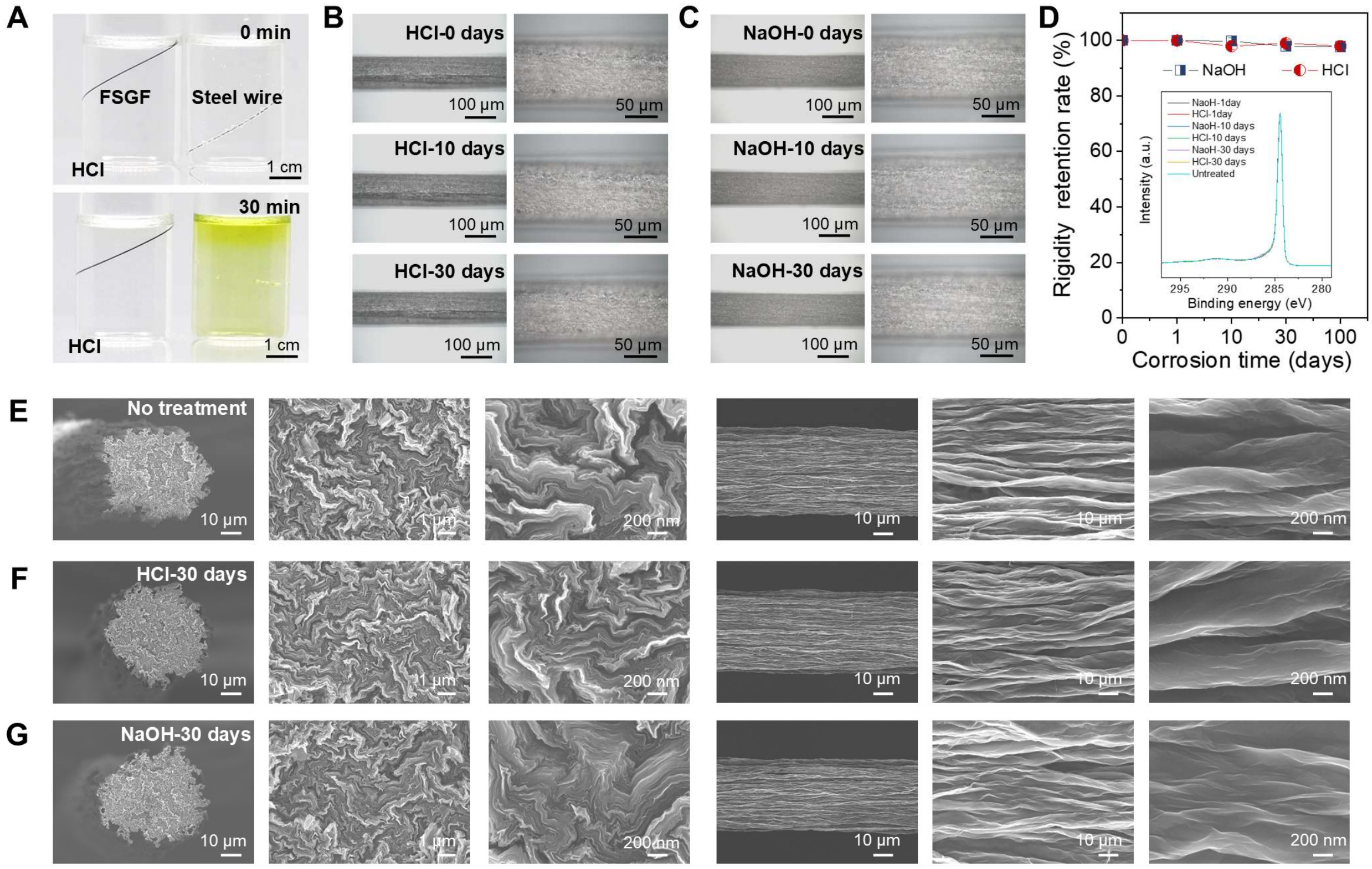

Fig. S47. Chemical stability of FSGF in corrosive environments. (**A**) Photograph comparing an FSGF (left) and an iron wire (right) immersed in hydrochloric acid (HCl). The iron wire rapidly produces bubbles and is completely corroded within 30 minutes, while the FSGF remains intact. (**B** and **C**) Optical micrographs of FSGF after immersion in HCl (B) and NaOH (C) solutions for different durations. (**D**) Retention of bending rigidity for FSGF after exposure to acid or base for varying times. Inset: corresponding XPS spectra. (**E** to **G**) SEM images of FSGF in axial and radial directions: untreated (E), after 30-day immersion in HCl (F), and after 30-day immersion in NaOH (G).

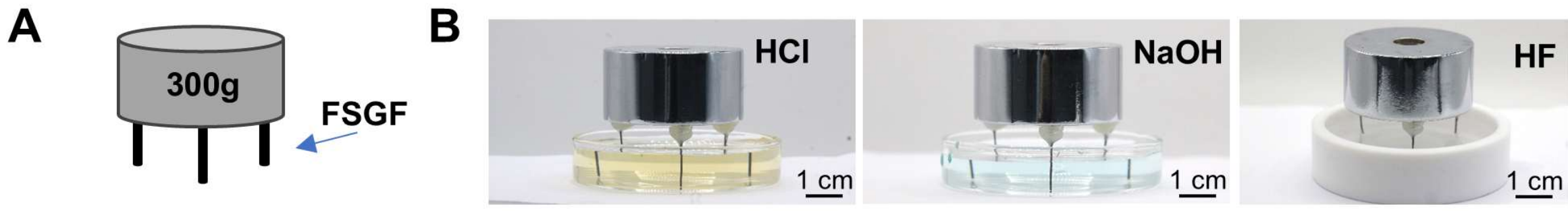

Fig. S48. Mechanical robustness of FSGF supporting loads in corrosive liquids. (**A**) Schematic of the load-supporting test setup. (**B**) Photographs demonstrating three FSGF supporting a weight while immersed in hydrochloric acid (HCl), sodium hydroxide (NaOH), and hydrofluoric acid (HF) solutions, respectively.

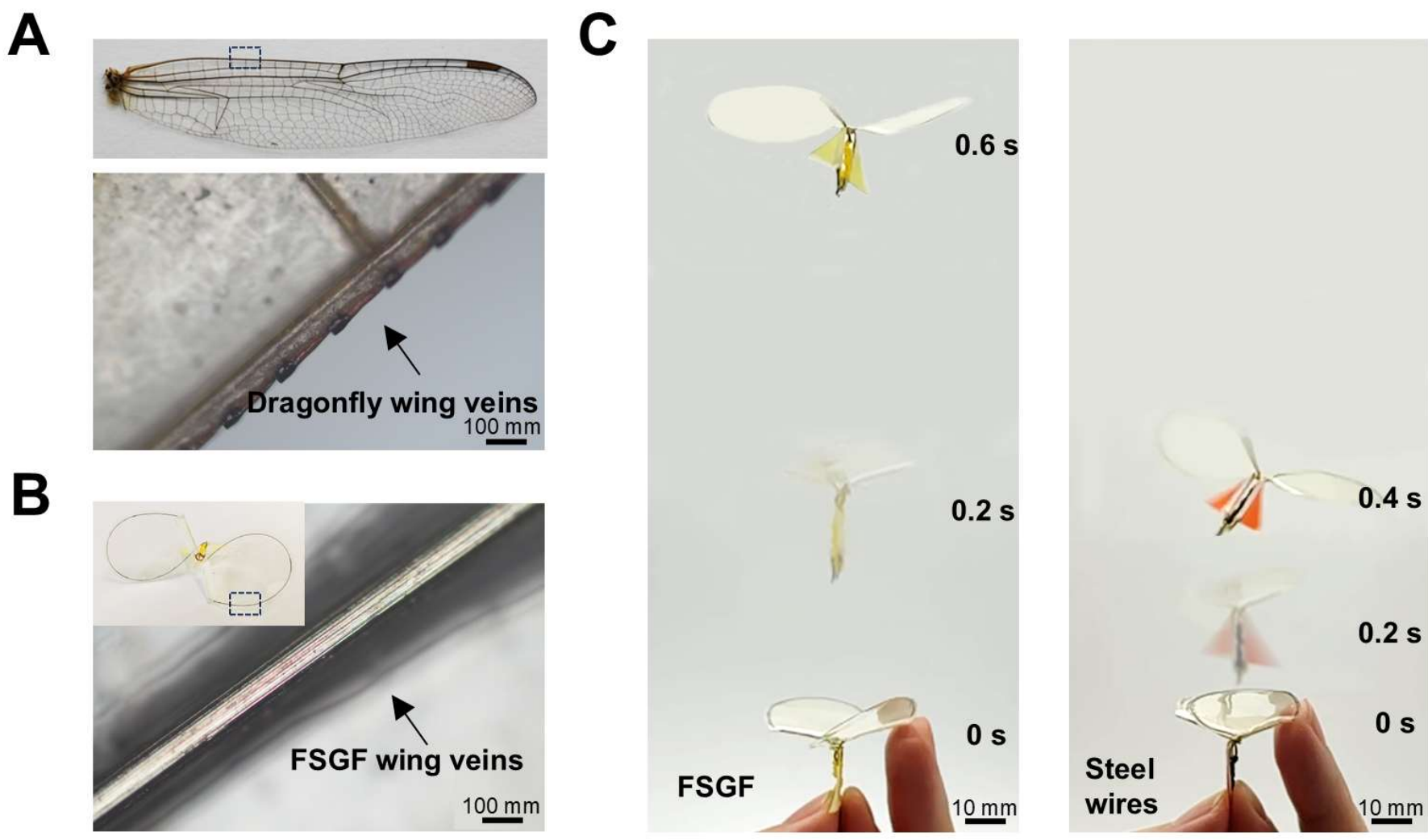

Fig. S49. Application of FSGF as biomimetic wing veins of micro-air-vehicles. (**A**) Photograph of a dragonfly wing specimen and a close-up of its wing venation. (**B**) Photograph of a micro-air-vehicle with FSGF wing veins and a close-up of the FSGF structure. (**C**) Strobe photograph comparing the flight of a micro-air-vehicle equipped with FSGF wing veins versus that with steel wing veins. Photos in (A) were taken by the authors.

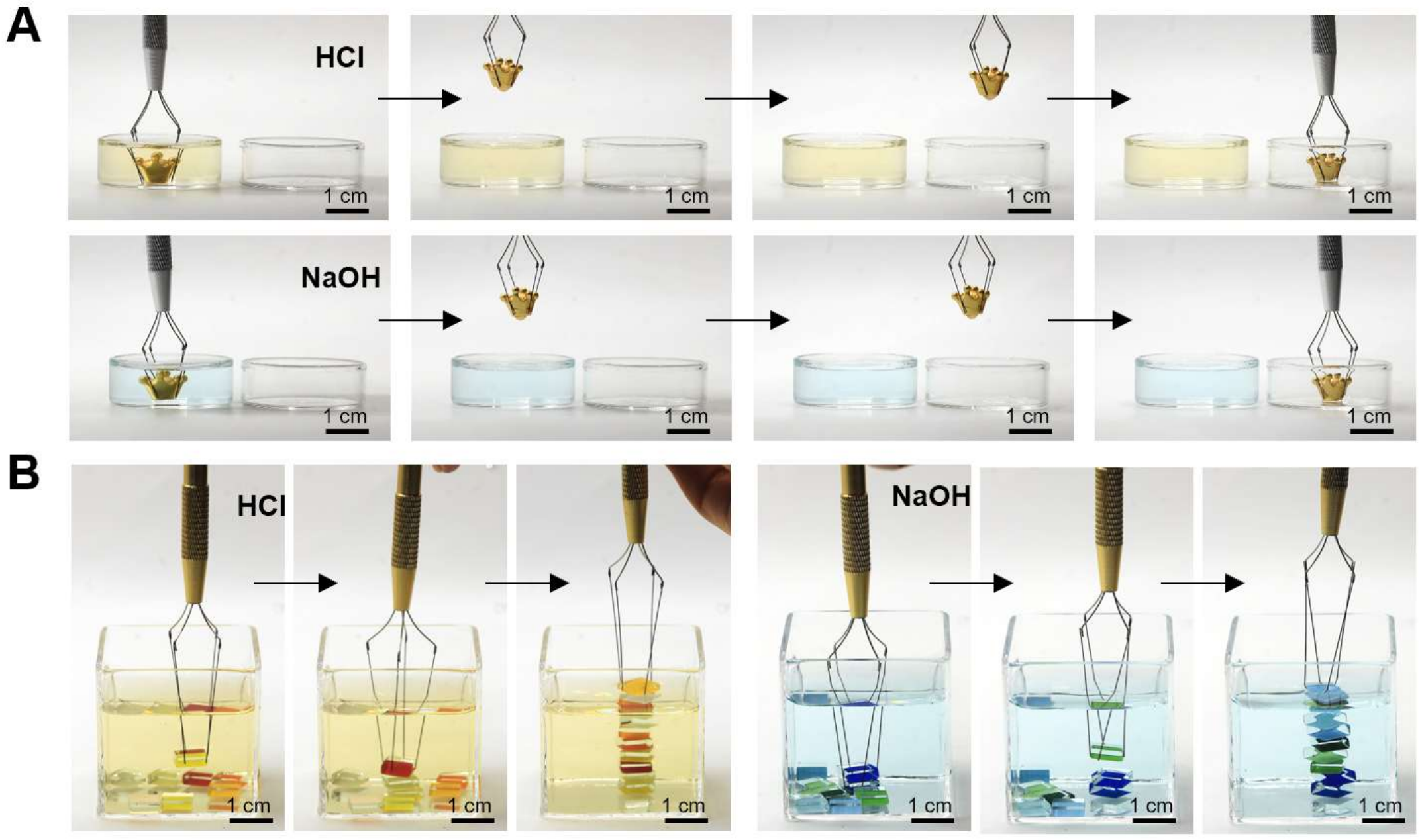

Fig. S50. Operation of FSGF-based robotic claw in corrosive environments. (**A**) Image sequence of the FSGF robotic claw grasping an object in strong acid and alkali solutions. (**B**) Demonstration of the FSGF robotic claw performing precision manipulation in strong acid and alkali solutions.

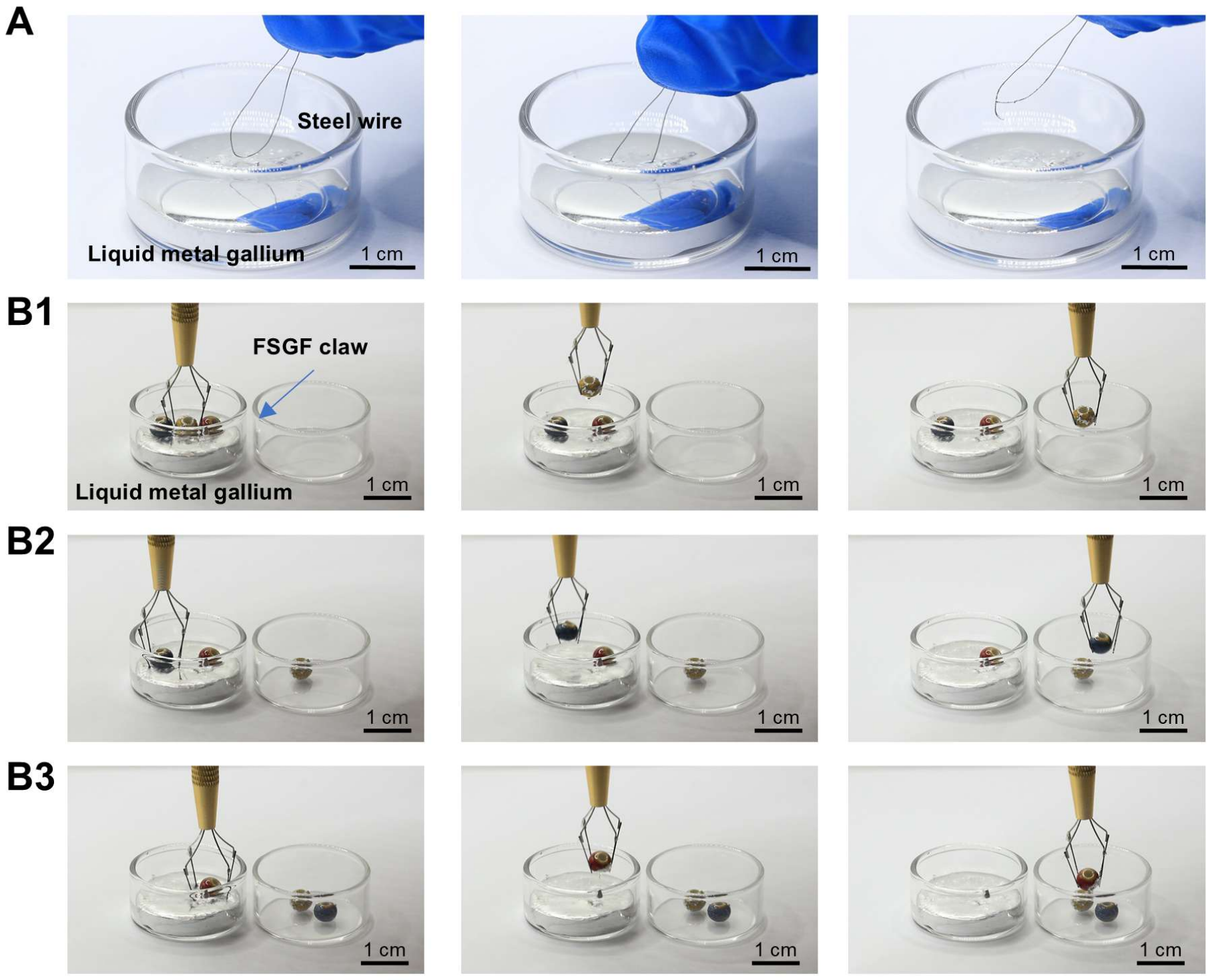

Fig. S51. Performance of FSGF robotic manipulator in liquid gallium. (**A**) Photograph showing the corrosion of an iron wire in liquid gallium metal. (**B1** to **B3**) Image sequence of an FSGF-based robotic claw repeatedly retrieving an object from liquid gallium.

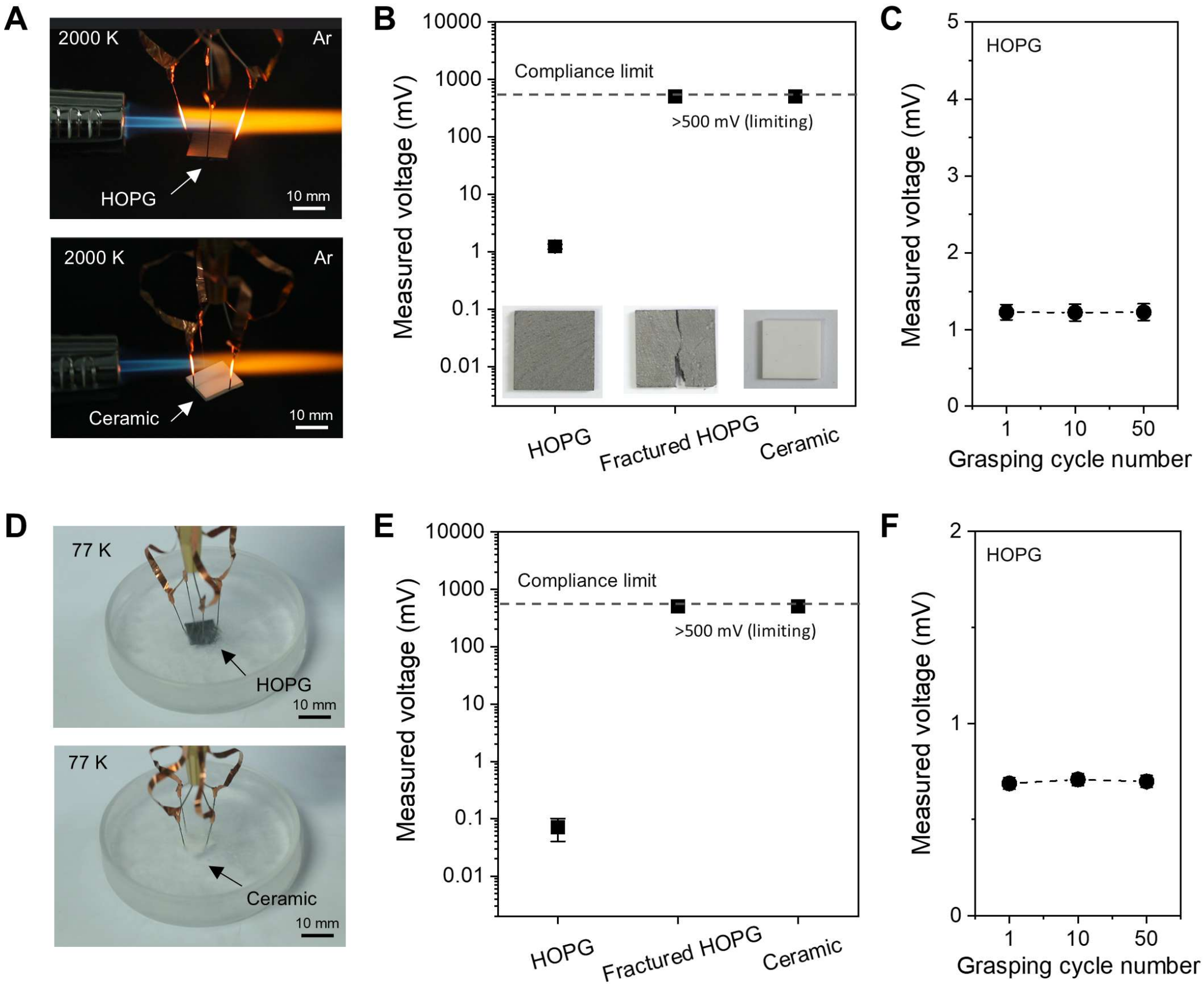

Fig. S52. Detection of grasped objects by the gripper under extreme high and low temperatures of 2000 K and 77 K. (**A**) Digital photograph of the robotic gripper grasping an object at 2000 K (Ar atmosphere). (**B**) Electrical signals measured from highly oriented pyrolytic graphite (HOPG), broken HOPG, and a ceramic block under a constant current of 1 mA using a four-wire configuration (the maximum voltage of the source meter is set to 500 mV) at 2000 K. (**C**) Electrical signals measured from HOPG after different numbers of repeated grasping cycles at 2000 K. (**D**) Digital photograph of the robotic gripper grasping an object at 77 K. (**E**) Electrical signals measured from HOPG, broken HOPG, and a ceramic block under the same electrical conditions as in (B) but at 77 K. (**F**) Electrical signals measured from HOPG after different numbers of repeated grasping cycles at 77 K.

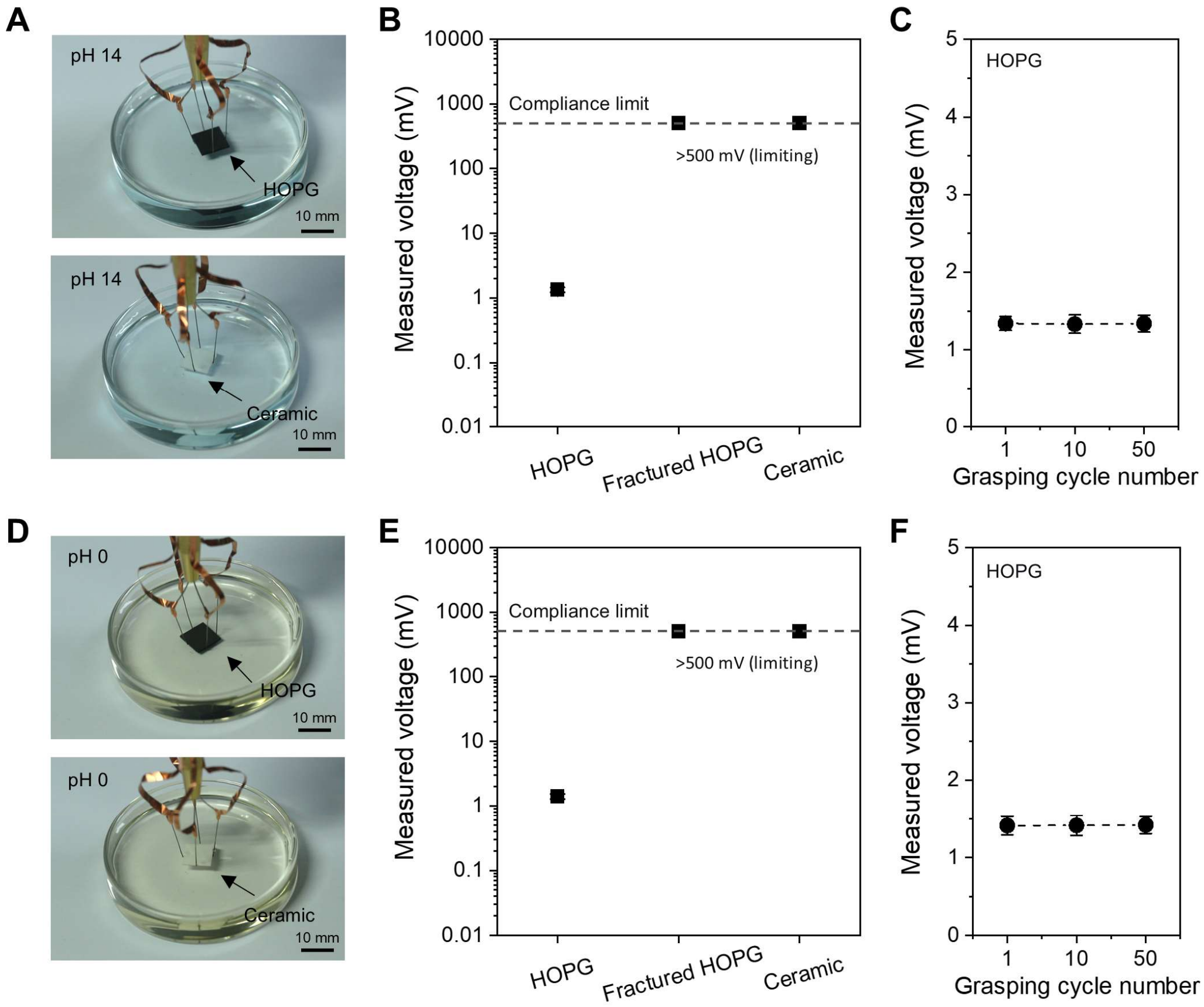

Fig. S53. Detection of grasped objects by the gripper under extreme acidic and alkaline conditions of pH 0 and pH 14. (**A**) Digital photograph of the robotic gripper grasping an object at pH 14. (**B**) Electrical signals measured from highly oriented pyrolytic graphite (HOPG), broken HOPG, and a ceramic block under a constant current of 1 mA using a four-wire configuration (the maximum voltage of the source meter is set to 500 mV) at pH 14. (**C**) Electrical signals measured from HOPG after different numbers of repeated grasping cycles at pH 14. (**D**) Digital photograph of the robotic gripper grasping an object at pH 0. (**E**) Electrical signals measured from HOPG, broken HOPG, and a ceramic block under the same electrical conditions as in (B) but at pH 0. (**F**) Electrical signals measured from HOPG after different numbers of repeated grasping cycles at pH 0.

Table S1. Comparison of key properties between previous representative works and FSGF in this work.

Work (Year)	Diameter (μm)	Tensile strength (GPa)	Young's modulus (GPa)	Bending rigidity (N μm^2)	Independent structural component?
2011 (ref.12)	40	0.14	3	3.77×10^2	No
2013 (ref.13)	20	0.5	8	6.28×10^1	No
2016 (ref.10)	8	2.2	400	8.04×10^1	No
2020 (ref.14)	7	3.4	340	4.01×10^1	No
2024 (ref.15)	8	2.5	901	1.81×10^2	No
2026 (ref.16)	4	5.19	529	6.65	No
This work	100	3	330	1.62×10^6	Yes

Table S2. Performance comparison of different fibers

Fiber	Type	Diameter (μm)	Density (g cm^{-3})	Tensile strength (MPa)	Young's modulus (GPa)	Specific tensile strength (MPa/g cm^{-3})	Specific bending rigidity (N μm^2/g cm^{-3})
FSGF	This work	70	1.9	3146	343	1.66×10^3	2.21×10^5
	This work	86	1.9	3121	340	1.64×10^3	4.94×10^5
	This work	100	1.9	3028	330	1.59×10^3	8.52×10^5
Carbon fibers	T300	7	1.76	3530	230	2.01×10^3	1.54×10^1
	T800	5	1.80	5880	294	3.27×10^3	5.01
	T1100	5	1.81	7000	324	3.87×10^3	5.49
	M40J	5	1.75	4400	377	2.51×10^3	6.61
	M60J	5	1.93	3820	588	1.98×10^3	9.34
Metal fibers	Q235	100	7.85	450	210	5.73×10^1	1.31×10^5
	304	100	7.90	600	190	7.59×10^1	1.18×10^5
	316	100	8.00	680	193	8.50×10^1	1.18×10^5
	Copper	100	8.92	358	116	4.01×10^1	6.38×10^4
	Aluminum	100	2.72	108	68	3.97×10^1	1.23×10^5
Polymer fibers	Nylon 6	30	1.13	80	2.5	7.08×10^1	8.79×10^1
	Nylon 66	50	1.14	90	3	7.89×10^1	8.07×10^2
	Polyester Fibers	20	1.39	800	3	5.76×10^2	1.69×10^1
	Polypropylene Fibers	30	0.92	500	3	5.43×10^2	1.30×10^2
	Nylon24	30	1.06	90	3.5	8.49×10^1	1.31×10^2
	Nylon82	50	1.15	100	4.5	8.70×10^1	1.20×10^3
	Kevlar® 29	12	1.44	2900	70	2.01×10^3	4.95×10^1
	Kevlar® 49	12	1.45	2800	125	1.93×10^3	8.77×10^1
Glass fibers	Quartz glass fibers	15	2.30	1100	78	4.78×10^2	8.42×10^1
	E-type glass fibers	20	2.55	2500	73	9.80×10^2	2.25×10^2
	D-type glass fibers	20	2.70	2000	70	7.41×10^2	2.04×10^2
	S-type glass fibers	20	2.50	4000	90	1.60×10^3	2.83×10^2
CNT fibers	2020	8.9	1.50	4200	260	2.80×10^3	5.34×10^1
	2013	9	1.30	1000	150	7.69×10^2	3.71×10^1
	2022	10	1.50	4620	302	3.08×10^3	9.88×10^1
Ceramic fibers	SiC	20	3.20	600	350	1.88×10^2	8.59×10^2
	Polycrystalline alumina (Du Pont)	15	2.70	1100	200	4.07×10^2	1.84×10^2
	Amorphous 85 wt% Al_2O_3 - 15 wt% SiO_2 Fibers (Sumitomo Chemical)	15	2.40	1500	100	6.25×10^2	1.03×10^2
	Al_2O_3-SiO_2 Fibers	20	2.50	1600	150	6.40×10^2	4.71×10^2

Table S3. Comparison of required mechanical properties for three robotic components and the actual properties of FSGF.

Application	Required strength (MPa)	Required bending rigidity (N μm^2)	FSGF strength (MPa)	FSGF bending rigidity (N μm^2)
Wing vein (100 μm in diameter)	160	1.14×10^6	3028	1.6×10^6
Spider foot (200 μm in diameter)	10.4	6.49×10^6	2811	2.51×10^7
Gripper claw (200 μm in diameter)	484	2.18×10^7	2811	2.51×10^7

Movie S1.
Continuous production process of fused graphene oxide fibers.

Movie S2.
Comparison of ice insertion time between FSGF and metal fibers demonstrating the superior thermal conductivity of FSGF.

Movie S3.
High wind resistance and flight height comparisons of FSGF-based wing and steel wire-based wing.

Movie S4.
Efficient walking in strong acid and alkali of FSGF-integrated spider-mimic robot.

Movie S5.
Precise manipulation of objects in strong acid and alkali realized by FSGF-integrated robotic claw.

Movie S6.
Robust manipulation of FSGF-integrated robotic claw in liquid gallium.

Movie S7.
Robust manipulation of FSGF-integrated robotic claw under ultra-high and ultra-low temperatures.
